# Supplementary figures and images for: Detection of R.1 lineage severe acute respiratory syndrome coronavirus 2 (SARS-CoV-2) with spike protein W152L/E484K/G769V mutations in Japan
Source: PLoS Pathog. 2021 Jun 7;17(6):e1009619. doi: 10.1371/journal.ppat.1009619 (PMC8238201; doi:10.1371/journal.ppat.1009619)

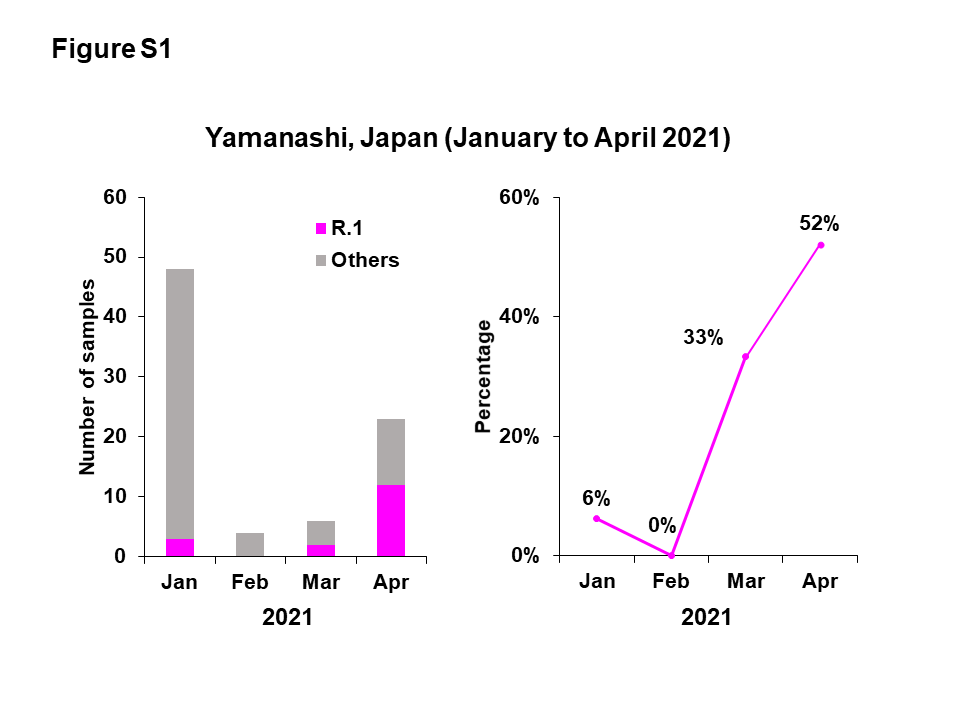

Supplement: S1 Fig — (A) The number of R.1 lineage SARS-CoV-2 samples identified by TaqMan assay and whole genome analysis during the period from January to April 2021. The pink color indicates R.1 lineage strains, and the gray color indicates other strains. (B) The percentage of SARS-CoV-2 strains detected over the period from January to April 2021 that belong to the R.1 lineage. (TIF) [file ppat.1009619.s001.tif]

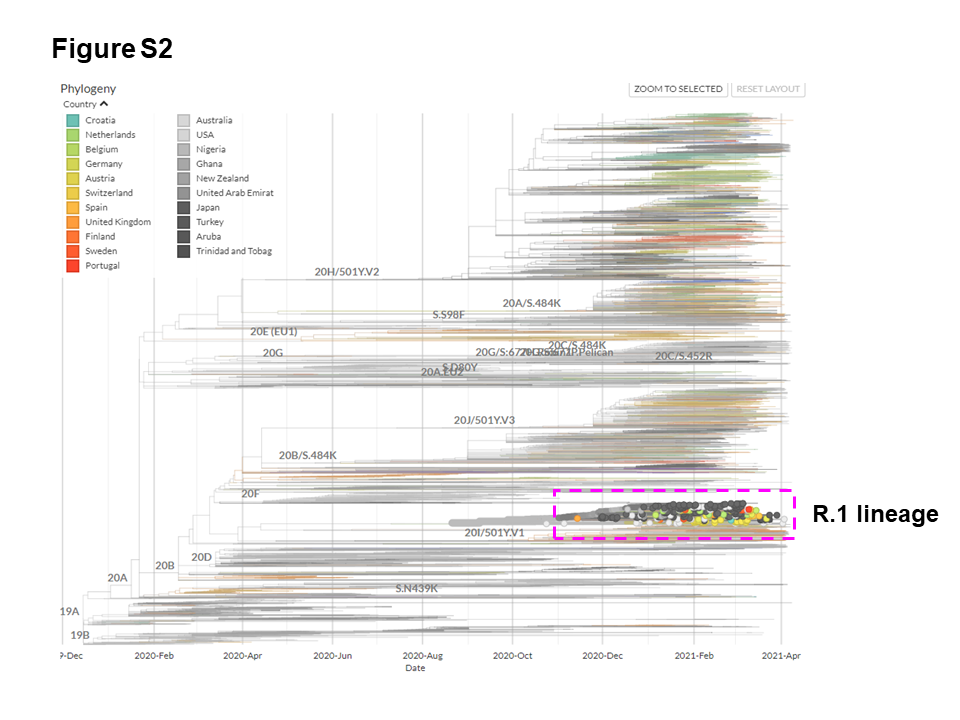

Supplement: S2 Fig — The dotted pink line shows a monophyletic clade containing the SARS-CoV-2 R.1 lineage. (TIF) [file ppat.1009619.s002.tif]
