# Supplementary material for: Detection of R.1 lineage severe acute respiratory syndrome coronavirus 2 (SARS-CoV-2) with spike protein W152L/E484K/G769V mutations in Japan
Source: PLoS Pathog. 2021 Jun 7;17(6):e1009619. doi: 10.1371/journal.ppat.1009619 (PMC8238201; doi:10.1371/journal.ppat.1009619)
Supplement: S1 File — (PDF) [file ppat.1009619.s003.pdf]

We gratefully acknowledge the following Authors from the Originating laboratories responsible for obtaining the specimens, as well as the Submitting laboratories where the genome data were generated and shared via GISAID, on which this research is based.

All Submitters of data may be contacted directly via www.gisaid.org

Authors are sorted alphabetically.

Accession ID	Originating Laboratory	Submitting Laboratory	Authors
EPI_ISL_1009535, EPI_ISL_1009564	Erie County Public Health (ECPHL)	University at Buffalo Genomics and Bioinformatics Core	Jonathan Bard, Natalie Lamb, Alyssa Pohlman, Brandon Marzullo, Amanda Boccolucci, Norma Nowak, Donald Yergeau, Jennifer Surtees
EPI_ISL_1010709	New South Wales Health Pathology Royal Prince Alfred Hospital	Microbiology RPAH	Foster, C.; Au, J.; Ruiz Silva, M.; Deveson, I.; Bull, R.; Van Hal, S.; Rawlinson, W.
EPI_ISL_1015618	Maryland Public Health Laboratory	Maryland Public Health Laboratory	Maryland Department of Health Laboratories Administration
EPI_ISL_1016417	ALBANY MEDICAL CENTER HOSPITAL CLINICAL LABORATORIES	Wadsworth Center, New York State Department of Health	Kirsten St. George, Daryl M. Lamson, Alexis Russel, Matthew Shudt, Melissa A Leisner, Jonathan Plitnick, Navjot Singh, John Kelly, Erasmus Schneider, Erica Lasek-Nesselquist
EPI_ISL_1018072, EPI_ISL_1018081, EPI_ISL_1018098	Immunology, Noguchi Memorial Institute for Medical Research	Immunology, Noguchi Memorial Institute for Medical Research	Adu,B., Egyir,B., Kumordjie,S., Agbodzi,B., Yeboah,C., Mohktar,Q., Oteng,F., Owusu-Nyantakyi,C., Asare,K.M., Appiah-Kubi,J., Adusei-Poku,M.A., Odoom,J.K., Ampofo,W.K., Bonney,J.K.
EPI_ISL_1020347	MEPHI, Aix Marseille University	MEPHI, Aix Marseille University	Anthony LEVASSEUR
EPI_ISL_1035598, EPI_ISL_1035785	Dutch COVID-19 response team	National Institute for Public Health and the Environment (RIVM)	Adam Meijer, Harry Vennema, Dirk Eggink, Jeroen Cremer, Sharon van den Brink, Bas van der Veer, AnneMarie van den Brandt, Florian Zwagemaker, Dennis Schmitz, Chantal Reusken, on behalf of the national COVID-19 response team
EPI_ISL_1036299, EPI_ISL_1036300, EPI_ISL_1036301, EPI_ISL_1036302	Johns Hopkins Hospital Department of Pathology	Johns Hopkins Hospital Department of Pathology	C. Paul Morris, Chun Huai Luo, Adannaya Amadi, Matthew Schwartz, Nicholas Gallagher, Heba H. Mostafa
EPI_ISL_1036609	Department of Clinical Microbiology	GIGA Medical Genomics	Keith Durkin, Maria Artesi, Sébastien Bontems, Raphaël Boreux, Bouchra Boujemla, Nathalie Renotte, Cécile Meex, Pierrette Melin, Marie-Pierre Hayette, Vincent Bours
EPI_ISL_1040514, EPI_ISL_1040515, EPI_ISL_1040516, EPI_ISL_1040517, EPI_ISL_1040518, EPI_ISL_1040534	University of Liège COVID-19 testing center	GIGA Medical Genomics	Keith Durkin, Maria Artesi, Bouchra Boujemla, Nathalie Renotte, Cécile Meex, Sébastien Bontems, Fabrice Bureau, Laurent Gillet, Wouter Coppieters, Marie-Pierre Hayette, Vincent Bours
EPI_ISL_1040885	NYU Langone Health	Departments of Pathology and Medicine, New York University School of Medicine	Adriana Heguy, Dacia Dimartino, Emily Guzman, Christian Marier, Peter Meyn, Sitharam Ramaswami, Gael Westby, Paul Zappile, Yutong Zhang, Paolo Cotzia, Guiqing Wang
EPI_ISL_1041417, EPI_ISL_1041420, EPI_ISL_1041478, EPI_ISL_1041479, EPI_ISL_1041489, EPI_ISL_1041669, EPI_ISL_1041687, EPI_ISL_1041690	Pandemic Response Lab - NYC	Pandemic Response Lab, R&D	Henry Lee, Michael Hammerling, Melissa Hopkins, Cybill del Castillo, William Ward, Pradeep Bugga, Haiping Hao, Jon Laurent
EPI_ISL_1041945, EPI_ISL_1041946, EPI_ISL_1041947, EPI_ISL_1041948	Tokyo Metropolitan Institute of Public Health	Tokyo Metropolitan Institute of Public Health	Masaki Hayashi, Takako Yamazaki, Ayano Hotta, Souichi Yoshikawa, Tomoki Igarashi, Kayo Hagino, Maki Kanda, Kenji Iida, Mami Nagashima,Takushi Fujiwara, Takashi Chiba, Kenji Sadamasu
EPI_ISL_1042980, EPI_ISL_1043085, EPI_ISL_1043099	Lighthouse Lab in Alderley Park	Wellcome Sanger Institute for the COVID-19 Genomics UK (COG-UK) Consortium	Jacquelyn Wynn, Mairead Hyland, The Lighthouse Lab in Alderley Park and Alex Alderton, Roberto Amato, Jeffrey Barrett, Sonia Goncalves, Ewan Harrison, David K. Jackson, Ian Johnston, Dominic Kwiatkowski, Cordelia Langford, John Sillitoe on behalf of the Wellcome Sanger Institute COVID-19 Surveillance Team
EPI_ISL_1065857	Department of Virus and Microbiological Special Diagnostics, Statens Serum Institut, Copenhagen, Denmark	Aalborg University	Danish Covid-19 Genome Consortium
EPI_ISL_1072166	Lighthouse Lab in Alderley Park	Wellcome Sanger Institute for the COVID-19 Genomics UK (COG-UK) Consortium	Jacquelyn Wynn, Mairead Hyland, The Lighthouse Lab in Alderley Park and Alex Alderton, Roberto Amato, Jeffrey Barrett, Sonia Goncalves, Ewan Harrison, David K. Jackson, Ian Johnston, Dominic Kwiatkowski, Cordelia Langford, John Sillitoe on behalf of the Wellcome Sanger Institute COVID-19 Surveillance Team
EPI_ISL_1072370	Lighthouse Lab in Milton Keynes	Wellcome Sanger Institute for the COVID-19 Genomics UK (COG-UK) Consortium	The Lighthouse Lab in Milton Keynes and Alex Alderton, Roberto Amato, Jeffrey Barrett, Sonia Goncalves, Ewan Harrison, David K. Jackson, Ian Johnston, Dominic Kwiatkowski, Cordelia Langford, John Sillitoe on behalf of the Wellcome Sanger Institute COVID-19 Surveillance Team
EPI_ISL_1072967, EPI_ISL_1072968, EPI_ISL_1072972, EPI_ISL_1072973, EPI_ISL_1072977, EPI_ISL_1072978, EPI_ISL_1072983, EPI_ISL_1072984	Tokyo Metropolitan Institute of Public Health	Tokyo Metropolitan Institute of Public Health	Masaki Hayashi, Takako Yamazaki, Mami Nagashima,Takushi Fujiwara, Takashi Chiba, Kenji Sadamasu
EPI_ISL_1074484, EPI_ISL_1075422, EPI_ISL_1078109, EPI_ISL_1078407, EPI_ISL_1078490	Houston Methodist Hospital	Houston Methodist Hospital	S. Wesley Long, Randall J. Olsen, Paul A. Christensen, Sishir Subedi, Robert Olson, James J. Davis, Matthew Ojeda Saavedra, Prasanti Yerramilli, Layne Pruitt, Kristina Reppond, Madison N. Shyer, Jessica Cambric, Ilya J. Finkelstein, Jimmy Gollihar, and James M. Musser
EPI_ISL_1078594, EPI_ISL_1078597	Tokyo Metropolitan Institute of Public Health	Tokyo Metropolitan Institute of Public Health	Masaki Hayashi, Takako Yamazaki, Ayano Hotta, Souichi Yoshikawa, Tomoki Igarashi, Kayo Hagino, Maki Kanda, Kenji Iida, Mami Nagashima,Takushi Fujiwara, Takashi Chiba, Kenji Sadamasu
EPI_ISL_1078693, EPI_ISL_1078815, EPI_ISL_1078877, EPI_ISL_1079558, EPI_ISL_1080031, EPI_ISL_1080063, EPI_ISL_1080314	Houston Methodist Hospital	Houston Methodist Hospital	S. Wesley Long, Randall J. Olsen, Paul A. Christensen, Sishir Subedi, Robert Olson, James J. Davis, Matthew Ojeda Saavedra, Prasanti Yerramilli, Layne Pruitt, Kristina Reppond, Madison N. Shyer, Jessica Cambric, Ilya J. Finkelstein, Jimmy Gollihar, and James M. Musser
EPI_ISL_1080341	NJ Rapid Response Teams	NJ_PHEL	Lindsey Bodnar, Shiv Verma, Dana Woell, Byeong Jeong
EPI_ISL_1081224, EPI_ISL_1081245	Maryland Public Health Laboratory	Maryland Public Health Laboratory	Maryland Department of Health Laboratories Administration
EPI_ISL_1082634, EPI_ISL_1082653	Lighthouse Lab in Milton Keynes	Wellcome Sanger Institute for the COVID-19 Genomics UK (COG-UK) Consortium	The Lighthouse Lab in Milton Keynes and Alex Alderton, Roberto Amato, Jeffrey Barrett, Sonia Goncalves, Ewan Harrison, David K. Jackson, Ian Johnston, Dominic Kwiatkowski, Cordelia Langford, John Sillitoe on behalf of the Wellcome Sanger Institute COVID-19 Surveillance Team
EPI_ISL_1089819, EPI_ISL_1090032, EPI_ISL_1090113, EPI_ISL_1090139, EPI_ISL_1090140, EPI_ISL_1090170, EPI_ISL_1090172, EPI_ISL_1090177, EPI_ISL_1090245	Dutch COVID-19 response team	National Institute for Public Health and the Environment (RIVM)	Adam Meijer, Harry Vennema, Dirk Eggink, Jeroen Cremer, Sharon van den Brink, Bas van der Veer, AnneMarie van den Brandt, Florian Zwagemaker, Dennis Schmitz, Chantal Reusken, on behalf of the national COVID-19 response team
EPI_ISL_1091929, EPI_ISL_1091931, EPI_ISL_1091932, EPI_ISL_1091938, EPI_ISL_1091984	Johns Hopkins Hospital Department of Pathology	Johns Hopkins Hospital Department of Pathology	C. Paul Morris, Chun Huai Luo, Adannaya Amadi, Matthew Schwartz, Nicholas Gallagher, Heba H. Mostafa
EPI_ISL_1094200	VT Dept. of Health Laboratory	Respiratory Viruses Branch, Division of Viral Diseases,	Krista Queen, Yan Li, Ying Tao, Jing Zhang, Anna Uehara, Anna Montmayeur, Clinton R. Paden, Peter W. Cook, Rachel Marine, Mili Sheth, Jasmine

		Centers for Disease Control and Prevention	Padilla, Sarah Nobles, Mark Burroughs, Lori Rowe, Haibin Wang, Ben L. Rambo-Martin, Dhwani Batra, Justin Lee, Suxiang Tong
EPI_ISL_1094373	Philadelphia Department of Public Health	Respiratory Viruses Branch, Division of Viral Diseases, Centers for Disease Control and Prevention	Krista Queen, Yan Li, Ying Tao, Jing Zhang, Anna Uehara, Anna Montmayeur, Clinton R. Paden, Peter W. Cook, Rachel Marine, Mili Sheth, Jasmine Padilla, Sarah Nobles, Mark Burroughs, Lori Rowe, Haibin Wang, Ben L. Rambo-Martin, Dhwani Batra, Justin Lee, Suxiang Tong
EPI_ISL_1094375, EPI_ISL_1094376	DE Public Health Laboratory	Respiratory Viruses Branch, Division of Viral Diseases, Centers for Disease Control and Prevention	Krista Queen, Yan Li, Ying Tao, Jing Zhang, Anna Uehara, Anna Montmayeur, Clinton R. Paden, Peter W. Cook, Rachel Marine, Mili Sheth, Jasmine Padilla, Sarah Nobles, Mark Burroughs, Lori Rowe, Haibin Wang, Ben L. Rambo-Martin, Dhwani Batra, Justin Lee, Suxiang Tong
EPI_ISL_1094378	NYSDOH Wadsworth Center, Virology Lab	Respiratory Viruses Branch, Division of Viral Diseases, Centers for Disease Control and Prevention	Krista Queen, Yan Li, Ying Tao, Jing Zhang, Anna Uehara, Anna Montmayeur, Clinton R. Paden, Peter W. Cook, Rachel Marine, Mili Sheth, Jasmine Padilla, Sarah Nobles, Mark Burroughs, Lori Rowe, Haibin Wang, Ben L. Rambo-Martin, Dhwani Batra, Justin Lee, Suxiang Tong
EPI_ISL_1094379	PA Department of Health, Bureau of Laboratories	Respiratory Viruses Branch, Division of Viral Diseases, Centers for Disease Control and Prevention	Krista Queen, Yan Li, Ying Tao, Jing Zhang, Anna Uehara, Anna Montmayeur, Clinton R. Paden, Peter W. Cook, Rachel Marine, Mili Sheth, Jasmine Padilla, Sarah Nobles, Mark Burroughs, Lori Rowe, Haibin Wang, Ben L. Rambo-Martin, Dhwani Batra, Justin Lee, Suxiang Tong
EPI_ISL_1094381	VA-Division of Consolidated Laboratory Services	Respiratory Viruses Branch, Division of Viral Diseases, Centers for Disease Control and Prevention	Krista Queen, Yan Li, Ying Tao, Jing Zhang, Anna Uehara, Anna Montmayeur, Clinton R. Paden, Peter W. Cook, Rachel Marine, Mili Sheth, Jasmine Padilla, Sarah Nobles, Mark Burroughs, Lori Rowe, Haibin Wang, Ben L. Rambo-Martin, Dhwani Batra, Justin Lee, Suxiang Tong
EPI_ISL_1095411, EPI_ISL_1095413, EPI_ISL_1095414, EPI_ISL_1095415, EPI_ISL_1095416, EPI_ISL_1095423, EPI_ISL_1095424, EPI_ISL_1095427	Virginia Division of Consolidated Laboratory Services	Virginia Division of Consolidated Laboratory Services	Virginia DCLS
EPI_ISL_1097633	Colorado Department of Public Health and Environment	Colorado Department of Puplic Health and Environment	Laura Bankers, Molly C. Hetherington-Rauth, Diana Ir, Shannon Ely, Shannon R. Matzinger, Sarah Elizabeth Totten, Emily A. Travanty
EPI_ISL_1097970, EPI_ISL_1098377, EPI_ISL_1098451, EPI_ISL_1098457, EPI_ISL_1098597, EPI_ISL_1098598, EPI_ISL_1098600	Pandemic Response Lab - NYC	Pandemic Response Lab, R&D	Henry Lee, Michael Hammerling, Melissa Hopkins, Cybill del Castillo, Shinyoung Clair Kang, William Ward, Pradeep Bugga, Haiping Hao, Jon Laurent
EPI_ISL_1098852	Jessa	Jessa	Cruys et al. on behalf of the Jessa_cmdLab
EPI_ISL_1111519, EPI_ISL_1111551, EPI_ISL_1111552, EPI_ISL_1111553, EPI_ISL_1111554, EPI_ISL_1111555, EPI_ISL_1111556, EPI_ISL_1111557, EPI_ISL_1111558, EPI_ISL_1111559, EPI_ISL_1111560, EPI_ISL_1111561, EPI_ISL_1111562, EPI_ISL_1111563			
see above	Virginia Division of Consolidated Laboratory Services	Virginia Division of Consolidated Laboratory Services	Virginia DCLS
EPI_ISL_1113164	NJ Rapid Response Teams	New Jersey Public Health and Environmental Laboratories (PHEL)	Lindsey Bodnar, Shiv Verma, Dana Woell, Byeong Jeong
EPI_ISL_1115007	Lighthouse Lab in Milton Keynes	Wellcome Sanger Institute for the COVID-19 Genomics UK (COG-UK) Consortium	The Lighthouse Lab in Milton Keynes and Alex Alderton, Roberto Amato, Jeffrey Barrett, Sonia Goncalves, Ewan Harrison, David K. Jackson, Ian Johnston, Dominic Kwiatkowski, Cordelia Langford, John Sillitoe on behalf of the Wellcome Sanger Institute COVID-19 Surveillance Team
EPI_ISL_1116533	NYU Langone Health	Departments of Pathology and Medicine, New York University School of Medicine	Adriana Heguy, Dacia Dimartino, Emily Guzman, Christian Marier, Peter Meyn, Sitharam Ramaswami, Gael Westby, Paul Zappile, Yutong Zhang, Paolo Cotzia, Guiqing Wang
EPI_ISL_1117776, EPI_ISL_1117796, EPI_ISL_1118029, EPI_ISL_1118030, EPI_ISL_1118031, EPI_ISL_1118032, EPI_ISL_1118033, EPI_ISL_1118034, EPI_ISL_1118035, EPI_ISL_1118036, EPI_ISL_1118037, EPI_ISL_1118038, EPI_ISL_1118039, EPI_ISL_1118040, EPI_ISL_1118041, EPI_ISL_1118042, EPI_ISL_1118043, EPI_ISL_1118044, EPI_ISL_1118045, EPI_ISL_1118046, EPI_ISL_1118047, EPI_ISL_1118048, EPI_ISL_1118049, EPI_ISL_1118079, EPI_ISL_1118080			
see above	Austrian Agency for Health and Food Safety (AGES)	Bergthaler laboratory, CeMM Research Center for Molecular Medicine of the Austrian Academy of Sciences	Lukas Endler, Anna Schedl, Thomas Penz, Benedikt Agerer, Maelle Le Moing, Michael Schuster, Bekir Erguner, Jan Laine, Martin Senekowitsch, Christoph Bock, Andreas Bergthaler
EPI_ISL_1121031	Area of Virology, Serology and Virology Division (SAViD), New South Wales Health Pathology Randwick	Virology Research Laboratory; Area of Virology, Serology and Virology Division (SAViD), New South Wales Health Pathology Randwick	Foster, C.; Au, J.; Ruiz Silva, M.; Deveson, I.; Bull, R.; Van Hal, S.; Rawlinson, W.
EPI_ISL_1121234, EPI_ISL_1121240, EPI_ISL_1121262, EPI_ISL_1121263	Maryland Public Health Laboratory	Maryland Public Health Laboratory	Maryland Department of Health Laboratories Administration
EPI_ISL_1121362	NJ Rapid Response Teams	New Jersey Public Health and Environmental Laboratories (NJ PHEL)	Lindsey Bodnar, Shiv K. Verma, Dana Woell, Byeong Jeong
EPI_ISL_1121934	Department of Health & Mental Hygiene Corona	New York City Public Health Laboratory	Jade Wang, et al.
EPI_ISL_1123448	SARS-CoV-2 testing team, National Institute of Infectious Diseases	Pathogen Genomics Center, National Institute of Infectious Diseases	Tsuyoshi Sekizuka, Kentaro Itokawa, Rina Tanaka, Masanori Hashino, Yoshihiro Kaku, Yasutaka Hoshino, Chikako Shimokawa, Eunsil Park, Tsuguto Fujimoto, Makoto Kuroda
EPI_ISL_1123466	Pathogen Genomics Center, National Institute of Infectious Diseases	Pathogen Genomics Center, National Institute of Infectious Diseases	Tsuyoshi Sekizuka, Kentaro Itokawa, Rina Tanaka, Masanori Hashino, Makoto Kuroda
EPI_ISL_1123467	Gunma Prefectural Institute of Public Health and Environmental Sciences	Pathogen Genomics Center, National Institute of Infectious Diseases	Tsuyoshi Sekizuka, Kentaro Itokawa, Rina Tanaka, Masanori Hashino, Makoto Kuroda
EPI_ISL_1123468	SARS-CoV-2 testing team, National Institute of Infectious Diseases	Pathogen Genomics Center, National Institute of Infectious Diseases	Tsuyoshi Sekizuka, Kentaro Itokawa, Rina Tanaka, Masanori Hashino, Shigeru Tajima, Takahiro Maeki, Eri Nakayama , Motohiko Ogawa , Chang-Kweng Lim, Makoto Kuroda
EPI_ISL_1123469, EPI_ISL_1123470, EPI_ISL_1123471	SARS-CoV-2 testing team, National Institute of Infectious Diseases	Pathogen Genomics Center, National Institute of Infectious Diseases	Tsuyoshi Sekizuka, Kentaro Itokawa, Rina Tanaka, Masanori Hashino, Yoshihiro Kaku, Yasutaka Hoshino, Chikako Shimokawa, Eunsil Park, Tsuguto Fujimoto, Makoto Kuroda
EPI_ISL_1123472, EPI_ISL_1123473, EPI_ISL_1123474, EPI_ISL_1123475, EPI_ISL_1123476, EPI_ISL_1123477, EPI_ISL_1123478	Niigata Prefectural Institute of Public Health and Environmental Sciences	Pathogen Genomics Center, National Institute of Infectious Diseases	Tsuyoshi Sekizuka, Kentaro Itokawa, Rina Tanaka, Masanori Hashino, Makoto Kuroda
EPI_ISL_1123479, EPI_ISL_1123480, EPI_ISL_1123481, EPI_ISL_1123482	Pathogen Genomics Center, National Institute of Infectious Diseases	Pathogen Genomics Center, National Institute of Infectious Diseases	Tsuyoshi Sekizuka, Kentaro Itokawa, Rina Tanaka, Masanori Hashino, Makoto Kuroda
EPI_ISL_1127105	Tokyo Metropolitan Institute of Public Health	Tokyo Metropolitan Institute of Public Health	Masaki Hayashi, Takako Yamazaki, Mami Ohgai, Maaya Isono, Eri Satou, Kana Kimoto, Hiroshi Hayashi, Ayano Hotta, Mami Nagashima,Takushi Fujiwara, Takashi Chiba, Kenji Sadamasu
EPI_ISL_1127158, EPI_ISL_1127159, EPI_ISL_1127160, EPI_ISL_1127161, EPI_ISL_1127162	Pathogen Genomics Center, National Institute of Infectious Diseases	Pathogen Genomics Center, National Institute of Infectious Diseases	Tsuyoshi Sekizuka, Kentaro Itokawa, Rina Tanaka, Masanori Hashino, Makoto Kuroda
EPI_ISL_1127164, EPI_ISL_1127165, EPI_ISL_1127166, EPI_ISL_1127167, EPI_ISL_1127168, EPI_ISL_1127169, EPI_ISL_1127170, EPI_ISL_1127171, EPI_ISL_1127172, EPI_ISL_1127173, EPI_ISL_1127174, EPI_ISL_1127175, EPI_ISL_1127176, EPI_ISL_1127177, EPI_ISL_1127178, EPI_ISL_1127179, EPI_ISL_1127180, EPI_ISL_1127181, EPI_ISL_1127182, EPI_ISL_1127183			
see above	Chiba Prefectural Institute of Public Health	Pathogen Genomics Center, National Institute of Infectious Diseases	Tsuyoshi Sekizuka, Kentaro Itokawa, Rina Tanaka, Masanori Hashino, Makoto Kuroda
EPI_ISL_1127184, EPI_ISL_1127185	Pathogen Genomics Center, National Institute of Infectious Diseases	Pathogen Genomics Center, National Institute of Infectious Diseases	Tsuyoshi Sekizuka, Kentaro Itokawa, Rina Tanaka, Masanori Hashino, Makoto Kuroda
EPI_ISL_1128175	SARS-CoV-2 testing team, National Institute of Infectious Diseases	Pathogen Genomics Center, National Institute of Infectious Diseases	Tsuyoshi Sekizuka, Kentaro Itokawa, Rina Tanaka, Masanori Hashino, Chang-Kweng Lim, Takahiro Maeki, Motohiko Ogawa , Eri Nakayama , Shigeru Tanjima, Makoto Kuroda
EPI_ISL_1128177, EPI_ISL_1128178,	SARS-CoV-2 testing team, National Institute of Infectious	Pathogen Genomics Center, National Institute of Infectious	Tsuyoshi Sekizuka, Kentaro Itokawa, Rina Tanaka, Masanori Hashino, Yoshihiro Kaku, Yasutaka Hoshino, Chikako Shimokawa, Kyoko Saito, Tsuguto

EPI_ISL_1128179	Diseases	Diseases	Fujimoto, Makoto Kuroda
EPI_ISL_1129230, EPI_ISL_1129231, EPI_ISL_1129235, EPI_ISL_1129236	Tokyo Metropolitan Institute of Public Health	Tokyo Metropolitan Institute of Public Health	Masaki Hayashi, Takako Yamazaki, Mami Ohgai, Maaya Isono, Eri Satou, Kana Kimoto, Hiroshi Hayashi, Ayano Hotta, Mami Nagashima,Takushi Fujiwara, Takashi Chiba, Kenji Sadamasu
EPI_ISL_1131161, EPI_ISL_1131162, EPI_ISL_1131163	SARS-CoV-2 testing team, National Institute of Infectious Diseases	Pathogen Genomics Center, National Institute of Infectious Diseases	Tsuyoshi Sekizuka, Kentaro Itokawa, Rina Tanaka, Masanori Hashino, Kento Fukano, Kousho Wakae, Hussein H Aly, Takanobu Kato, Makoto Kuroda
EPI_ISL_1131164, EPI_ISL_1131165, EPI_ISL_1131166, EPI_ISL_1131167, EPI_ISL_1131168, EPI_ISL_1131169, EPI_ISL_1131170, EPI_ISL_1131171, EPI_ISL_1131172, EPI_ISL_1131173	Niigata Prefectural Institute of Public Health and Environmental Sciences	Pathogen Genomics Center, National Institute of Infectious Diseases	Tsuyoshi Sekizuka, Kentaro Itokawa, Rina Tanaka, Masanori Hashino, Makoto Kuroda
EPI_ISL_1137009, EPI_ISL_1137010	Delaware Public Health Lab	Delaware Public Health Lab	Gregory Hovan
EPI_ISL_1137526	Clinical Molecular Microbiology Laboratory, UNC Hospitals	Jeremy Wang	Jeremy Wang, Alexander Rubinsteyn, Colleen Rice, Jason Smedberg, Shawn Hawken, Melissa Miller, Corbin Jones, Robert Hagan
EPI_ISL_1147173, EPI_ISL_1147174, EPI_ISL_1147176, EPI_ISL_1147177	MVZ Labor Krone GbR	Robert Koch Institute	unknown
EPI_ISL_1158309	Michigan Department of Health and Human Services, Bureau of Laboratories	Michigan Department of Health and Human Services, Bureau of Laboratories	Blankenship HM, Riner D, Soehnlen MK
EPI_ISL_1159108	DC Public Health Lab/ Dept. of Forensic Sciences	DC Public Health Lab/ Dept. of Forensic Sciences	Scott Nguyen, Janis Doss, Elizabeth Zelaya, Sarah Scott, Connie Maza, Monica Mann, Brittany Hamilton, David Payne, Jocelyn Hauser
EPI_ISL_1160112	Laboratory Corporation of America	Respiratory Viruses Branch, Division of Viral Diseases, Centers for Disease Control and Prevention	Peter W. Cook, Dakota Howard, Dhwani Batra, Ben L. Rambo-Martin, Minoo Agarwal, Eyad Almasri Debbie Boles, Ayla Burns, Nuthawin Charoensri, Oren Cohen, Susan Countryman, Mary Ann Cristobal, Bobbi Croy, Suzanne Dale, Hrushikesh Deshmukh, Amanda Douglas, Vincent Drouillon, Marcia Eisenberg, Howard Engler, Rama Ghatti, Prashant Gupta, Susan Hicks, Jake Humphrey, Lax Iyer, Manoj Jain, Mohan Kolli, Brian Krueger, Tim Kuphal, Stanley Letovsky, Michael Levandoski, Craig Lukasik, Jonathan Meltzer, Brian Norvell, Mindy Nye, Scott Parker, Christos Petropoulos, John Pruitt, Steven Ragan, Scott Ryan, Mike Sapeta, Jana Schroth, Suresh Babu Selvaraju, Goran Stevovic, Amanda Suchanek, Andrea Throop, Lyndon Tilson, Thomas Urban, Joe Voshell, Kimberly Wagner, Jonathan Williams, Mary Williamson, Qian Zeng, Tricia Zwiefelhofer, Clinton R. Paden, Suxiang Tong, Duncan MacCannell
EPI_ISL_1164927, EPI_ISL_1164928, EPI_ISL_1164929	Genome Analysis Center, Yamanashi Central Hospital	Genome Analysis Center, Yamanashi Central Hospital	Yosuke Hirotsu
EPI_ISL_1165567, EPI_ISL_1165647, EPI_ISL_1165649	Dutch COVID-19 response team	National Institute for Public Health and the Environment (RIVM)	Adam Meijer, Harry Vennema, Dirk Eggink, Jeroen Cremer, Sharon van den Brink, Bas van der Veer, AnneMarie van den Brandt, Florian Zwagemaker, Dennis Schmitz, Chantal Reusken, on behalf of the national COVID-19 response team
EPI_ISL_1165877	University of Liège COVID-19 testing center	GIGA Medical Genomics	Keith Durkin, Maria Artesi, Bouchra Boujemla, Nathalie Renotte, Cécile Meex, Sébastien Bontems, Fabrice Bureau, Laurent Gillet, Wouter Coppieters, Marie-Pierre Hayette, Vincent Bours
EPI_ISL_1167279, EPI_ISL_1167290	Maryland Public Health Laboratory	Maryland Public Health Laboratory	Maryland Department of Health Laboratories Administration
EPI_ISL_1170851, EPI_ISL_1170852	Public Health Ontario Laboratory	Public Health Ontario Laboratory	Vanessa G Allen, Philip Banh, Yao Chen, Richard de Borja, Alireza Eshaghi, Nahuel Fittipaldi, Christine Frantz, Jonathan B Gubbay, Jennifer L Guthrie, Lawrence Heisler, Esha Joshi, Michael Laszloffy, Aimin Li, Michael CY Li, Dean Maxwell, Sandeep Nagra, Samir N Patel, Jared Simpson, Karthikeyan Sivaraman, Ashleigh Sullivan, Yogi Sundaravadanam, Sarah Teatero, Andre Villegas, Matthew Watson, Sandra Zittermann
EPI_ISL_1171601	SYNLAB	GIGA Medical Genomics	Keith Durkin, Maria Artesi, Sébastien Bontems, Raphaël Boreux, Bouchra Boujemla, Nathalie Renotte, Cécile Meex, Pierrette Melin, Marie-Pierre Hayette, Vincent Bours
EPI_ISL_1171783, EPI_ISL_1171787, EPI_ISL_1171793, EPI_ISL_1171796	Johns Hopkins Hospital Department of Pathology	Johns Hopkins Hospital Department of Pathology	C. Paul Morris, Chun Huai Luo, Adannaya Amadi, Matthew Schwartz, Nicholas Gallagher, Heba H. Mostafa
EPI_ISL_1172210, EPI_ISL_1172215, EPI_ISL_1172275, EPI_ISL_1172292, EPI_ISL_1172337, EPI_ISL_1172459, EPI_ISL_1172487, EPI_ISL_1172583, EPI_ISL_1172658, EPI_ISL_1173028	Pandemic Response Lab - NYC	Pandemic Response Lab, R&D	Henry Lee, Michael Hammerling, Melissa Hopkins, Cybill del Castillo, Shinyoung Clair Kang, William Ward, Pradeep Bugga, Haiping Hao, Jon Laurent
EPI_ISL_1180781, EPI_ISL_1180785, EPI_ISL_1180801, EPI_ISL_1180805, EPI_ISL_1180830	Austrian Agency for Health and Food Safety (AGES)	Bergthaler laboratory, CeMM Research Center for Molecular Medicine of the Austrian Academy of Sciences	Lukas Endler, Anna Schedl, Fabian Amman, Thomas Penz, Benedikt Agerer, Maelle Le Moing, Michael Schuster, Bekir Erguner, Jan Laine, Martin Senekowitsch, Christoph Bock, Andreas Bergthaler
EPI_ISL_1181642	DPHL	Delaware Public Health Lab	Gregory Hovan
EPI_ISL_1181985, EPI_ISL_1181994, EPI_ISL_1181996, EPI_ISL_1181998, EPI_ISL_1182000	Maryland Public Health Laboratory	Maryland Public Health Laboratory	Maryland Department of Health Laboratories Administration
EPI_ISL_1182090	Murphy Medical Association	Grubaugh Lab - Yale School of Public Health	Joseph Fauver, Mallery Breban, Isabell Ott, Tara Alpert, Mary Petrone, Anderson Brito, Chantal Vogels, Annie Watkins, Chaney Kalinich, Caleb Neal, Eva Laszlo, Steven Murphy, Nathan Grubaugh
EPI_ISL_1191140	Center for Virology	Center for Virology	Jeremy V. Camp, Irene Goerzer, Monika Redlberger-Fritz, Stephan W. Aberle
EPI_ISL_1195765, EPI_ISL_1195777, EPI_ISL_1195797	NYU Langone Health	Departments of Pathology and Medicine, New York University School of Medicine	Adriana Heguy, Dacia Dimartino, Emily Guzman, Christian Marier, Peter Meyn, Sitharam Ramaswami, Gael Westby, Paul Zappile, Yutong Zhang, Paolo Cotzia, Guiqing Wang
EPI_ISL_1199159, EPI_ISL_1200053	Swedish national genomic surveillance program of SARS-CoV-2	The Public Health Agency of Sweden	Swedish national genomic surveillance program of SARS-CoV-2
EPI_ISL_1200525	DOHMH Riverside	New York City Public Health Laboratory	Jade Wang, et al.
EPI_ISL_1200528, EPI_ISL_1200529, EPI_ISL_1200530	DOHMH Chelsea	New York City Public Health Laboratory	Jade Wang, et al.
EPI_ISL_1200611	DOHMH Riverside	New York City Public Health Laboratory	Jade Wang, et al.
EPI_ISL_1209278	Department of Laboratory Medicine, Division of Clinical Virology, University of Medicine, Vienna	Bergthaler laboratory, CeMM Research Center for Molecular Medicine of the Austrian Academy of Sciences	Lukas Endler, Anna Schedl, Fabian Amman, Thomas Penz, Benedikt Agerer, Maelle Le Moing, Michael Schuster, Bekir Erguner, Jan Laine, Martin Senekowitsch, Christoph Bock, Andreas Bergthaler
EPI_ISL_1210498	Laboratorio Genzano - ASL RM 7	INMI Lazzaro Spallanzani IRCCS	CEM Gruber, B Bartolini, E Giombini, F Messina, M Rueca, O Butera, G Tramini, E Conti, MR Capobianchi, A Di Caro
EPI_ISL_1211598	Labor Dr. Wisplinghoff - Köln	Robert Koch Institute	unknown
EPI_ISL_1214341	SYNLAB MVZ Weiden	Robert Koch Institute	unknown
EPI_ISL_1215936	Labor Dr. Wisplinghoff - Köln	Robert Koch Institute	unknown
EPI_ISL_1224774	M Health Fairview	Minnesota Department of Health, Public Health Laboratory	Alexandra Lorentz, Jacob Garfin, Matt Plumb, and Xiong Wang
EPI_ISL_1224937	Houston Health Dept.	Houston Health Dept.	Ryker Penn, Pamela Brown, Adolpho Lara
EPI_ISL_1225731	Yale Clinical Virology Lab	Grubaugh Lab - Yale School of Public Health	Joseph Fauver, Mallery Breban, Isabell Ott, Tara Alpert, Mary Petrone, Anderson Brito, Chantal Vogels, Annie Watkins, Chaney Kalinich, Marie L. Landry,

			Nathan Grubaugh
EPI_ISL_1226457	MONTEFIORE MEDICAL CENTER LABORATORIES	Wadsworth Center, New York State Department of Health	Kirsten St. George, Daryl M. Lamson, Alexis Russel, Matthew Shudt, Melissa A Leisner, Jonathan Plitnick, Navjot Singh, John Kelly, Erasmus Schneider, Erica Lasek-Nesselquist
EPI_ISL_1227232, EPI_ISL_1227233	ALBANY MEDICAL CENTER	Wadsworth Center, New York State Department of Health	Kirsten St. George, Daryl M. Lamson, Alexis Russel, Matthew Shudt, Melissa A Leisner, Jonathan Plitnick, Navjot Singh, John Kelly, Erasmus Schneider, Erica Lasek-Nesselquist
EPI_ISL_1227234	Wadsworth Center, New York State Department of Health	Wadsworth Center, New York State Department of Health	Kirsten St. George, Daryl M. Lamson, Alexis Russel, Matthew Shudt, Melissa A Leisner, Jonathan Plitnick, Navjot Singh, John Kelly, Erasmus Schneider, Erica Lasek-Nesselquist
EPI_ISL_1227235	ALBANY MEDICAL CENTER	Wadsworth Center, New York State Department of Health	Kirsten St. George, Daryl M. Lamson, Alexis Russel, Matthew Shudt, Melissa A Leisner, Jonathan Plitnick, Navjot Singh, John Kelly, Erasmus Schneider, Erica Lasek-Nesselquist
EPI_ISL_1227237	Columbia University Irving Medical Center	Wadsworth Center, New York State Department of Health	Kirsten St. George, Daryl M. Lamson, Alexis Russel, Matthew Shudt, Melissa A Leisner, Jonathan Plitnick, Navjot Singh, John Kelly, Erasmus Schneider, Erica Lasek-Nesselquist
EPI_ISL_1227239, EPI_ISL_1227240, EPI_ISL_1227241	SUNY UPSTATE MEDICAL UNIVERSITY	Wadsworth Center, New York State Department of Health	Kirsten St. George, Daryl M. Lamson, Alexis Russel, Matthew Shudt, Melissa A Leisner, Jonathan Plitnick, Navjot Singh, John Kelly, Erasmus Schneider, Erica Lasek-Nesselquist
EPI_ISL_1227242	MEMORIAL SLOAN KETTERING CANCER CENTER	Wadsworth Center, New York State Department of Health	Kirsten St. George, Daryl M. Lamson, Alexis Russel, Matthew Shudt, Melissa A Leisner, Jonathan Plitnick, Navjot Singh, John Kelly, Erasmus Schneider, Erica Lasek-Nesselquist
EPI_ISL_1227244	NYC Pandemic Response Lab	Wadsworth Center, New York State Department of Health	Kirsten St. George, Daryl M. Lamson, Alexis Russel, Matthew Shudt, Melissa A Leisner, Jonathan Plitnick, Navjot Singh, John Kelly, Erasmus Schneider, Erica Lasek-Nesselquist
EPI_ISL_1227245	SARATOGA HOSPITAL LABORATORY	Wadsworth Center, New York State Department of Health	Kirsten St. George, Daryl M. Lamson, Alexis Russel, Matthew Shudt, Melissa A Leisner, Jonathan Plitnick, Navjot Singh, John Kelly, Erasmus Schneider, Erica Lasek-Nesselquist
EPI_ISL_1227246	Wadsworth Center, New York State Department of Health	Wadsworth Center, New York State Department of Health	Kirsten St. George, Daryl M. Lamson, Alexis Russel, Matthew Shudt, Melissa A Leisner, Jonathan Plitnick, Navjot Singh, John Kelly, Erasmus Schneider, Erica Lasek-Nesselquist
EPI_ISL_1227247, EPI_ISL_1227248	WESTCHESTER MEDICAL CENTER	Wadsworth Center, New York State Department of Health	Kirsten St. George, Daryl M. Lamson, Alexis Russel, Matthew Shudt, Melissa A Leisner, Jonathan Plitnick, Navjot Singh, John Kelly, Erasmus Schneider, Erica Lasek-Nesselquist
EPI_ISL_1229050	UNILIANS DECINES	CNR Virus des Infections Respiratoires - France SUD	Antonin Bal, Gregory Destras, Gwendolyne Burfin, Hadrien Regue, Quentin Semanas, Martine Valette, Bruno Lina, Laurence Josset
EPI_ISL_1231615	Fulgent Genetics	Fulgent Genetics	Harry Gao, Mickey Li, John Gao, Joseph Fierro, Benafsh Sapra, Becky Tsai, Yan Meng, Doreen Ng, James Xie
EPI_ISL_1232310, EPI_ISL_1232883, EPI_ISL_1232909	Dutch COVID-19 response team	National Institute for Public Health and the Environment (RIVM)	Adam Meijer, Harry Vennema, Dirk Eggink, Jeroen Cremer, Sharon van den Brink, Bas van der Veer, AnneMarie van den Brandt, Florian Zwagemaker, Dennis Schmitz, Chantal Reusken, on behalf of the national COVID-19 response team
EPI_ISL_1233286, EPI_ISL_1233289, EPI_ISL_1233290, EPI_ISL_1233296, EPI_ISL_1233297, EPI_ISL_1233298, EPI_ISL_1233299, EPI_ISL_1233302	Massachusetts State Public Health Laboratory	Massachusetts State Public Health Laboratory	Andrew Lang, Timelia Fink, Glen Gallagher, Sandra Smole
EPI_ISL_1234359	Colorado Department of Public Health and Environment	Colorado Department of Public Health and Environment	Laura Bankers, Molly C. Hetherington-Rauth, Diana Ir, Shannon Ely, Shannon R. Matzinger, Sarah Elizabeth Totten, Emily A. Travanty
EPI_ISL_1235668, EPI_ISL_1235669	INNO Diagnostics Reference Laboratory	RCMI-Center for Research Resources, Ponce Research Institute	Vanessa Rivera-Amill, Andrea Arias-García, Raphael Sánchez-Torres
EPI_ISL_1235902, EPI_ISL_1236250, EPI_ISL_1236275, EPI_ISL_1236398, EPI_ISL_1236456, EPI_ISL_1236698, EPI_ISL_1237071, EPI_ISL_1237355, EPI_ISL_1237578, EPI_ISL_1237614, EPI_ISL_1237646, EPI_ISL_1238043, EPI_ISL_1238055, EPI_ISL_1238056, EPI_ISL_1238143, EPI_ISL_1238513, EPI_ISL_1238521, EPI_ISL_1238560			
see above	Houston Methodist Hospital	Houston Methodist Hospital	S. Wesley Long, Randall J. Olsen, Paul A. Christensen, Sishir Subedi, Robert Olson, James J. Davis, Matthew Ojeda Saavedra, Prasanti Yerramilli, Layne Pruitt, Kristina Reppond, Madison N. Shyer, Jessica Cambric, Ilya J. Finkelstein, Jimmy Gollihar, and James M. Musser
EPI_ISL_1238798, EPI_ISL_1238813	MD PHL	MD PHL	Maryland Department of Health Laboratories Administration
EPI_ISL_1238913, EPI_ISL_1238917, EPI_ISL_1238951, EPI_ISL_1238992	Johns Hopkins Hospital Department of Pathology	Johns Hopkins Hospital Department of Pathology	C. Paul Morris, Chun Huai Luo, Adannaya Amadi, Matthew Schwartz, Nicholas Gallagher, Heba H. Mostafa
EPI_ISL_1239140	Ohio State University Wexner Medical Center	Polaris Molecular Laboratory	Ru P, Chappell D, Chang Y-S, Tu H, Snyder P, Pancholi P, Koenig S, Corcoran S, Jones D
EPI_ISL_1239141	Ohio State University Wexner Medical Center	Polaris Molecular Laboratory	Chappell D, Ru P, Chang Y-S, Tu H, Snyder P, Pancholi P, Koenig S, Corcoran S, Jones D
EPI_ISL_1239142	Ohio State University Wexner Medical Cente	Polaris Molecular Laboratory	Chang Y-S, Ru P, Chappell D, Tu H, Snyder P, Pancholi P, Koenig S, Corcoran S, Jones D
EPI_ISL_1240005	Florida Bureau of Public Health Laboratories	Florida Bureau of Public Health Laboratories	Sarah Schmedes, Jason Blanton
EPI_ISL_1240218	Jessa	Jessa	Cruys et al. on behalf of the Jessa_cmdLab
EPI_ISL_1240905	Clinical Molecular Microbiology Laboratory, UNC Hospitals	Jeremy Wang	Jeremy Wang, Alexander Rubinsteyn, Colleen Rice, Jason Smedberg, Shawn Hawken, Melissa Miller, Corbin Jones, Robert Hagan
EPI_ISL_1241740	SYNLAB	GIGA Medical Genomics	Keith Durkin, Maria Artesi, Sébastien Bontems, Raphaël Boreux, Bouchra Boujemla, Nathalie Renotte, Cécile Meex, Pierrette Melin, Marie-Pierre Hayette, Vincent Bours
EPI_ISL_1241750	University of Liège COVID-19 testing center	GIGA Medical Genomics	Keith Durkin, Maria Artesi, Sébastien Bontems, Raphaël Boreux, Bouchra Boujemla, Nathalie Renotte, Cécile Meex, Pierrette Melin, Marie-Pierre Hayette, Vincent Bours
EPI_ISL_1242142	Lighthouse Lab in Cambridge	Wellcome Sanger Institute for the COVID-19 Genomics UK (COG-UK) Consortium	Rob Howes, The Lighthouse Lab in Cambridge and Alex Alderton, Roberto Amato, Jeffrey Barrett, Sonia Goncalves, Ewan Harrison, David K. Jackson, Ian Johnston, Dominic Kwiatkowski, Cordelia Langford, John Sillitoe on behalf of the Wellcome Sanger Institute COVID-19 Surveillance Team
EPI_ISL_1250527	Michigan Department of Health and Human Services, Bureau of Laboratories	Michigan Department of Health and Human Services, Bureau of Laboratories	Blankenship HM, Riner D, Soehnlen MK
EPI_ISL_1252630	TXDSHS	TXDSHS	Rashmi Tuladhar, Bonnie Oh, Jenny Zhang, Maliha Rahman, Mayela Pedrueza, Anita Pokharel, Myong Koag, Chun Wang, Rachel Lee, Grace Kubin
EPI_ISL_1252844, EPI_ISL_1252845	INNO Diagnostics Reference Laboratory	RCMI-Center for Research Resources, Ponce Research Institute	Vanessa Rivera-Amill, Andrea Arias-García, Raphael Sánchez-Torres
EPI_ISL_1252858, EPI_ISL_1252882	University of Wisconsin-Madison AIDS Vaccine Research Laboratories	University of Wisconsin-Madison AIDS Vaccine Research Laboratories	Gage Moreno, Katarina Braun, et al. AIDS Vaccine Research Laboratories
EPI_ISL_1253727, EPI_ISL_1253807, EPI_ISL_1253834	Broad Institute Clinical Research Sequencing Platform	Infectious Disease Program, Broad Institute of Harvard and MIT	Lemieux,J.E., Siddle,K.J., Adams,G., Gladden-Young,A., Lagerborg,K., Rudy,M., DeRuff,K., Carter,A., Normandin,E., Bauer,M., Reilly,S., Tomkins-Tinch,C., Loreth,C., Chaluvadi,S., Birren,B.W., Gallagher,G., Smole,S., Park,D.J., MacInnis,B.L., and Sabeti,P.C.
EPI_ISL_1254264	NJDOH, Public Health and Environmental Laboratories	NJ_PHEL	Lindsey Bodnar, Shiv K. Verma, Dana Woell, Byeong Jeong
EPI_ISL_1258929, EPI_ISL_1258930, EPI_ISL_1258931, EPI_ISL_1258932, EPI_ISL_1258933	Pandemic Response Lab - NYC	Pandemic Response Lab, R&D	Henry Lee, Michael Hammerling, Melissa Hopkins, Cybill del Castillo, Shinyoung Clair Kang, William Ward, Pradeep Bugga, Haiping Hao, Jon Laurent
EPI_ISL_1261141	Molecular & Genomic Pathology Laboratory, Thomas Jefferson University Hospital	Molecular & Genomic Pathology Laboratory, Thomas Jefferson University Hospital	Nitika Badjatia, Run Jin, Zi-xuan Wang

EPI_ISL_1261855	DOHMH Chelsea	New York City Public Health Laboratory	Jade Wang, et al.
EPI_ISL_1261856	DOHMH Central Harlem	New York City Public Health Laboratory	Jade Wang, et al.
EPI_ISL_1262512, EPI_ISL_1262573, EPI_ISL_1262574	NYU Langone Health	Departments of Pathology and Medicine, New York University School of Medicine	Adriana Heguy, Dacia Dimartino, Emily Guzman, Christian Marier, Peter Meyn, Sitharam Ramaswami, Gael Westby, Paul Zappile, Yutong Zhang, Paolo Cotzia, Guiqing Wang
EPI_ISL_1265682, EPI_ISL_1265759	CNR Virus des Infections Respiratoires - France SUD	CNR Virus des Infections Respiratoires - France SUD	Antonin Bal, Gregory Destras, Gwendolyne Burfin, Hadrien Regue, Quentin Semanas, Martine Valette, Bruno Lina, Laurence Josset
EPI_ISL_1268202	DPHL	Delaware Public Health Lab	Rebecca Savage
EPI_ISL_1272197	MD PHL	MD PHL	Maryland Department of Health Laboratories Administration
EPI_ISL_1273320, EPI_ISL_1273321, EPI_ISL_1273324, EPI_ISL_1273346, EPI_ISL_1273356	The Jackson Laboratory	The Jackson Laboratory	Bergeron D, Renzette N, Adams M, Omerza G, Kelly K, Li L
EPI_ISL_1275651	Lighthouse Lab in Cambridge	Wellcome Sanger Institute for the COVID-19 Genomics UK (COG-UK) Consortium	Rob Howes, The Lighthouse Lab in Cambridge and Alex Alderton, Roberto Amato, Jeffrey Barrett, Sonia Goncalves, Ewan Harrison, David K. Jackson, Ian Johnston, Dominic Kwiatkowski, Cordelia Langford, John Sillitoe on behalf of the Wellcome Sanger Institute COVID-19 Surveillance Team
EPI_ISL_1279222, EPI_ISL_1279238	Microvida	Microvida	J. Stohr, S.D. Pas, J. Verweij
EPI_ISL_1282555	Labor Dr. Wisplinghoff - Köln	Robert Koch Institute	unknown
EPI_ISL_1284425	SYNLAB MVZ Weiden	Robert Koch Institute	unknown
EPI_ISL_1292684	State Laboratories Division, Hawaii State Department of Health	State Laboratories Division, Hawaii State Department of Health	Pamela O'Brien, Drew Kuwazaki, Ayana Garnet, Razvan Sultana, Edward Desmond
EPI_ISL_1292822, EPI_ISL_1292833, EPI_ISL_1292834, EPI_ISL_1292836, EPI_ISL_1292851, EPI_ISL_1292854, EPI_ISL_1292874, EPI_ISL_1292896, EPI_ISL_1292899, EPI_ISL_1292901, EPI_ISL_1292903, EPI_ISL_1292930, EPI_ISL_1292932, EPI_ISL_1292938, EPI_ISL_1292940			
see above	Maryland Genomics, Institute for Genome Sciences, University of Maryland School of Medicine	Maryland Genomics, Institute for Genome Sciences, University of Maryland School of Medicine	Tallon, Luke J; Sadzewicz, Lisa D; Humphrys, Mike; Ott, Sandra; Roussey, Holly; Mehta, Aditya; Vavikolanu, Kranthi; Fraser, Claire M; Ravel, Jacques
EPI_ISL_1292982	Yale Clinical Virology Lab	Grubaugh Lab - Yale School of Public Health	Joseph Fauver, Mallery Breban, Isabell Ott, Tara Alpert, Mary Petrone, Anderson Brito, Chantal Vogels, Annie Watkins, Chaney Kalinich, Jessica Rothman, Marie L. Landry, Nathan Grubaugh
EPI_ISL_1293041	MD Public Health Laboratory	MD Public Health Laboratory	Maryland Department of Health Laboratories Administration
EPI_ISL_1293234	Yale Clinical Virology Lab	Grubaugh Lab - Yale School of Public Health	Joseph Fauver, Mallery Breban, Isabell Ott, Tara Alpert, Mary Petrone, Anderson Brito, Chantal Vogels, Annie Watkins, Chaney Kalinich, Jessica Rothman, Marie L. Landry, Nathan Grubaugh
EPI_ISL_1296790, EPI_ISL_1296933, EPI_ISL_1297070	Sonora Quest Laboratories	TGen North	"Jolene Bowers, Heather Centner, Chris French, Hayley Yaglom, Ashlyn Pfeiffer, Darrin Lemmer, Dave Engelthaler, The Arizona COVID Genomics Union (ACGU)"
EPI_ISL_1300931, EPI_ISL_1300932, EPI_ISL_1300934, EPI_ISL_1300935	MSHS Clinical Microbiology Laboratories	MSHS Pathogen Surveillance Program	Ana S. Gonzalez-Reiche, Hala Alshammary, Mitchell J. Sullivan, Brianne Ciferri, Ajay Obla, Angela Amoako, Mahmoud Awawda, Daniel Floda, Julia Matthews, Ashley Salimbangon, Levy Sominsky, Katherine Beach, Kayla Russo, Charles Gleason, Shelcie Fabre, Giulio Kleiner, Zenab Khan, Bremy Alburquerque, Adriana van de Guchte, Komal Srivastava, Matthew M. Hernandez, Jayeeta Dutta, Denise Jurczyszak, Nancy Francoeur, Betsaida Salom Melo, Irina Oussenko, Gintaras Deikus, Juan Soto, Shwetha Hara Sridhar, Ying-Chih Wang, Kathryn Twyman, Deena R. Altman, Robert Sebra, Adolfo García-Sastre, Marta Luksza, Gopi Patel, Sarah Schaefer, Melissa Gitman, Michael D. Nowak, Alberto Paniz-Mondolfi, Emilia Mia Sordillo, Viviana Simon, Harm van Bakel
EPI_ISL_1303159, EPI_ISL_1303210	Microbiology Division, South Carolina Department of Health and Environmental Control	Microbiology Division, South Carolina Department of Health and Environmental Control	Flores,H., Freeman,J.
EPI_ISL_1303644, EPI_ISL_1303770, EPI_ISL_1303873, EPI_ISL_1303876, EPI_ISL_1304402, EPI_ISL_1305292, EPI_ISL_1305372, EPI_ISL_1305636	Houston Methodist Hospital	Houston Methodist Hospital	S. Wesley Long, Randall J. Olsen, Paul A. Christensen, Sishir Subedi, Robert Olson, James J. Davis, Matthew Ojeda Saavedra, Prasanti Yerramilli, Layne Pruitt, Kristina Reppond, Madison N. Shyer, Jessica Cambric, Ilya J. Finkelstein, Jimmy Gollihar, and James M. Musser
EPI_ISL_1305838, EPI_ISL_1305905, EPI_ISL_1305932, EPI_ISL_1305933, EPI_ISL_1305934, EPI_ISL_1305935, EPI_ISL_1305936, EPI_ISL_1305937	Johns Hopkins Hospital Department of Pathology	Johns Hopkins Hospital Department of Pathology	C. Paul Morris, Chun Huai Luo, Adannaya Amadi, Matthew Schwartz, Heba H. Mostafa
EPI_ISL_1306025	University Hospital for Infectious Diseases	Croatian Institute of Public Health	Irena Tabain, Ivana Ferenak
EPI_ISL_1306288, EPI_ISL_1306318, EPI_ISL_1306574, EPI_ISL_1306690, EPI_ISL_1307212	Pandemic Response Lab - NYC	Pandemic Response Lab, R&D	Henry Lee, Michael Hammerling, Melissa Hopkins, Cybill del Castillo, Shinyoung Clair Kang, William Ward, Pradeep Bugga, Sol Rey, Dylan Law, Haiping Hao, Jon Laurent
EPI_ISL_1312344, EPI_ISL_1312345	Gravity Diagnostics	Kentucky State Public Health Lab	Stephanie Lunn, Karim George, Joshua Tobias, William Grooms, Vaneet Arora, Matthew Johnson, Rachel Zinner, Rhonda Lucas
EPI_ISL_1312346, EPI_ISL_1312347	Kentucky State Public Health Lab	Kentucky State Public Health Lab	Stephanie Lunn, Karim George, Joshua Tobias, William Grooms, Vaneet Arora, Matthew Johnson, Rachel Zinner, Rhonda Lucas
EPI_ISL_1312355, EPI_ISL_1312359, EPI_ISL_1312360	Gravity Diagnostics	Kentucky State Public Health Lab	Stephanie Lunn, Karim George, Joshua Tobias, William Grooms, Vaneet Arora, Matthew Johnson, Rachel Zinner, Rhonda Lucas
EPI_ISL_1312795	Molecular & Genomic Pathology Laboratory, Thomas Jefferson University Hospital	Molecular & Genomic Pathology Laboratory, Thomas Jefferson University Hospital	Run Jin, Nitika Badjatia, Zi-xuan Wang
EPI_ISL_1313423, EPI_ISL_1313458, EPI_ISL_1313509, EPI_ISL_1313510	Karolinska University Hospital	Karolinska University Hospital	Jan Albert, Tobias Allander, Annelie Bjerkner, Sandra Broddesson, Robert Dyrdak, Martin Ekman, Lynda Eneh, Lina Guerra Blomqvist, Karolina Ininbergs, Tanja Normark, Isak Sylvin, Zhibing Yun, Martina Wahlund, Valtteri Wirta
EPI_ISL_1314020	CENTRE HOSPITALIER PIERRE OUDOT	CNR Virus des Infections Respiratoires - France SUD	Antonin Bal, Gregory Destras, Gwendolyne Burfin, Hadrien Regue, Quentin Semanas, Martine Valette, Bruno Lina, Laurence Josset
EPI_ISL_1314170, EPI_ISL_1314171, EPI_ISL_1314172, EPI_ISL_1314173, EPI_ISL_1314174, EPI_ISL_1314175, EPI_ISL_1314176, EPI_ISL_1314177	Kentucky State Public Health Lab	Kentucky State Public Health Lab	Stephanie Lunn, Karim George, Joshua Tobias, William Grooms, Vaneet Arora, Matthew Johnson, Rachel Zinner, Rhonda Lucas
EPI_ISL_1314178, EPI_ISL_1314179, EPI_ISL_1314180, EPI_ISL_1314181, EPI_ISL_1314182, EPI_ISL_1314183, EPI_ISL_1314184, EPI_ISL_1314185, EPI_ISL_1314187, EPI_ISL_1314198	Gravity Diagnostics	Kentucky State Public Health Lab	Stephanie Lunn, Karim George, Joshua Tobias, William Grooms, Vaneet Arora, Matthew Johnson, Rachel Zinner, Rhonda Lucas
EPI_ISL_1315137, EPI_ISL_1315171, EPI_ISL_1315220	The Jackson Laboratory	The Jackson Laboratory	Bergeron D, Renzette N, Adams M, Omerza G, Kelly K, Li L
EPI_ISL_1315310	Waikato Hospital	Institute of Environmental Science and Research (ESR)	Rachel Boyle, SallyAnn Harbison, Olivia Stroeven, Xiaoyun Ren, Matt Storey, Nikki Freed, Muhammad Faisal, Jing Wang, Hermes Perez, Anja Werno, Antje van der Linden, Arlo Upton, Chris Mansell, David Hammer, Dragana Drinkovic, Gary McAuliffe, Hana Sofia Andersson, James Ussher, Jill Sherwood,

			Josh Freeman, Julia Howard, Juliet Elvy, Mary DeAlmeida, Matt Blakiston, Matthew Rogers, Max Bloomfield, Michael Addidle, Michelle Balm, Sally Roberts, Sarah Jefferies, Sharmini Muttaiyah, Susan Morpeth, Susan Taylor, Timothy Blackmore, Vani Sathyendran, Veronica Playle, Virginia Hope, Erasmus Smit, Lauren Jelly, Olin Silander, Joep de Ligt
EPI_ISL_1318195	Department of Clinical Microbiology	GIGA Medical Genomics	Keith Durkin, Maria Artesi, Sébastien Bontems, Raphaël Boreux, Bouchra Boujemla, Nathalie Renotte, Cécile Meex, Pierrette Melin, Marie-Pierre Hayette, Vincent Bours
EPI_ISL_1318201	University of Liège COVID-19 testing center	GIGA Medical Genomics	Keith Durkin, Maria Artesi, Sébastien Bontems, Raphaël Boreux, Bouchra Boujemla, Nathalie Renotte, Cécile Meex, Pierrette Melin, Marie-Pierre Hayette, Vincent Bours
EPI_ISL_1318247	TN Division of Laboratory Services	Genomics and Discovery, Respiratory Viruses Branch, Division of Viral Diseases, Centers for Disease Control and Prevention	Ying Tao, Jing Zhang, Yan Li, Brian Lynch, Anna Kelleher, Krista Queen, Anna Uehara, Peter Cook, Han Jia Justin Ng, Clinton R. Paden, Haibin Wang, Suxiang Tong
EPI_ISL_1324362, EPI_ISL_1324363	LSUHS Emerging Viral Threat Laboratory	Microbial Genome Sequencing Center	Jeremy P. Kamil, Jennifer L. Carroll, Rona S. Scott, Maarten Van Diest, Andrew D. Yurochko, Christopher G. Kevil, Martin J. Sapp, Daniel J. Snyder, Vaughn S. Cooper, John A. Vanchiere
EPI_ISL_1324384, EPI_ISL_1324466	Genome Analysis Center, Kamma Memorial Hospital	Genome Analysis Center, Kamma Memorial Hospital	Hanako Yazawa, Jun Ishii, Satoko Soma, Kaori Watanabe, Takuya Yazawa, Michiaki Masuda, Hiroshi Kamma
EPI_ISL_1326070	Lighthouse Lab in Milton Keynes	Wellcome Sanger Institute for the COVID-19 Genomics UK (COG-UK) Consortium	The Lighthouse Lab in Milton Keynes and Alex Alderton, Roberto Amato, Jeffrey Barrett, Sonia Goncalves, Ewan Harrison, David K. Jackson, Ian Johnston, Dominic Kwiatkowski, Cordelia Langford, John Sillitoe on behalf of the Wellcome Sanger Institute COVID-19 Surveillance Team
EPI_ISL_1327391, EPI_ISL_1327460, EPI_ISL_1327507, EPI_ISL_1329295	Lighthouse Lab in Cambridge	Wellcome Sanger Institute for the COVID-19 Genomics UK (COG-UK) Consortium	Rob Howes, The Lighthouse Lab in Cambridge and Alex Alderton, Roberto Amato, Jeffrey Barrett, Sonia Goncalves, Ewan Harrison, David K. Jackson, Ian Johnston, Dominic Kwiatkowski, Cordelia Langford, John Sillitoe on behalf of the Wellcome Sanger Institute COVID-19 Surveillance Team
EPI_ISL_1335012	Public Health Ontario Laboratory	Public Health Ontario Laboratory	Vanessa G Allen, Philip Banh, Yao Chen, Richard de Borja, Alireza Eshaghi, Nahuel Fittipaldi, Christine Frantz, Jonathan B Gubbay, Jennifer L Guthrie, Lawrence Heisler, Esha Joshi, Michael Laszloffy, Aimin Li, Michael CY Li, Dean Maxwell, Sandeep Nagra, Samir N Patel, Jared Simpson, Karthikeyan Sivaraman, Ashleigh Sullivan, Yogi Sundaravadanam, Sarah Teatero, Andre Villegas, Matthew Watson, Sandra Zittermann
EPI_ISL_1336326	MD PHL	MD PHL	Maryland Department of Health Laboratories Administration
EPI_ISL_1337484	DOHMH Corona	New York City Public Health Laboratory	Jade Wang, et al.
EPI_ISL_1337926	Dutch COVID-19 response team	Medical Microbiology, Maastricht University Medical Centre	Jozef Dingemans*, Brian van der Veer*, Erik Beuken, Carmen Reumkens, Lieke van Alphen, Christian Hoebe, Paul Savelkoul
EPI_ISL_1340033	Houston Health Dept.	Houston Health Dept.	Ryker Penn, Pamela Brown, Adolpho Lara, Yanlai Lai
EPI_ISL_1340835	Northwestern Memorial Hospital	Northwestern University - Ozer Lab	Ramon Lorenzo-Redondo, Lacy M. Simons, Taylor J. Dean, Chad J. Achenbach, Lawrence J. Jennings, Chao Qi, Michael G. Ison, Judd F. Hultquist, Egon A. Ozer
EPI_ISL_1344624	Lighthouse Lab in Cambridge	Wellcome Sanger Institute for the COVID-19 Genomics UK (COG-UK) Consortium	Rob Howes, The Lighthouse Lab in Cambridge and Alex Alderton, Roberto Amato, Jeffrey Barrett, Sonia Goncalves, Ewan Harrison, David K. Jackson, Ian Johnston, Dominic Kwiatkowski, Cordelia Langford, John Sillitoe on behalf of the Wellcome Sanger Institute COVID-19 Surveillance Team
EPI_ISL_1347096	Microbiology Department, Laboratori Clínic Metropolitana Nord. Hospital Universitari Germans Trias i Pujol.	Can Ruti SARS-CoV-2 Sequencing Hub (HUGTiP/IrsiCaixa/IGTP)	Marc Noguera-Julian, Pilar Armengol, Ignacio Blanco, Antoni E Bordoy, Francesc Catala-Moll, Pere-Joan Cardona, Maria Casadellà, Cristina Casañ, Gemma Clara, Bonaventura Clotet, Cristina Esteban, Montserrat Giménez, Mercedes Guerrero, Anna Not, Roger Paredes, Mariona Parera, Verónica Saludes, Alba Sánchez, and Elisa Martró on behalf of the Can Ruti SARS-CoV-2 Sequencing Hub.
EPI_ISL_1347514	ZOTZ KLIMAS MVZ Düsseldorf-Centrum GbR ÜBAG für Labormedizin, Genetik, Zytologie, Pathologie	Center of Medical Microbiology, Virology, and Hospital Hygiene, University of Duesseldorf	Maximilian Damagnez;Alexander Dilthey;Patrick Finzer;Katrin Hoffmann;Torsten Houwaart;Lisanna Hülse;Malte Kohns Vasconcelos;Nadine Lübke;Jessica Nicolai;Klaus Pfeffer;Daniel Strelow;Jörg Timm;Andreas Walker;Tobias Wienemann;Rainer Zotz
EPI_ISL_1348120, EPI_ISL_1348160, EPI_ISL_1348170, EPI_ISL_1348171, EPI_ISL_1348180, EPI_ISL_1348182, EPI_ISL_1348186, EPI_ISL_1348193, EPI_ISL_1348202, EPI_ISL_1348222, EPI_ISL_1348238, EPI_ISL_1348240, EPI_ISL_1348244, EPI_ISL_1348249, EPI_ISL_1348250, EPI_ISL_1348254			
see above	Maryland Genomics, Institute for Genome Sciences, University of Maryland School of Medicine	Maryland Genomics, Institute for Genome Sciences, University of Maryland School of Medicine	Tallon, Luke J; Sadzewicz, Lisa D; Humphrys, Mike; Ott, Sandra; Roussey, Holly; Mehta, Aditya; Vavikolanu, Kranthi; Fraser, Claire M; Ravel, Jacques
EPI_ISL_1349209	MVZ Labor Krone GbR	Robert Koch Institute	unknown
EPI_ISL_1350094	Medizinische Laboratorien Düsseldorf	Robert Koch Institute	unknown
EPI_ISL_1350265, EPI_ISL_1350278	Procomcure Biotech Germany GmbH	Robert Koch Institute	unknown
EPI_ISL_1354965, EPI_ISL_1354983, EPI_ISL_1354995	Universitätsklinikum Heidelberg	Robert Koch Institute	unknown
EPI_ISL_1358210, EPI_ISL_1358211, EPI_ISL_1358213	Department of Virology I, National Institute of Infectious Diseases	Department of Veterinary Science, National Institute of Infectious Diseases	Yudai Kuroda, Tsukasa Yamamoto, Keita Ishijima, Tadaki Suzuki, Souichi Yamada, Shuetsu Fukushi, Ken Maeda
EPI_ISL_1363764, EPI_ISL_1363770, EPI_ISL_1363771, EPI_ISL_1363780	Maryland Genomics, Institute for Genome Sciences, University of Maryland School of Medicine	Maryland Genomics, Institute for Genome Sciences, University of Maryland School of Medicine	Tallon, Luke J; Sadzewicz, Lisa D; Humphrys, Mike; Ott, Sandra; Roussey, Holly; Mehta, Aditya; Vavikolanu, Kranthi; Fraser, Claire M; Ravel, Jacques
EPI_ISL_1364597	Virginia Division of Consolidated Laboratory Services	Virginia Division of Consolidated Laboratory Services	Virginia DCLS
EPI_ISL_1364670, EPI_ISL_1364723, EPI_ISL_1364739, EPI_ISL_1364758, EPI_ISL_1364774	Arizona State University	Arizona State University	Peter T. Skidmore, LaRinda A. Holland, Rabia Maqsood, Emily A. Kaelin, Nicholas J. Mellor, Kristina Buss, Joy M. Blain, Neal W Woodbury, Valerie Harris, Joshua LaBaer, Vel Murugan, Efrem S. Lim
EPI_ISL_1364903	Bundeswehrzentralkrankenhaus Koblenz	Bundeswehr Institute of Microbiology	Markus Antwerpen, Alexandra Rehn, Mathias Walter, Malena Bestehorn-Willmann, Mike Pillukat, Sabine Zange, Enrico Georgi, Roman Wölfel
EPI_ISL_1366176	UW Virology Lab	UW Virology Lab	Pavitra Roychoudhury, Hong Xie, Lasata Shrestha, Shah Mohamed Bakhash, Michelle Lin, Noah R. Baker, Sean Ellis, Saraswathi Sathees, Meei-Li Huang, Keith R Jerome, Alexander Greninger
EPI_ISL_1369463	CHUV	Laboratory of genomics and metagenomics, Institute of Microbiology, University Hospital Centre and University of Lausanne, Switzerland	Trestan Pillonel, Damien Jacot, Sébastien Aeby, Gilbert Greub, Claire Bertelli
EPI_ISL_1370682, EPI_ISL_1371431, EPI_ISL_1371459	Dutch COVID-19 response team	National Institute for Public Health and the Environment (RIVM)	Adam Meijer, Harry Vennema, Dirk Eggink, Jeroen Cremer, Sharon van den Brink, Bas van der Veer, AnneMarie van den Brandt, Florian Zwagemaker, Dennis Schmitz, Chantal Reusken, on behalf of the national COVID-19 response team
EPI_ISL_1371709, EPI_ISL_1371718	Virginia Division of Consolidated Laboratory Services	Virginia Division of Consolidated Laboratory Services	Virginia DCLS
EPI_ISL_1373319, EPI_ISL_1373322, EPI_ISL_1373323, EPI_ISL_1373328, EPI_ISL_1373355, EPI_ISL_1373356	MD PHL	MD PHL	Maryland Department of Health Laboratories Administration
EPI_ISL_1373445	UNC-CH COVID Surveillance Lab	Jeremy Wang	Jeremy Wang, Alexander Rubinsteyn, Melissa Miller, Corbin Jones, Amy James Loftis, Amir Barzin, Susan Fiscus
EPI_ISL_1373547	Vault Health	Minnesota Department of Health, Public Health Laboratory	Alexandra Lorentz, Jacob Garfin, Matt Plumb, and Xiong Wang
EPI_ISL_1374224	Lighthouse Lab in Cambridge	Wellcome Sanger Institute for the COVID-19 Genomics UK (COG-UK) Consortium	Rob Howes, The Lighthouse Lab in Cambridge and Alex Alderton, Roberto Amato, Jeffrey Barrett, Sonia Goncalves, Ewan Harrison, David K. Jackson, Ian Johnston, Dominic Kwiatkowski, Cordelia Langford, John Sillitoe on behalf of the Wellcome Sanger Institute COVID-19 Surveillance Team
EPI_ISL_1378794, EPI_ISL_1378795, EPI_ISL_1378796, EPI_ISL_1378800	Tempus	Grubaugh Lab - Yale School of Public Health	Joseph Fauver, Mallery Breban, Isabell Ott, Tara Alpert, Mary Petrone, Anderson Brito, Chantal Vogels, Annie Watkins, Chaney Kalinich, Jessica Rothman, Matthew J. MacKay, Gaurav Khullar, Jessica Metti, Joel T. Dudley, Megan Nash, Nike Beaubier, Christopher E. Mason, Nathan Grubaugh
EPI_ISL_1378822, EPI_ISL_1378823, EPI_ISL_1378828	Murphy Medical Association	Grubaugh Lab - Yale School of Public Health	Joseph Fauver, Mallery Breban, Isabell Ott, Tara Alpert, Mary Petrone, Anderson Brito, Chantal Vogels, Annie Watkins, Chaney Kalinich, Jessica Rothman, Caleb Neal, Eva Laszlo, Steven Murphy, Nathan Grubaugh

EPI_ISL_1379274, EPI_ISL_1379326, EPI_ISL_1379332	Hackensack Meridian Health	New York Genome Center	Michael Zody, Andre Corvelo, Dayna M. Oschwald, Samantha Fennessey, Tom Maniatis, Liang Chen, Jose Mediavilla, Marcus Cunningham, Kaelea Composto, Kar Chow, David Perlin, Barry Kreiswirth
EPI_ISL_1381885, EPI_ISL_1381886, EPI_ISL_1381887, EPI_ISL_1381888, EPI_ISL_1381889	Virginia Division of Consolidated Laboratory Services	Virginia Division of Consolidated Laboratory Services	Virginia DCLS
EPI_ISL_1382071	Department of Clinical Microbiology	GIGA Medical Genomics	Keith Durkin, Maria Artesi, Sébastien Bontems, Raphaël Boreux, Bouchra Boujemla, Nathalie Renotte, Cécile Meex, Pierrette Melin, Marie-Pierre Hayette, Vincent Bours
EPI_ISL_1382072	University of Liège COVID-19 testing center	GIGA Medical Genomics	Keith Durkin, Maria Artesi, Sébastien Bontems, Raphaël Boreux, Bouchra Boujemla, Nathalie Renotte, Cécile Meex, Pierrette Melin, Marie-Pierre Hayette, Vincent Bours
EPI_ISL_1382073	Department of Clinical Microbiology	GIGA Medical Genomics	Keith Durkin, Maria Artesi, Sébastien Bontems, Raphaël Boreux, Bouchra Boujemla, Nathalie Renotte, Cécile Meex, Pierrette Melin, Marie-Pierre Hayette, Vincent Bours
EPI_ISL_1382078, EPI_ISL_1382107, EPI_ISL_1382108	Virginia Division of Consolidated Laboratory Services	Virginia Division of Consolidated Laboratory Services	Virginia DCLS
EPI_ISL_1384922, EPI_ISL_1385148, EPI_ISL_1385149, EPI_ISL_1385293, EPI_ISL_1385357, EPI_ISL_1385422, EPI_ISL_1385507, EPI_ISL_1385539, EPI_ISL_1385593, EPI_ISL_1385714, EPI_ISL_1385764			
see above	Pandemic Response Lab - NYC	Pandemic Response Lab, R&D	Henry Lee, Michael Hammerling, Melissa Hopkins, Cybill del Castillo, Shinyoung Clair Kang, William Ward, Pradeep Bugga, Sol Rey, Dylan Law, Haiping Hao, Jon Laurent
EPI_ISL_1386037, EPI_ISL_1386038	Microbiology Department, Laboratori Clínic Metropolitana Nord. Hospital Universitari Germans Trias i Pujol.	Can Ruti SARS-CoV-2 Sequencing Hub (HUGTiP/IrsiCaixa/IGTP)	Marc Noguera-Julian, Pilar Armengol, Ignacio Blanco, Antoni E Bordoy, Francesc Catala-Moll, Pere-Joan Cardona, Maria Casadellà, Cristina Casañ, Gemma Clara, Bonaventura Clotet, Cristina Esteban, Montserrat Giménez, Mercedes Guerrero, Anna Not, Roger Paredes, Mariona Parera, Verónica Saludes, Alba Sánchez, and Elisa Martró on behalf of the Can Ruti SARS-CoV-2 Sequencing Hub.
EPI_ISL_1389336	Labo Analyses Med	National Reference Center for Viruses of Respiratory Infections, Institut Pasteur, Paris	Marion Barbet, Sylvie Behillil, Méline Bizard, Angela Brisebarre, Camille Capel, Louise Lefrançois, Etienne Simon-Lorière, Vincent Enouf, Maud Vanpeene, Sylvie van der Werf,Felloni Claire
EPI_ISL_1391378	DC Public Health Lab/ Dept. of Forensic Sciences	DC Public Health Lab/ Dept. of Forensic Sciences	Janis Doss, Scott Nguyen, Elizabeth Zelaya, Sarah Scott, Connie Maza, Monica Mann, Brittany Hamilton, David Payne, Jocelyn Hauser
EPI_ISL_1391992	Helix/Illumina	Centers for Disease Control and Prevention Division of Viral Diseases, Pathogen Discovery	Peter W. Cook, Dakota Howard, Dhwani Batra, Ben L. Rambo-Martin, Eileen de Feo, Jan Antico, Christine Tran, Matthew Tolentino, Shannon Wickline, Kim Gietzen, Brad Sickler, Jingtao Liu, Eric Allen, Phil Febbo, Summer Galloway, Nicole L. Washington, Simon White, Geraint Levan, Kelly Schiabor Barrett, Elizabeth Cirulli, Alexandre Bolze, Ary Ascencio, Charlotte Rivera-Garcia, Ryan Cho, Jason Nguyen, Sherry Wang, Jimmy Ramirez, Tyler Cassens, Efren Sandoval, Magnus Isaksson, William Lee, David Becker, Marc Laurent, James Lu, Clinton R. Paden, Suxiang Tong, Duncan MacCannell
EPI_ISL_1393289, EPI_ISL_1393291, EPI_ISL_1393292	Servicio de Microbiología Clínica (Complejo Hospitalario de Navarra, Pamplona)	Centro de Secuenciación NASERTIC	Carmen Ezpeleta Baquedano, Ana Navascués, Ana Miqueleiz
EPI_ISL_1394610	WESTCHESTER MEDICAL CENTER	Wadsworth Center, New York State Department of Health	Kirsten St. George, Daryl M. Lamson, Alexis Russel, Matthew Shudt, Melissa A Leisner, Jonathan Plitnick, Navjot Singh, John Kelly, Erasmus Schneider, Erica Lasek-Nesselquist
EPI_ISL_1397592, EPI_ISL_1397609	SUNY UPSTATE MEDICAL UNIVERSITY	Wadsworth Center, New York State Department of Health	Kirsten St. George, Daryl M. Lamson, Alexis Russel, Matthew Shudt, Melissa A Leisner, Jonathan Plitnick, Navjot Singh, John Kelly, Erasmus Schneider, Erica Lasek-Nesselquist
EPI_ISL_1397699	BIO-REFERENCE LABORATORIES	Wadsworth Center, New York State Department of Health	Kirsten St. George, Daryl M. Lamson, Alexis Russel, Matthew Shudt, Melissa A Leisner, Jonathan Plitnick, Navjot Singh, John Kelly, Erasmus Schneider, Erica Lasek-Nesselquist
EPI_ISL_1397787	SUNY UPSTATE MEDICAL UNIVERSITY	Wadsworth Center, New York State Department of Health	Kirsten St. George, Daryl M. Lamson, Alexis Russel, Matthew Shudt, Melissa A Leisner, Jonathan Plitnick, Navjot Singh, John Kelly, Erasmus Schneider, Erica Lasek-Nesselquist
EPI_ISL_1397853, EPI_ISL_1397869	ALBANY MEDICAL CENTER	Wadsworth Center, New York State Department of Health	Kirsten St. George, Daryl M. Lamson, Alexis Russel, Matthew Shudt, Melissa A Leisner, Jonathan Plitnick, Navjot Singh, John Kelly, Erasmus Schneider, Erica Lasek-Nesselquist
EPI_ISL_1398093	NORTHWELL HEALTH LABORATORIES	Wadsworth Center, New York State Department of Health	Kirsten St. George, Daryl M. Lamson, Alexis Russel, Matthew Shudt, Melissa A Leisner, Jonathan Plitnick, Navjot Singh, John Kelly, Erasmus Schneider, Erica Lasek-Nesselquist
EPI_ISL_1398144	Columbia University Irving Medical Center	Wadsworth Center, New York State Department of Health	Kirsten St. George, Daryl M. Lamson, Alexis Russel, Matthew Shudt, Melissa A Leisner, Jonathan Plitnick, Navjot Singh, John Kelly, Erasmus Schneider, Erica Lasek-Nesselquist
EPI_ISL_1398149	ALBANY MEDICAL CENTER	Wadsworth Center, New York State Department of Health	Kirsten St. George, Daryl M. Lamson, Alexis Russel, Matthew Shudt, Melissa A Leisner, Jonathan Plitnick, Navjot Singh, John Kelly, Erasmus Schneider, Erica Lasek-Nesselquist
EPI_ISL_1398159, EPI_ISL_1398160	SUNY UPSTATE MEDICAL UNIVERSITY	Wadsworth Center, New York State Department of Health	Kirsten St. George, Daryl M. Lamson, Alexis Russel, Matthew Shudt, Melissa A Leisner, Jonathan Plitnick, Navjot Singh, John Kelly, Erasmus Schneider, Erica Lasek-Nesselquist
EPI_ISL_1398257	KALEIDA CENTER FOR LABORATORY MEDICINE	Wadsworth Center, New York State Department of Health	Kirsten St. George, Daryl M. Lamson, Alexis Russel, Matthew Shudt, Melissa A Leisner, Jonathan Plitnick, Navjot Singh, John Kelly, Erasmus Schneider, Erica Lasek-Nesselquist
EPI_ISL_1398274, EPI_ISL_1398275, EPI_ISL_1398277	ALBANY MEDICAL CENTER	Wadsworth Center, New York State Department of Health	Kirsten St. George, Daryl M. Lamson, Alexis Russel, Matthew Shudt, Melissa A Leisner, Jonathan Plitnick, Navjot Singh, John Kelly, Erasmus Schneider, Erica Lasek-Nesselquist
EPI_ISL_1399579	Instituto Nacional de Saude (INSA)	Instituto Nacional de Saude (INSA)	Borges et al
EPI_ISL_1400837, EPI_ISL_1400849, EPI_ISL_1400860, EPI_ISL_1400862, EPI_ISL_1400872, EPI_ISL_1400875, EPI_ISL_1400877, EPI_ISL_1400878, EPI_ISL_1400879, EPI_ISL_1400887, EPI_ISL_1400892, EPI_ISL_1400893, EPI_ISL_1400895, EPI_ISL_1400896, EPI_ISL_1400897, EPI_ISL_1400898, EPI_ISL_1400912, EPI_ISL_1400913, EPI_ISL_1400922, EPI_ISL_1400932			
see above	Maryland Genomics, Institute for Genome Sciences, University of Maryland School of Medicine	Maryland Genomics, Institute for Genome Sciences, University of Maryland School of Medicine	Tallon, Luke J; Sadzewicz, Lisa D; Humphrys, Mike; Ott, Sandra; Roussey, Holly; Mehta, Aditya; Vavikolanu, Kranthi; Fraser, Claire M; Ravel, Jacques
EPI_ISL_1402805, EPI_ISL_1402832, EPI_ISL_1402861, EPI_ISL_1402870, EPI_ISL_1402876, EPI_ISL_1402914	Johns Hopkins Hospital Department of Pathology	Johns Hopkins Hospital Department of Pathology	C. Paul Morris, Chun Huai Luo, Adannaya Amadi, Matthew Schwartz, Heba H. Mostafa
EPI_ISL_1402987, EPI_ISL_1402996, EPI_ISL_1403023, EPI_ISL_1403032	Hospital of the University of Pennsylvania Molecular Pathology Lab	Bushman Lab - University of Pennsylvania	John Everett, Kyle Rodino, Shantan Reddy, Pascha Hokama, Aoife M. Roche, Young Hwang, Abigail Glascock, Scott Sherrill-Mix, Samantha A. Whiteside, Jevon Graham-Wooten, Layla A. Khatib, Ayannah S. Fitzgerald, Arupa Ganguly, Mike Feldman, Brendan Kelly, Ronald G. Collman and Frederic Bushman
EPI_ISL_1403814	University of Wisconsin-Madison AIDS Vaccine Research Laboratories	University of Wisconsin-Madison AIDS Vaccine Research Laboratories	Gage Moreno, Katarina Braun, et al. AIDS Vaccine Research Laboratories
EPI_ISL_1405030	NJDOH, Public Health and Environmental Laboratories	New Jersey Public Health and Environmental Laboratories (NJ PHEL)	Lindsey Bodnar, Shiv K. Verma, Dana Woell, Byeong Jeong
EPI_ISL_1405105, EPI_ISL_1405111, EPI_ISL_1405279	UW Virology Lab	UW Virology Lab	Pavitra Roychoudhury, Hong Xie, Lasata Shrestha, Shah Mohamed Bakhash, Michelle Lin, Noah R. Baker, Sean Ellis, Saraswathi Sathees, Meei-Li Huang, Keith R Jerome, Alexander Greninger
EPI_ISL_1405565, EPI_ISL_1405573, EPI_ISL_1405577	Johns Hopkins Hospital Department of Pathology	Johns Hopkins Hospital Department of Pathology	C. Paul Morris, Chun Huai Luo, Adannaya Amadi, Matthew Schwartz, Heba H. Mostafa
EPI_ISL_1405980, EPI_ISL_1406020	Microbiology Department, Laboratori Clínic Metropolitana Nord. Hospital Universitari Germans Trias i Pujol.	Can Ruti SARS-CoV-2 Sequencing Hub (HUGTiP/IrsiCaixa/IGTP)	Marc Noguera-Julian, Pilar Armengol, Ignacio Blanco, Antoni E Bordoy, Francesc Catala-Moll, Pere-Joan Cardona, Maria Casadellà, Cristina Casañ, Gemma Clara, Bonaventura Clotet, Cristina Esteban, Montserrat Giménez, Mercedes Guerrero, Anna Not, Roger Paredes, Mariona Parera, Verónica Saludes, Alba Sánchez, and Elisa Martró on behalf of the Can Ruti SARS-CoV-2 Sequencing Hub.

EPI_ISL_1407256, EPI_ISL_1407282, EPI_ISL_1407292, EPI_ISL_1413173, EPI_ISL_1413226, EPI_ISL_1413238, EPI_ISL_1413249, EPI_ISL_1413274, EPI_ISL_1413434, EPI_ISL_1413511	Broad Institute Clinical Research Sequencing Platform	Infectious Disease Program, Broad Institute of Harvard and MIT	Siddle,K.J., Adams,G., Pearlman,L., Gladden-Young,A., Vicente,G., Blumenstiel,B., DeFelice,M., Lee,M., McGovern,S., Lagerborg,K., Rudy,M., DeRuff,K., Carter,A., Normandin,E., Bauer,M., Reilly,S., Tomkins-Tinch,C., Loreth,C., Chaluvadi,S., Meldrim,J., Granger,B., Lemieux,J.E., Birren,B.W., Sabeti,P.C., Larkin,K., Dodge,S., Lennon,N., Madoff,L., Brown,C., Gallagher,G., Smole,S., Park,D.J., Gabriel,S., and MacInnis,B.L.
EPI_ISL_1413574	Rhode Island Department of Health	Infectious Disease Program, Broad Institute of Harvard and MIT	Siddle,K.J., Azevedo,K., Miller,A., Adams,G., Pearlman,L., Gladden-Young,A., Lagerborg,K., Rudy,M., DeRuff,K., Carter,A., Normandin,E., Bauer,M., Reilly,S., Tomkins-Tinch,C., Loreth,C., Chaluvadi,S., Lemieux,J.E., Birren,B.W., Sabeti,P.C., Huard,R., King,E., Park,D.J., and MacInnis,B.L.
EPI_ISL_1418775, EPI_ISL_1418776	Swedish national genomic surveillance program of SARS-CoV-2	The Public Health Agency of Sweden	Swedish national genomic surveillance program of SARS-CoV-2
EPI_ISL_1421495	Wisconsin State Laboratory of Hygiene Communicable Disease Division	Wisconsin State Laboratory of Hygiene Communicable Disease Division	Kelsey R. Florek, Abigail C. Shockey
EPI_ISL_1423188	WVU Rapid Development Laboratory	WVU and Marshall University Combined Genomics Core Facilities	"James Denvir, Peter Stoilov, Peter Perrotta, Wesley Kimble, Ryan Percifield"
EPI_ISL_1423220	Berkeley Medical Center	WVU and Marshall University Combined Genomics Core Facilities	"James Denvir, Peter Stoilov, Peter Perrotta, Wesley Kimble, Ryan Percifield"
EPI_ISL_1423274	LabCorp Dublin	WVU and Marshall University Combined Genomics Core Facilities	"James Denvir, Peter Stoilov, Peter Perrotta, Wesley Kimble, Ryan Percifield"
EPI_ISL_1423450	NYU Langone Health	Departments of Pathology and Medicine, New York University School of Medicine	Adriana Heguy, Dacia Dimartino, Emily Guzman, Christian Marier, Peter Meyn, Sitharam Ramaswami, Gael Westby, Paul Zappile, Yutong Zhang, Paolo Cotzia, Guiqing Wang
EPI_ISL_1425101, EPI_ISL_1425123, EPI_ISL_1425206, EPI_ISL_1425253, EPI_ISL_1425458, EPI_ISL_1425490	Department of Infectious Diseases, Kobe Institute of Health	Department of Infectious Diseases, Kobe Institute of Health	Ryohei Nomoto, Noriko Nakanishi, Tomotada Iwamoto, Tsuyoshi Sekizuka, Kentaro Itokawa, Rina Tanaka, Masanori Hashino, Makoto Kuroda
EPI_ISL_1425698	Pathogen Genomics Center, National Institute of Infectious Diseases	Pathogen Genomics Center, National Institute of Infectious Diseases	Tsuyoshi Sekizuka, Kentaro Itokawa, Rina Tanaka, Masanori Hashino, Makoto Kuroda
EPI_ISL_1425719	SARS-CoV-2 testing team, National Institute of Infectious Diseases	Pathogen Genomics Center, National Institute of Infectious Diseases	Tsuyoshi Sekizuka, Kentaro Itokawa, Rina Tanaka, Masanori Hashino, Koichi Ishikawa, Midori Nakamura-Hoshi, Shigeru Kusagawa, Makoto Kuroda
EPI_ISL_1425720	SARS-CoV-2 testing team, National Institute of Infectious Diseases	Pathogen Genomics Center, National Institute of Infectious Diseases	Tsuyoshi Sekizuka, Kentaro Itokawa, Rina Tanaka, Masanori Hashino, Eri Nakayama, Motohiko Ogawa, Takahiro Maeki, Shigeru Tanjima, Chang-Kweng Lim, Makoto Kuroda
EPI_ISL_1425721, EPI_ISL_1425722, EPI_ISL_1425724	Pathogen Genomics Center, National Institute of Infectious Diseases	Pathogen Genomics Center, National Institute of Infectious Diseases	Tsuyoshi Sekizuka, Kentaro Itokawa, Rina Tanaka, Masanori Hashino, Makoto Kuroda
EPI_ISL_1425725, EPI_ISL_1425726, EPI_ISL_1425727, EPI_ISL_1425728, EPI_ISL_1425729, EPI_ISL_1425730, EPI_ISL_1425731, EPI_ISL_1425732, EPI_ISL_1425733, EPI_ISL_1425734	SARS-CoV-2 testing team, National Institute of Infectious Diseases	Pathogen Genomics Center, National Institute of Infectious Diseases	Tsuyoshi Sekizuka, Kentaro Itokawa, Rina Tanaka, Masanori Hashino, Shigeru Tanjima, Eri Nakayama, Takahiro Maeki, Motohiko Ogawa, Chang-Kweng Lim, Makoto Kuroda
EPI_ISL_1425735, EPI_ISL_1425736, EPI_ISL_1425737, EPI_ISL_1425738, EPI_ISL_1425739, EPI_ISL_1425748	Saitama Prefectural Institute of Public Health	Pathogen Genomics Center, National Institute of Infectious Diseases	Tsuyoshi Sekizuka, Kentaro Itokawa, Rina Tanaka, Masanori Hashino, Makoto Kuroda
EPI_ISL_1426646	Pathogen Genomics Center, National Institute of Infectious Diseases	Pathogen Genomics Center, National Institute of Infectious Diseases	Tsuyoshi Sekizuka, Kentaro Itokawa, Rina Tanaka, Masanori Hashino, Makoto Kuroda
EPI_ISL_1426648, EPI_ISL_1426649	SARS-CoV-2 testing team, National Institute of Infectious Diseases	Pathogen Genomics Center, National Institute of Infectious Diseases	Tsuyoshi Sekizuka, Kentaro Itokawa, Rina Tanaka, Masanori Hashino, Hidemasa Izumiya, Hideyuki Takahashi, Masatomo Morita, Nobuo Koizumi, , Makoto Kuroda
EPI_ISL_1426650, EPI_ISL_1426651, EPI_ISL_1426652	Saitama Prefectural Institute of Public Health	Pathogen Genomics Center, National Institute of Infectious Diseases	Tsuyoshi Sekizuka, Kentaro Itokawa, Rina Tanaka, Masanori Hashino, Makoto Kuroda
EPI_ISL_1426653, EPI_ISL_1426654, EPI_ISL_1426655, EPI_ISL_1426656, EPI_ISL_1426657, EPI_ISL_1426658	Chiba Prefectural Institute of Public Health	Pathogen Genomics Center, National Institute of Infectious Diseases	Tsuyoshi Sekizuka, Kentaro Itokawa, Rina Tanaka, Masanori Hashino, Makoto Kuroda
EPI_ISL_1426659, EPI_ISL_1426660	Pathogen Genomics Center, National Institute of Infectious Diseases	Pathogen Genomics Center, National Institute of Infectious Diseases	Tsuyoshi Sekizuka, Kentaro Itokawa, Rina Tanaka, Masanori Hashino, Makoto Kuroda
EPI_ISL_1427689, EPI_ISL_1427690, EPI_ISL_1427691, EPI_ISL_1427692	Gunma Prefectural Institute of Public Health and Environmental Sciences	Pathogen Genomics Center, National Institute of Infectious Diseases	Tsuyoshi Sekizuka, Kentaro Itokawa, Rina Tanaka, Masanori Hashino, Makoto Kuroda
EPI_ISL_1427693	SARS-CoV-2 testing team, National Institute of Infectious Diseases	Pathogen Genomics Center, National Institute of Infectious Diseases	Tsuyoshi Sekizuka, Kentaro Itokawa, Rina Tanaka, Masanori Hashino, Daisuke Kobayashi, Kento Fukano, Yasuyuki Morishima, Takanobu Kato, , Makoto Kuroda
EPI_ISL_1427694, EPI_ISL_1427695, EPI_ISL_1427696, EPI_ISL_1427697, EPI_ISL_1427698, EPI_ISL_1427699, EPI_ISL_1427700, EPI_ISL_1427701, EPI_ISL_1427702, EPI_ISL_1427703, EPI_ISL_1427704, EPI_ISL_1427705, EPI_ISL_1427706, EPI_ISL_1427707, EPI_ISL_1427708, EPI_ISL_1427709, EPI_ISL_1427710, EPI_ISL_1427711, EPI_ISL_1427712, EPI_ISL_1427713, EPI_ISL_1427714			
see above	Pathogen Genomics Center, National Institute of Infectious Diseases	Pathogen Genomics Center, National Institute of Infectious Diseases	Tsuyoshi Sekizuka, Kentaro Itokawa, Rina Tanaka, Masanori Hashino, Makoto Kuroda
EPI_ISL_1427716	Sakai City Institute of Public Health	Pathogen Genomics Center, National Institute of Infectious Diseases	Tsuyoshi Sekizuka, Kentaro Itokawa, Rina Tanaka, Masanori Hashino, Makoto Kuroda
EPI_ISL_1427717, EPI_ISL_1427718	Chiba Prefectural Institute of Public Health	Pathogen Genomics Center, National Institute of Infectious Diseases	Tsuyoshi Sekizuka, Kentaro Itokawa, Rina Tanaka, Masanori Hashino, Makoto Kuroda
EPI_ISL_1428665, EPI_ISL_1428694, EPI_ISL_1428695, EPI_ISL_1428696, EPI_ISL_1428697, EPI_ISL_1428698, EPI_ISL_1428699, EPI_ISL_1428700, EPI_ISL_1428701, EPI_ISL_1428702, EPI_ISL_1428703, EPI_ISL_1428704, EPI_ISL_1428705, EPI_ISL_1428706, EPI_ISL_1428707, EPI_ISL_1428708, EPI_ISL_1428709, EPI_ISL_1428710, EPI_ISL_1428711, EPI_ISL_1428712, EPI_ISL_1428713, EPI_ISL_1428714, EPI_ISL_1428715, EPI_ISL_1428716, EPI_ISL_1428717, EPI_ISL_1428718, EPI_ISL_1428719, EPI_ISL_1428720, EPI_ISL_1428721, EPI_ISL_1428722, EPI_ISL_1428723, EPI_ISL_1428724, EPI_ISL_1428725, EPI_ISL_1428726, EPI_ISL_1428727, EPI_ISL_1428728, EPI_ISL_1428729, EPI_ISL_1428730, EPI_ISL_1428731, EPI_ISL_1428732, EPI_ISL_1428733, EPI_ISL_1428734, EPI_ISL_1428735, EPI_ISL_1428736, EPI_ISL_1428737, EPI_ISL_1428738, EPI_ISL_1428739, EPI_ISL_1428740, EPI_ISL_1428741, EPI_ISL_1428742, EPI_ISL_1428743, EPI_ISL_1428744, EPI_ISL_1428745, EPI_ISL_1428746, EPI_ISL_1428747, EPI_ISL_1428748, EPI_ISL_1428749, EPI_ISL_1428750, EPI_ISL_1428751, EPI_ISL_1428752, EPI_ISL_1428753, EPI_ISL_1428754, EPI_ISL_1428755, EPI_ISL_1428756, EPI_ISL_1428757, EPI_ISL_1428758, EPI_ISL_1428759, EPI_ISL_1428760, EPI_ISL_1428761, EPI_ISL_1428762, EPI_ISL_1428763, EPI_ISL_1428764, EPI_ISL_1428765, EPI_ISL_1428766, EPI_ISL_1428767, EPI_ISL_1428768, EPI_ISL_1428769, EPI_ISL_1428770, EPI_ISL_1428771, EPI_ISL_1428777, EPI_ISL_1428779, EPI_ISL_1429609, EPI_ISL_1429635, EPI_ISL_1429637, EPI_ISL_1429638			
see above	Pathogen Genomics Center, National Institute of Infectious Diseases	Pathogen Genomics Center, National Institute of Infectious Diseases	Tsuyoshi Sekizuka, Kentaro Itokawa, Rina Tanaka, Masanori Hashino, Makoto Kuroda
EPI_ISL_1429639	Toyama Institute of Health	Pathogen Genomics Center, National Institute of Infectious Diseases	Tsuyoshi Sekizuka, Kentaro Itokawa, Rina Tanaka, Masanori Hashino, Makoto Kuroda
EPI_ISL_1430685	Niigata Prefectural Institute of Public Health and Environmental Sciences	Pathogen Genomics Center, National Institute of Infectious Diseases	Tsuyoshi Sekizuka, Kentaro Itokawa, Rina Tanaka, Masanori Hashino, Makoto Kuroda
EPI_ISL_1430702, EPI_ISL_1430703, EPI_ISL_1430704, EPI_ISL_1430735, EPI_ISL_1430741, EPI_ISL_1430742, EPI_ISL_1430743, EPI_ISL_1430744, EPI_ISL_1430745, EPI_ISL_1430746, EPI_ISL_1430747, EPI_ISL_1430748			

see above	Pathogen Genomics Center, National Institute of Infectious Diseases	Pathogen Genomics Center, National Institute of Infectious Diseases	Tsuyoshi Sekizuka, Kentaro Itokawa, Rina Tanaka, Masanori Hashino, Makoto Kuroda
EPI_ISL_1430749, EPI_ISL_1430750	Kanagawa Prefectural Institute of Public Health	Pathogen Genomics Center, National Institute of Infectious Diseases	Tsuyoshi Sekizuka, Kentaro Itokawa, Rina Tanaka, Masanori Hashino, Makoto Kuroda
EPI_ISL_1430751, EPI_ISL_1430752, EPI_ISL_1430753, EPI_ISL_1430754	Pathogen Genomics Center, National Institute of Infectious Diseases	Pathogen Genomics Center, National Institute of Infectious Diseases	Tsuyoshi Sekizuka, Kentaro Itokawa, Rina Tanaka, Masanori Hashino, Makoto Kuroda
EPI_ISL_1430755, EPI_ISL_1430756, EPI_ISL_1430757, EPI_ISL_1430758, EPI_ISL_1430759, EPI_ISL_1430760, EPI_ISL_1430761	SARS-CoV-2 testing team, National Institute of Infectious Diseases	Pathogen Genomics Center, National Institute of Infectious Diseases	Tsuyoshi Sekizuka, Kentaro Itokawa, Rina Tanaka, Masanori Hashino, Daisuke Kobayashi, Kousho Wakae, Hussein H. Aly, Takanobu Kato, , Makoto Kuroda
EPI_ISL_1430763, EPI_ISL_1430764, EPI_ISL_1430765, EPI_ISL_1430766, EPI_ISL_1430767, EPI_ISL_1430768, EPI_ISL_1430769, EPI_ISL_1430770, EPI_ISL_1430771, EPI_ISL_1430772	Saitama Prefectural Institute of Public Health	Pathogen Genomics Center, National Institute of Infectious Diseases	Tsuyoshi Sekizuka, Kentaro Itokawa, Rina Tanaka, Masanori Hashino, Makoto Kuroda
EPI_ISL_1430773, EPI_ISL_1430774, EPI_ISL_1430775, EPI_ISL_1430776, EPI_ISL_1430777, EPI_ISL_1430778, EPI_ISL_1430779, EPI_ISL_1430780, EPI_ISL_1430781, EPI_ISL_1430782, EPI_ISL_1430783, EPI_ISL_1430784, EPI_ISL_1430785, EPI_ISL_1430786, EPI_ISL_1430787, EPI_ISL_1430788, EPI_ISL_1430789, EPI_ISL_1430790, EPI_ISL_1430791, EPI_ISL_1430792, EPI_ISL_1430793, EPI_ISL_1430794, EPI_ISL_1430795, EPI_ISL_1430796, EPI_ISL_1430797, EPI_ISL_1430798, EPI_ISL_1430799, EPI_ISL_1430800, EPI_ISL_1430801, EPI_ISL_1430802, EPI_ISL_1430803, EPI_ISL_1430804, EPI_ISL_1430805, EPI_ISL_1430806, EPI_ISL_1430807, EPI_ISL_1430808, EPI_ISL_1430809, EPI_ISL_1430810, EPI_ISL_1430811, EPI_ISL_1430812, EPI_ISL_1430813, EPI_ISL_1430815, EPI_ISL_1430816, EPI_ISL_1430817, EPI_ISL_1430818, EPI_ISL_1430819, EPI_ISL_1430820, EPI_ISL_1430821, EPI_ISL_1430822, EPI_ISL_1430823			
see above	Pathogen Genomics Center, National Institute of Infectious Diseases	Pathogen Genomics Center, National Institute of Infectious Diseases	Tsuyoshi Sekizuka, Kentaro Itokawa, Rina Tanaka, Masanori Hashino, Makoto Kuroda
EPI_ISL_1430824	Osaka Institute of Public Health, Morinomiya Center	Pathogen Genomics Center, National Institute of Infectious Diseases	Tsuyoshi Sekizuka, Kentaro Itokawa, Rina Tanaka, Masanori Hashino, Makoto Kuroda
EPI_ISL_1430825, EPI_ISL_1430826, EPI_ISL_1430827	SARS-CoV-2 testing team, National Institute of Infectious Diseases	Pathogen Genomics Center, National Institute of Infectious Diseases	Tsuyoshi Sekizuka, Kentaro Itokawa, Rina Tanaka, Masanori Hashino, Yoshihiro Kaku, Yasutaka Hoshino, Chikako Shimokawa, Eunsil Park, Tsuguto Fujimoto, Makoto Kuroda
EPI_ISL_1430829, EPI_ISL_1430830, EPI_ISL_1430833, EPI_ISL_1430834, EPI_ISL_1430835, EPI_ISL_1430836, EPI_ISL_1430837, EPI_ISL_1430838, EPI_ISL_1430840, EPI_ISL_1430841, EPI_ISL_1430842, EPI_ISL_1430843, EPI_ISL_1430844, EPI_ISL_1430845, EPI_ISL_1430846, EPI_ISL_1430847, EPI_ISL_1430848			
see above	Pathogen Genomics Center, National Institute of Infectious Diseases	Pathogen Genomics Center, National Institute of Infectious Diseases	Tsuyoshi Sekizuka, Kentaro Itokawa, Rina Tanaka, Masanori Hashino, Makoto Kuroda
EPI_ISL_1430849, EPI_ISL_1430850, EPI_ISL_1430851, EPI_ISL_1430852, EPI_ISL_1430853, EPI_ISL_1430854	Niigata Prefectural Institute of Public Health and Environmental Sciences	Pathogen Genomics Center, National Institute of Infectious Diseases	Tsuyoshi Sekizuka, Kentaro Itokawa, Rina Tanaka, Masanori Hashino, Makoto Kuroda
EPI_ISL_1430855, EPI_ISL_1430856, EPI_ISL_1430857, EPI_ISL_1430858, EPI_ISL_1430859, EPI_ISL_1430860, EPI_ISL_1430861, EPI_ISL_1430862, EPI_ISL_1430863, EPI_ISL_1430864, EPI_ISL_1431590, EPI_ISL_1431591, EPI_ISL_1431592, EPI_ISL_1431593, EPI_ISL_1431594, EPI_ISL_1431607, EPI_ISL_1431608, EPI_ISL_1431609, EPI_ISL_1431610, EPI_ISL_1431621, EPI_ISL_1431622, EPI_ISL_1431627, EPI_ISL_1431628, EPI_ISL_1431629, EPI_ISL_1431630, EPI_ISL_1431631, EPI_ISL_1431632, EPI_ISL_1431633, EPI_ISL_1431634, EPI_ISL_1431635, EPI_ISL_1431636, EPI_ISL_1431637, EPI_ISL_1431638, EPI_ISL_1431639, EPI_ISL_1431640, EPI_ISL_1431641			
see above	Pathogen Genomics Center, National Institute of Infectious Diseases	Pathogen Genomics Center, National Institute of Infectious Diseases	Tsuyoshi Sekizuka, Kentaro Itokawa, Rina Tanaka, Masanori Hashino, Makoto Kuroda
EPI_ISL_1431642, EPI_ISL_1431643, EPI_ISL_1431644, EPI_ISL_1431645, EPI_ISL_1431646, EPI_ISL_1431647, EPI_ISL_1431648, EPI_ISL_1431649, EPI_ISL_1431650, EPI_ISL_1431651, EPI_ISL_1431652, EPI_ISL_1431653, EPI_ISL_1431654, EPI_ISL_1431655, EPI_ISL_1431656, EPI_ISL_1431657, EPI_ISL_1431658, EPI_ISL_1431659, EPI_ISL_1431660, EPI_ISL_1431661, EPI_ISL_1431662, EPI_ISL_1431663, EPI_ISL_1431664, EPI_ISL_1431665, EPI_ISL_1431666, EPI_ISL_1431667, EPI_ISL_1431668, EPI_ISL_1431669, EPI_ISL_1431670, EPI_ISL_1431671, EPI_ISL_1431672, EPI_ISL_1431673, EPI_ISL_1431674, EPI_ISL_1431675, EPI_ISL_1431676, EPI_ISL_1431677, EPI_ISL_1431678, EPI_ISL_1431679, EPI_ISL_1431680, EPI_ISL_1431681, EPI_ISL_1431682, EPI_ISL_1431683, EPI_ISL_1431684, EPI_ISL_1431685, EPI_ISL_1431686, EPI_ISL_1431687, EPI_ISL_1431688, EPI_ISL_1431690, EPI_ISL_1431691, EPI_ISL_1431692, EPI_ISL_1431693, EPI_ISL_1431694, EPI_ISL_1431695, EPI_ISL_1431696, EPI_ISL_1431697, EPI_ISL_1431698, EPI_ISL_1431699, EPI_ISL_1431700, EPI_ISL_1431701, EPI_ISL_1431702, EPI_ISL_1431703, EPI_ISL_1431704, EPI_ISL_1431705, EPI_ISL_1431706, EPI_ISL_1431707, EPI_ISL_1431708			
see above	Chiba Prefectural Institute of Public Health	Pathogen Genomics Center, National Institute of Infectious Diseases	Tsuyoshi Sekizuka, Kentaro Itokawa, Rina Tanaka, Masanori Hashino, Makoto Kuroda
EPI_ISL_1431709, EPI_ISL_1431710, EPI_ISL_1431711, EPI_ISL_1431712, EPI_ISL_1431713, EPI_ISL_1431714, EPI_ISL_1431715, EPI_ISL_1431716, EPI_ISL_1431717, EPI_ISL_1431718	Pathogen Genomics Center, National Institute of Infectious Diseases	Pathogen Genomics Center, National Institute of Infectious Diseases	Tsuyoshi Sekizuka, Kentaro Itokawa, Rina Tanaka, Masanori Hashino, Makoto Kuroda
EPI_ISL_1431719	Osaka Institute of Public Health, Morinomiya Center	Pathogen Genomics Center, National Institute of Infectious Diseases	Tsuyoshi Sekizuka, Kentaro Itokawa, Rina Tanaka, Masanori Hashino, Makoto Kuroda
EPI_ISL_1431720, EPI_ISL_1431721, EPI_ISL_1431722, EPI_ISL_1431723	Pathogen Genomics Center, National Institute of Infectious Diseases	Pathogen Genomics Center, National Institute of Infectious Diseases	Tsuyoshi Sekizuka, Kentaro Itokawa, Rina Tanaka, Masanori Hashino, Makoto Kuroda
EPI_ISL_1431724	Niigata Prefectural Institute of Public Health and Environmental Sciences	Pathogen Genomics Center, National Institute of Infectious Diseases	Tsuyoshi Sekizuka, Kentaro Itokawa, Rina Tanaka, Masanori Hashino, Makoto Kuroda
EPI_ISL_1431725	Pathogen Genomics Center, National Institute of Infectious Diseases	Pathogen Genomics Center, National Institute of Infectious Diseases	Tsuyoshi Sekizuka, Kentaro Itokawa, Rina Tanaka, Masanori Hashino, Makoto Kuroda
EPI_ISL_1431726	Chiba Prefectural Institute of Public Health	Pathogen Genomics Center, National Institute of Infectious Diseases	Tsuyoshi Sekizuka, Kentaro Itokawa, Rina Tanaka, Masanori Hashino, Makoto Kuroda
EPI_ISL_1431727, EPI_ISL_1431728, EPI_ISL_1431729	Pathogen Genomics Center, National Institute of Infectious Diseases	Pathogen Genomics Center, National Institute of Infectious Diseases	Tsuyoshi Sekizuka, Kentaro Itokawa, Rina Tanaka, Masanori Hashino, Makoto Kuroda
EPI_ISL_1431730	Niigata Prefectural Institute of Public Health and Environmental Sciences	Pathogen Genomics Center, National Institute of Infectious Diseases	Tsuyoshi Sekizuka, Kentaro Itokawa, Rina Tanaka, Masanori Hashino, Makoto Kuroda
EPI_ISL_1431731, EPI_ISL_1431732, EPI_ISL_1431733, EPI_ISL_1431734, EPI_ISL_1431735	Pathogen Genomics Center, National Institute of Infectious Diseases	Pathogen Genomics Center, National Institute of Infectious Diseases	Tsuyoshi Sekizuka, Kentaro Itokawa, Rina Tanaka, Masanori Hashino, Makoto Kuroda
EPI_ISL_1431736	Gunma Prefectural Institute of Public Health and Environmental Sciences	Pathogen Genomics Center, National Institute of Infectious Diseases	Tsuyoshi Sekizuka, Kentaro Itokawa, Rina Tanaka, Masanori Hashino, Makoto Kuroda
EPI_ISL_1431737, EPI_ISL_1431738	Pathogen Genomics Center, National Institute of Infectious Diseases	Pathogen Genomics Center, National Institute of Infectious Diseases	Tsuyoshi Sekizuka, Kentaro Itokawa, Rina Tanaka, Masanori Hashino, Makoto Kuroda
EPI_ISL_1431739, EPI_ISL_1431740	Saitama Prefectural Institute of Public Health	Pathogen Genomics Center, National Institute of Infectious Diseases	Tsuyoshi Sekizuka, Kentaro Itokawa, Rina Tanaka, Masanori Hashino, Makoto Kuroda
EPI_ISL_1431741, EPI_ISL_1431742, EPI_ISL_1431743, EPI_ISL_1431744	Pathogen Genomics Center, National Institute of Infectious Diseases	Pathogen Genomics Center, National Institute of Infectious Diseases	Tsuyoshi Sekizuka, Kentaro Itokawa, Rina Tanaka, Masanori Hashino, Makoto Kuroda
EPI_ISL_1431745, EPI_ISL_1431746, EPI_ISL_1431747, EPI_ISL_1431748	Saitama Prefectural Institute of Public Health	Pathogen Genomics Center, National Institute of Infectious Diseases	Tsuyoshi Sekizuka, Kentaro Itokawa, Rina Tanaka, Masanori Hashino, Makoto Kuroda

EPI_ISL_1431749, EPI_ISL_1431750, EPI_ISL_1431751, EPI_ISL_1431752, EPI_ISL_1431753, EPI_ISL_1431754, EPI_ISL_1431755, EPI_ISL_1431756, EPI_ISL_1431757, EPI_ISL_1431758, EPI_ISL_1431759, EPI_ISL_1431760, EPI_ISL_1431761, EPI_ISL_1431762, EPI_ISL_1431763, EPI_ISL_1431764			
see above	Chiba Prefectural Institute of Public Health	Pathogen Genomics Center, National Institute of Infectious Diseases	Tsuyoshi Sekizuka, Kentaro Itokawa, Rina Tanaka, Masanori Hashino, Makoto Kuroda
EPI_ISL_1431765, EPI_ISL_1431766, EPI_ISL_1431767, EPI_ISL_1431768, EPI_ISL_1431769, EPI_ISL_1431770, EPI_ISL_1431771, EPI_ISL_1431772, EPI_ISL_1431773, EPI_ISL_1431774, EPI_ISL_1431775, EPI_ISL_1431776, EPI_ISL_1431777, EPI_ISL_1431778, EPI_ISL_1431779, EPI_ISL_1431780, EPI_ISL_1431781, EPI_ISL_1431782, EPI_ISL_1431783, EPI_ISL_1431784, EPI_ISL_1431785, EPI_ISL_1431786, EPI_ISL_1431787, EPI_ISL_1431788, EPI_ISL_1431789, EPI_ISL_1431790, EPI_ISL_1431791, EPI_ISL_1431792, EPI_ISL_1431793, EPI_ISL_1431794, EPI_ISL_1431795, EPI_ISL_1431796, EPI_ISL_1431797, EPI_ISL_1431798, EPI_ISL_1431799, EPI_ISL_1431800, EPI_ISL_1431801			
see above	Pathogen Genomics Center, National Institute of Infectious Diseases	Pathogen Genomics Center, National Institute of Infectious Diseases	Tsuyoshi Sekizuka, Kentaro Itokawa, Rina Tanaka, Masanori Hashino, Makoto Kuroda
EPI_ISL_1431802, EPI_ISL_1431803, EPI_ISL_1431804	Gunma Prefectural Institute of Public Health and Environmental Sciences	Pathogen Genomics Center, National Institute of Infectious Diseases	Tsuyoshi Sekizuka, Kentaro Itokawa, Rina Tanaka, Masanori Hashino, Makoto Kuroda
EPI_ISL_1431805	Toyama Institute of Health	Pathogen Genomics Center, National Institute of Infectious Diseases	Tsuyoshi Sekizuka, Kentaro Itokawa, Rina Tanaka, Masanori Hashino, Makoto Kuroda
EPI_ISL_1431806, EPI_ISL_1431807, EPI_ISL_1431808, EPI_ISL_1431809, EPI_ISL_1431810, EPI_ISL_1431811, EPI_ISL_1431812, EPI_ISL_1431813, EPI_ISL_1431814, EPI_ISL_1431815, EPI_ISL_1431816, EPI_ISL_1431817, EPI_ISL_1431818, EPI_ISL_1431819, EPI_ISL_1431820, EPI_ISL_1431821, EPI_ISL_1431822, EPI_ISL_1431823			
see above	Pathogen Genomics Center, National Institute of Infectious Diseases	Pathogen Genomics Center, National Institute of Infectious Diseases	Tsuyoshi Sekizuka, Kentaro Itokawa, Rina Tanaka, Masanori Hashino, Makoto Kuroda
EPI_ISL_1431824, EPI_ISL_1431825, EPI_ISL_1431826, EPI_ISL_1431827, EPI_ISL_1431828	Chiba Prefectural Institute of Public Health	Pathogen Genomics Center, National Institute of Infectious Diseases	Tsuyoshi Sekizuka, Kentaro Itokawa, Rina Tanaka, Masanori Hashino, Makoto Kuroda
EPI_ISL_1431829, EPI_ISL_1431830, EPI_ISL_1431831, EPI_ISL_1431832, EPI_ISL_1431833, EPI_ISL_1431834, EPI_ISL_1431835, EPI_ISL_1431836, EPI_ISL_1431837, EPI_ISL_1431838, EPI_ISL_1431839, EPI_ISL_1431840, EPI_ISL_1431841, EPI_ISL_1431842, EPI_ISL_1431843, EPI_ISL_1431844, EPI_ISL_1431845, EPI_ISL_1431846, EPI_ISL_1431847, EPI_ISL_1431848, EPI_ISL_1431849, EPI_ISL_1431850, EPI_ISL_1431851, EPI_ISL_1431852, EPI_ISL_1431853, EPI_ISL_1431854, EPI_ISL_1431855, EPI_ISL_1431856, EPI_ISL_1431857, EPI_ISL_1431858, EPI_ISL_1431859, EPI_ISL_1431860, EPI_ISL_1431861, EPI_ISL_1431862, EPI_ISL_1431863, EPI_ISL_1431864, EPI_ISL_1431865, EPI_ISL_1431866, EPI_ISL_1431867, EPI_ISL_1431868, EPI_ISL_1431869, EPI_ISL_1431870, EPI_ISL_1431871, EPI_ISL_1431872, EPI_ISL_1431873, EPI_ISL_1431874, EPI_ISL_1431875, EPI_ISL_1431876, EPI_ISL_1431877, EPI_ISL_1431878, EPI_ISL_1431879, EPI_ISL_1431880, EPI_ISL_1431881, EPI_ISL_1431882, EPI_ISL_1431883, EPI_ISL_1431884, EPI_ISL_1431885, EPI_ISL_1431886, EPI_ISL_1431887, EPI_ISL_1431888, EPI_ISL_1431889, EPI_ISL_1431890, EPI_ISL_1431891, EPI_ISL_1431892, EPI_ISL_1431893, EPI_ISL_1431894, EPI_ISL_1431895, EPI_ISL_1431896, EPI_ISL_1431897, EPI_ISL_1431898, EPI_ISL_1431899, EPI_ISL_1431900, EPI_ISL_1431901, EPI_ISL_1431902, EPI_ISL_1431903, EPI_ISL_1431904, EPI_ISL_1431905, EPI_ISL_1431906, EPI_ISL_1431907, EPI_ISL_1431908, EPI_ISL_1431909, EPI_ISL_1431910, EPI_ISL_1431911, EPI_ISL_1431912, EPI_ISL_1431913, EPI_ISL_1431914, EPI_ISL_1431915, EPI_ISL_1431916, EPI_ISL_1431917, EPI_ISL_1431918, EPI_ISL_1431919, EPI_ISL_1431920, EPI_ISL_1431921, EPI_ISL_1431922, EPI_ISL_1431923, EPI_ISL_1431924, EPI_ISL_1431925, EPI_ISL_1431926, EPI_ISL_1431927, EPI_ISL_1431928, EPI_ISL_1431929, EPI_ISL_1431930, EPI_ISL_1431931, EPI_ISL_1431932, EPI_ISL_1431933, EPI_ISL_1431934, EPI_ISL_1431935, EPI_ISL_1431936, EPI_ISL_1431937, EPI_ISL_1431938, EPI_ISL_1431939, EPI_ISL_1431940, EPI_ISL_1431941, EPI_ISL_1431942, EPI_ISL_1431943, EPI_ISL_1431944, EPI_ISL_1431945, EPI_ISL_1431946, EPI_ISL_1431947, EPI_ISL_1431948, EPI_ISL_1431949, EPI_ISL_1431950, EPI_ISL_1431951, EPI_ISL_1431952, EPI_ISL_1431953, EPI_ISL_1431954, EPI_ISL_1431955, EPI_ISL_1431956, EPI_ISL_1431957, EPI_ISL_1431958, EPI_ISL_1431959, EPI_ISL_1431960, EPI_ISL_1431961, EPI_ISL_1431962, EPI_ISL_1431963, EPI_ISL_1431964, EPI_ISL_1431965, EPI_ISL_1431966, EPI_ISL_1431967, EPI_ISL_1431968, EPI_ISL_1431969, EPI_ISL_1431970, EPI_ISL_1431971, EPI_ISL_1431972, EPI_ISL_1431973, EPI_ISL_1431974, EPI_ISL_1431975, EPI_ISL_1431976, EPI_ISL_1431977, EPI_ISL_1431978, EPI_ISL_1431979, EPI_ISL_1431980, EPI_ISL_1431981, EPI_ISL_1431982, EPI_ISL_1431983, EPI_ISL_1431984, EPI_ISL_1431985, EPI_ISL_1431986, EPI_ISL_1431987, EPI_ISL_1431988, EPI_ISL_1431989, EPI_ISL_1431990, EPI_ISL_1431991, EPI_ISL_1431992, EPI_ISL_1431993, EPI_ISL_1431994, EPI_ISL_1431995, EPI_ISL_1431996, EPI_ISL_1431997, EPI_ISL_1431998, EPI_ISL_1431999, EPI_ISL_1432000, EPI_ISL_1432001, EPI_ISL_1432002, EPI_ISL_1432003, EPI_ISL_1432004			
see above	Pathogen Genomics Center, National Institute of Infectious Diseases	Pathogen Genomics Center, National Institute of Infectious Diseases	Tsuyoshi Sekizuka, Kentaro Itokawa, Rina Tanaka, Masanori Hashino, Makoto Kuroda
EPI_ISL_1432005	Toyama Institute of Health	Pathogen Genomics Center, National Institute of Infectious Diseases	Tsuyoshi Sekizuka, Kentaro Itokawa, Rina Tanaka, Masanori Hashino, Makoto Kuroda
EPI_ISL_1432006, EPI_ISL_1433318, EPI_ISL_1433320, EPI_ISL_1433322, EPI_ISL_1433324, EPI_ISL_1433326, EPI_ISL_1433328, EPI_ISL_1433330, EPI_ISL_1433331, EPI_ISL_1433333, EPI_ISL_1433335, EPI_ISL_1433337, EPI_ISL_1433339, EPI_ISL_1433341, EPI_ISL_1433343, EPI_ISL_1433345, EPI_ISL_1433348, EPI_ISL_1433350, EPI_ISL_1433352, EPI_ISL_1433353, EPI_ISL_1433355, EPI_ISL_1433357, EPI_ISL_1433359, EPI_ISL_1433361, EPI_ISL_1433363, EPI_ISL_1433365, EPI_ISL_1433367, EPI_ISL_1433369, EPI_ISL_1433371, EPI_ISL_1433373			
see above	Pathogen Genomics Center, National Institute of Infectious Diseases	Pathogen Genomics Center, National Institute of Infectious Diseases	Tsuyoshi Sekizuka, Kentaro Itokawa, Rina Tanaka, Masanori Hashino, Makoto Kuroda
EPI_ISL_1433375, EPI_ISL_1433377, EPI_ISL_1433379, EPI_ISL_1433381, EPI_ISL_1433383, EPI_ISL_1433385, EPI_ISL_1433387, EPI_ISL_1433389	Ibaraki Prefectural Institute of Public Health	Pathogen Genomics Center, National Institute of Infectious Diseases	Tsuyoshi Sekizuka, Kentaro Itokawa, Rina Tanaka, Masanori Hashino, Makoto Kuroda
EPI_ISL_1433764, EPI_ISL_1434074, EPI_ISL_1434130, EPI_ISL_1434325	Labor Dr. Wisplinghoff - Köln	Robert Koch Institute	unknown
EPI_ISL_1434432, EPI_ISL_1434434, EPI_ISL_1434436, EPI_ISL_1434438, EPI_ISL_1434440, EPI_ISL_1434442, EPI_ISL_1434445, EPI_ISL_1434447, EPI_ISL_1434449, EPI_ISL_1434451, EPI_ISL_1434453, EPI_ISL_1434455, EPI_ISL_1434457, EPI_ISL_1434459, EPI_ISL_1434460, EPI_ISL_1434462, EPI_ISL_1434464, EPI_ISL_1434466, EPI_ISL_1434468, EPI_ISL_1434470, EPI_ISL_1434472, EPI_ISL_1434474, EPI_ISL_1434476, EPI_ISL_1434478, EPI_ISL_1434480, EPI_ISL_1434482, EPI_ISL_1434484, EPI_ISL_1434486, EPI_ISL_1434488, EPI_ISL_1434490, EPI_ISL_1434491, EPI_ISL_1434493, EPI_ISL_1434495, EPI_ISL_1434497, EPI_ISL_1434499, EPI_ISL_1434501, EPI_ISL_1434503, EPI_ISL_1434505, EPI_ISL_1434507, EPI_ISL_1434509, EPI_ISL_1434511, EPI_ISL_1434513, EPI_ISL_1434515, EPI_ISL_1434517, EPI_ISL_1434519, EPI_ISL_1434522, EPI_ISL_1434523, EPI_ISL_1434525, EPI_ISL_1434527, EPI_ISL_1434529			
see above	Ibaraki Prefectural Institute of Public Health	Ibaraki Prefectural Institute of Public Health	Keiko Goto, Tsuyoshi Sekizuka, Kentaro Itokawa, Rina Tanaka, Masanori Hashino, Makoto Kuroda
EPI_ISL_1435375	MVZ Dr. Eberhard & Partner Dortmund	Robert Koch Institute	unknown
EPI_ISL_1437816, EPI_ISL_1440277, EPI_ISL_1440317, EPI_ISL_1440341, EPI_ISL_1440363, EPI_ISL_1440417, EPI_ISL_1440424, EPI_ISL_1440427, EPI_ISL_1440428, EPI_ISL_1440434, EPI_ISL_1440436, EPI_ISL_1440468, EPI_ISL_1440469, EPI_ISL_1440483, EPI_ISL_1440556, EPI_ISL_1442604, EPI_ISL_1442609, EPI_ISL_1442613, EPI_ISL_1442633, EPI_ISL_1442711, EPI_ISL_1442732, EPI_ISL_1442744			
see above	SYNLAB MVZ Weiden	Robert Koch Institute	unknown
EPI_ISL_1442956, EPI_ISL_1442957, EPI_ISL_1442958, EPI_ISL_1442959, EPI_ISL_1442960, EPI_ISL_1442961, EPI_ISL_1442962, EPI_ISL_1442963	Gunma Prefectural Institute of Public Health and Environmental Sciences	Gunma Prefectural Institute of Public Health and Environmental Sciences	Tsuyoshi Sekizuka, Kentaro Itokawa, Rina Tanaka, Masanori Hashino, Makoto Kuroda, Hiroyuki Tsukagoshi, Daisuke Shinoda, Mariko Saito
EPI_ISL_1443315	Clinical Molecular Microbiology Laboratory, UNC Hospital	Dirk Dittmer	Justin T. Landis , Razia Moorad , Brent A. Eason, Melissa B. Miller, Linda Pluta, Dirk Dittmer, Angelica Juarez, Cecilia Thompson, Shawn Hawken, Cameroon Grant, Evelyn Hoffman, Patricio Cano, Jason Wong, Carolina Caro-Vegas, Ryan McNamara, Blossom Damania.
EPI_ISL_1443429	Lab voor klinische biologie	Lab voor klinische biologie	Marija Janevska, Hannelore Hamerlinck, Bruno Verhasselt
EPI_ISL_1444820, EPI_ISL_1444822, EPI_ISL_1444826, EPI_ISL_1444827, EPI_ISL_1444828, EPI_ISL_1444850, EPI_ISL_1444856, EPI_ISL_1444859, EPI_ISL_1444867, EPI_ISL_1444901	Maryland Genomics, Institute for Genome Sciences, University of Maryland School of Medicine	Maryland Genomics, Institute for Genome Sciences, University of Maryland School of Medicine	Tallon, Luke J; Sadzewicz, Lisa D; Humphrys, Mike; Ott, Sandra; Roussey, Holly; Mehta, Aditya; Vavikolanu, Kranthi; Fraser, Claire M; Ravel, Jacques
EPI_ISL_1446224	Texas Children's Hospital	Texas Children's Microbiome Center	Ruth Ann Luna, Jennifer K. Spinler, James Dunn, James Versalovic, Ila Singh
EPI_ISL_1447099	DOHMH PHL	New York City Public Health Laboratory	Jade Wang, et al.
EPI_ISL_1447112	DOHMH Fort Greene	New York City Public Health Laboratory	Jade Wang, et al.
EPI_ISL_1447184	DOHMH PHL	New York City Public Health Laboratory	Jade Wang, et al.
EPI_ISL_1447185	DOHMH Corona	New York City Public Health Laboratory	Jade Wang, et al.
EPI_ISL_1447186	DOHMH Central Harlem	New York City Public Health Laboratory	Jade Wang, et al.
EPI_ISL_1447627, EPI_ISL_1447647, EPI_ISL_1447660, EPI_ISL_1447669,	Yale Clinical Virology Lab	Grubaugh Lab - Yale School of Public Health	Joseph Fauver, Mallery Breban, Isabel Ott, Tara Alpert, Mary Petrone, Anderson Brito, Chantal Vogels, Annie Watkins, Chaney Kalinich, Jessica Rothman, Marie L. Landry, Nathan Grubaugh

EPI_ISL_1447670			
EPI_ISL_1448034, EPI_ISL_1448041	Maryland Genomics, Institute for Genome Sciences, University of Maryland School of Medicine	Maryland Genomics, Institute for Genome Sciences, University of Maryland School of Medicine	Tallon, Luke J; Sadzewicz, Lisa D; Humphrys, Mike; Ott, Sandra; Roussey, Holly; Mehta, Aditya; Vavikolanu, Kranthi; Fraser, Claire M; Ravel, Jacques
EPI_ISL_1448375	UW Virology Lab	UW Virology Lab	Pavitra Roychoudhury, Hong Xie, Lasata Shrestha, Shah Mohamed Bakhash, Michelle Lin, Noah R. Baker, Sean Ellis, Saraswathi Sathees, Meei-Li Huang, Keith R Jerome, Alexander Greninger
EPI_ISL_1454536	Lighthouse Lab in Cambridge	Wellcome Sanger Institute for the COVID-19 Genomics UK (COG-UK) Consortium	Rob Howes, The Lighthouse Lab in Cambridge and Alex Alderton, Roberto Amato, Jeffrey Barrett, Sonia Goncalves, Ewan Harrison, David K. Jackson, Ian Johnston, Dominic Kwiatkowski, Cordelia Langford, John Sillitoe on behalf of the Wellcome Sanger Institute COVID-19 Surveillance Team
EPI_ISL_1457561	Dutch COVID-19 response team	National Institute for Public Health and the Environment (RIVM)	Adam Meijer, Harry Vennema, Dirk Eggink, Jeroen Cremer, Sharon van den Brink, Bas van der Veer, AnneMarie van den Brandt, Lisa Wijsman, Kim Freriks, Ryanne Jaarsma, Euníce Then, Jolienke Hardeman, Lynn Aarts, Sanne Bos, Melissa van Tuil, Robert Kohl, Linda van de Nes, Sjoerd Kuiling, James Groot, Florian Zwagemaker, Dennis Schmitz, Annelies Kroneman, Karim Hajji, Chantal Reusken, on behalf of the national COVID-19 response team
EPI_ISL_1461295	ALBANY MEDICAL CENTER	Wadsworth Center, New York State Department of Health	Kirsten St. George, Daryl M. Lamson, Alexis Russell, Matthew Shudt, Melissa A Leisner, Jonathan Plitnick, Catharine Prussing, Navjot Singh, John Kelly, Erasmus Schneider, Erica Lasek-Nesselquist
EPI_ISL_1461365, EPI_ISL_1461366, EPI_ISL_1461367	TEMPUS LABS INC	Wadsworth Center, New York State Department of Health	Kirsten St. George, Daryl M. Lamson, Alexis Russell, Matthew Shudt, Melissa A Leisner, Jonathan Plitnick, Catharine Prussing, Navjot Singh, John Kelly, Erasmus Schneider, Erica Lasek-Nesselquist
EPI_ISL_1461644	CHU NIMES	CHU NIMES	Stephan Robin, Marie-josée Carles, Sophie Bravo, Agathe Boudet
EPI_ISL_1461683	Virginia Division of Consolidated Laboratory Services	Virginia Division of Consolidated Laboratory Services	Virginia DCLS
EPI_ISL_1462500	SUNY UPSTATE MEDICAL UNIVERSITY	Wadsworth Center, New York State Department of Health	Kirsten St. George, Daryl M. Lamson, Alexis Russell, Matthew Shudt, Melissa A Leisner, Jonathan Plitnick, Catharine Prussing, Navjot Singh, John Kelly, Erasmus Schneider, Erica Lasek-Nesselquist
EPI_ISL_1465674, EPI_ISL_1465677	THE MARY IMOGENE BASSETT HOSPITAL	Wadsworth Center, New York State Department of Health	Kirsten St. George, Daryl M. Lamson, Alexis Russell, Matthew Shudt, Melissa A Leisner, Jonathan Plitnick, Catharine Prussing, Navjot Singh, John Kelly, Erasmus Schneider, Erica Lasek-Nesselquist
EPI_ISL_1465691	SUNY UPSTATE MEDICAL UNIVERSITY	Wadsworth Center, New York State Department of Health	Kirsten St. George, Daryl M. Lamson, Alexis Russell, Matthew Shudt, Melissa A Leisner, Jonathan Plitnick, Catharine Prussing, Navjot Singh, John Kelly, Erasmus Schneider, Erica Lasek-Nesselquist
EPI_ISL_1465865	TEMPUS LABS INC	Wadsworth Center, New York State Department of Health	Kirsten St. George, Daryl M. Lamson, Alexis Russell, Matthew Shudt, Melissa A Leisner, Jonathan Plitnick, Catharine Prussing, Navjot Singh, John Kelly, Erasmus Schneider, Erica Lasek-Nesselquist
EPI_ISL_1468561	Johns Hopkins Hospital Department of Pathology	Johns Hopkins Hospital Department of Pathology	C. Paul Morris, Chun Huai Luo, Adannaya Amadi, Matthew Schwartz, Heba H. Mostafa
EPI_ISL_1468693	Altius Institute	Seattle Flu Study	Deborah A. Nickerson, Chris D. Frazar, Jover Lee, Benjamin Pelle, Erica Ryke, Matthew Richardson, Amanda Adler, Elisabeth Brandstetter, Peter D. Han, Kairsten Fay, Misja Ilcisin, Kirsten Lacombe, Thomas R. Sibley, Melissa Truong, Caitlin R. Wolf, Ryan Alexander, Daniel Bates, Rebecca Bruders, Stephanie DeBaun, Clem Green, Muhammad Halimun, Jessica Halow, Kneshay Harper, Matt Hartman, Andrew Meuser, Alex Nguyen, Truong Nguyen, Sofia Olsson, Sadie Patraw, Hannah Petersen, Tobias Ragoczy, Joshua Richards, Jacob Rodriguez, John Stamatoyannopoulos, Julia Wald, Olivia Waltner, Michael Boeckh, Janet A. Englund, Michael Famulare, Barry R. Lutz, Mark J. Rieder, Lea M. Starita, Matthew Thompson, Helen Y. Chu, Jay Shendure, Trevor Bedford
EPI_ISL_1470380, EPI_ISL_1470381, EPI_ISL_1470382, EPI_ISL_1470383, EPI_ISL_1470397, EPI_ISL_1470398, EPI_ISL_1470399, EPI_ISL_1470400, EPI_ISL_1470401	Kentucky State Public Health Lab	Kentucky State Public Health Lab	Stephanie Lunn, Karim George, Joshua Tobias, William Grooms, Vaneet Arora, Matthew Johnson, Rachel Zinner, Rhonda Lucas
EPI_ISL_1470810, EPI_ISL_1471392, EPI_ISL_1471594, EPI_ISL_1471660, EPI_ISL_1471681, EPI_ISL_1471711, EPI_ISL_1471937, EPI_ISL_1471985	Pandemic Response Lab - NYC	Pandemic Response Lab, R&D	Henry Lee, Michael Hammerling, Melissa Hopkins, Cybill del Castillo, Shinyoung Clair Kang, William Ward, Pradeep Bugga, Sol Rey, Dylan Law, Haiping Hao, Jon Laurent
EPI_ISL_1472331, EPI_ISL_1472332, EPI_ISL_1472333, EPI_ISL_1472334	Hokkaido Institute of Public Health	Hokkaido Institute of Public Health	Yuta Ohno, Kazuhiro Okubo, Hiroki Yamaguchi, Tetsuya Ikeda, Tsuyoshi Sekizuka, Kentaro Itokawa, Rina Tanaka, Masanori Hashino, Makoto Kuroda
EPI_ISL_1481630, EPI_ISL_1481650, EPI_ISL_1482193, EPI_ISL_1482261	Fulgent Genetics	Centers for Disease Control and Prevention Division of Viral Diseases, Pathogen Discovery	Dakota Howard, Dhwani Batra, Peter W. Cook, Kara Moser, Adrian Paskey, Jason Caravas, Benjamin Rambo-Martin, Shatavia Morrison, Christopher Gulvick, Scott Sammons, Yvette Unoarumhi, Darlene Wagner, Matthew Schmerer, Harry Gao, Mickey Li, John Gao, Joseph Fierro, Benafsh Sapra, Becky Tsai, Yan Meng, Doreen Ng, James Xie, Clinton R. Paden, Duncan MacCannell
EPI_ISL_1482275	SUNY UPSTATE MEDICAL UNIVERSITY	Wadsworth Center, New York State Department of Health	Kirsten St. George, Daryl M. Lamson, Alexis Russell, Matthew Shudt, Melissa A Leisner, Jonathan Plitnick, Catharine Prussing, Navjot Singh, John Kelly, Erasmus Schneider, Erica Lasek-Nesselquist
EPI_ISL_1482447	BOSTON HEART DIAGNOSTICS CORP	Wadsworth Center, New York State Department of Health	Kirsten St. George, Daryl M. Lamson, Alexis Russell, Matthew Shudt, Melissa A Leisner, Jonathan Plitnick, Catharine Prussing, Navjot Singh, John Kelly, Erasmus Schneider, Erica Lasek-Nesselquist
EPI_ISL_1482919	MUSC Molecular Pathology Laboratory	MUSC Molecular Pathology Laboratory	Julie W. Hirschhorn, W. Bailey Glen Jr, Dariusz Pytel, Jaclyn Dunne, Kristen Maurer, Frederick S. Nolte
EPI_ISL_1483301	Tampa General Hospital Esoteric Lab	Tampa General Hospital Esoteric Research & Development Lab	Grant Vestal, Deanna Becker, Dominic Uy, Vicki Healer, Amorce Lima, Suzane Silbert
EPI_ISL_1489702, EPI_ISL_1489703, EPI_ISL_1489705, EPI_ISL_1489716	NJDOH, Public Health and Environmental Laboratories	New Jersey Public Health and Environmental Laboratories (NJ PHEL)	Lindsey Bodnar, Shiv K. Verma, Dana Woell, Byeong Jeong
EPI_ISL_1490666, EPI_ISL_1490714	The Caribbean Public Health Agency	Carrington Lab, Department of PreClinical Sciences, Faculty of Medical Sciences, The University of the West Indies	Nikita S. D. Sahadeo, Arianne Brown-Jordan, Sarah Hill, Vernie Ramkissoon, Roshan Parasram, Naresh Nandram, Avery Hinds, Jerome Foster, Stanley Giddings, Karla Georges, Marsha Ivey, Rahul Naidu, Risha Singh, SueMin Nathaniel, Rajini Haraksingh, Jaya Jayaraman, Chinna Chinnadurai, Adesh Ramsubhag, Nuno Faria, Oliver Pybus, Christopher Oura, Gabriel Escobar, Christine V. F. Carrington
EPI_ISL_1491347, EPI_ISL_1491353	Kentucky State Public Health Lab	Kentucky State Public Health Lab	Stephanie Lunn, Karim George, Joshua Tobias, William Grooms, Vaneet Arora, Matthew Johnson, Rachel Zinner, Rhonda Lucas
EPI_ISL_1491789, EPI_ISL_1491800, EPI_ISL_1491835, EPI_ISL_1491864, EPI_ISL_1491889, EPI_ISL_1491921	Aegis Sciences Corporation	Centers for Disease Control and Prevention Division of Viral Diseases, Pathogen Discovery	Dakota Howard, Dhwani Batra, Peter W. Cook, Kara Moser, Adrian Paskey, Jason Caravas, Benjamin Rambo-Martin, Shatavia Morrison, Christopher Gulvick, Scott Sammons, Yvette Unoarumhi, Darlene Wagner, Matthew Schmerer, Cyndi Clark, Patrick Campbell, Rob Case, Vikramsinha Ghorpade, Holly Houdeshell, Ola Kvalvaag, Dillon Nall, Ethan Sanders, Alec Vest, Shaun Westlund, Matthew Hardison, Clinton R. Paden, Duncan MacCannell
EPI_ISL_1491962, EPI_ISL_1491973, EPI_ISL_1492016	Quest Diagnostics Incorporated	Centers for Disease Control and Prevention Division of Viral Diseases, Pathogen Discovery	Dakota Howard, Dhwani Batra, Peter W. Cook, Kara Moser, Adrian Paskey, Jason Caravas, Benjamin Rambo-Martin, Shatavia Morrison, Christopher Gulvick, Scott Sammons, Yvette Unoarumhi, Darlene Wagner, Matthew Schmerer, S. H. Rosenthal, A. Gerasimova, R. M. Kagan, B. Anderson, M. Hua, Y. Liu, L.E. Bernstein, K.E. Livingston, A. Perez, I. A. Shlyakhter, R. V. Rolando, R. Owen, P. Tanpaiboon, F. Lacbawan, Clinton R. Paden, Duncan MacCannell
EPI_ISL_1493191, EPI_ISL_1493213	MD PHL	MD PHL	Maryland Department of Health Laboratories Administration
EPI_ISL_1494559, EPI_ISL_1494572, EPI_ISL_1494608	Virginia Division of Consolidated Laboratory Services	Virginia Division of Consolidated Laboratory Services	Virginia DCLS
EPI_ISL_1495528, EPI_ISL_1495529, EPI_ISL_1495530, EPI_ISL_1495531, EPI_ISL_1495532, EPI_ISL_1495533, EPI_ISL_1495534, EPI_ISL_1495535	Klinikum Wels-Grieskirchen	Bergthaler laboratory, CeMM Research Center for Molecular Medicine of the Austrian Academy of Sciences	Lukas Endler, Anna Schedl, Fabian Amman, Petr Triska, Thomas Penz, Benedikt Agerer, Maelle Le Moing, Michael Schuster, Bekir Erguner, Jan Laine, Martin Senekowitsch, Christoph Bock, Andreas Bergthaler

EPI_ISL_1495633, EPI_ISL_1495635, EPI_ISL_1495636, EPI_ISL_1495653, EPI_ISL_1495692, EPI_ISL_1495703, EPI_ISL_1495704, EPI_ISL_1495712, EPI_ISL_1495715	Austrian Agency for Health and Food Safety (AGES)	Bergthaler laboratory, CeMM Research Center for Molecular Medicine of the Austrian Academy of Sciences	Lukas Endler, Anna Schedl, Fabian Amman, Petr Triska, Thomas Penz, Benedikt Agerer, Maelle Le Moing, Michael Schuster, Bekir Erguner, Jan Laine, Martin Senekowitsch, Christoph Bock, Andreas Bergthaler
EPI_ISL_1495719, EPI_ISL_1495720, EPI_ISL_1495742, EPI_ISL_1495743, EPI_ISL_1495744, EPI_ISL_1495745, EPI_ISL_1495746, EPI_ISL_1495747, EPI_ISL_1495748	Elling group, Institute of Molecular Biotechnology (IMBA)	Bergthaler laboratory, CeMM Research Center for Molecular Medicine of the Austrian Academy of Sciences	Lukas Endler, Anna Schedl, Fabian Amman, Petr Triska, Thomas Penz, Benedikt Agerer, Maelle Le Moing, Michael Schuster, Bekir Erguner, Jan Laine, Martin Senekowitsch, Christoph Bock, Andreas Bergthaler
EPI_ISL_1498095, EPI_ISL_1498262, EPI_ISL_1498379	The Caribbean Public Health Agency	Carrington Lab, Department of PreClinical Sciences, Faculty of Medical Sciences, The University of the West Indies	Nikita S. D. Sahadeo, Arianne Brown-Jordan, Sarah Hill, Vernie Ramkissoon, Roshan Parasram, Naresh Nandram, Avery Hinds, Jerome Foster, Stanley Giddings, Karla Georges, Marsha Ivey, Rahul Naidu, Risha Singh, SueMin Nathaniel, Rajini Haraksingh, Jaya Jayaraman, Chinna Chinnadurai, Adesh Ramsubhag, Nuno Faria, Oliver Pybus, Christopher Oura, Gabriel Escobar, Christine V. F. Carrington
EPI_ISL_1499113, EPI_ISL_1499115	Trinidad Public Health Laboratory	Carrington Lab, Department of PreClinical Sciences, Faculty of Medical Sciences, The University of the West Indies	Nikita S. D. Sahadeo, Arianne Brown-Jordan, Sarah Hill, Vernie Ramkissoon, Roshan Parasram, Naresh Nandram, Avery Hinds, Jerome Foster, Stanley Giddings, Karla Georges, Marsha Ivey, Rahul Naidu, Risha Singh, SueMin Nathaniel, Rajini Haraksingh, Jaya Jayaraman, Chinna Chinnadurai, Adesh Ramsubhag, Nuno Faria, Oliver Pybus, Christopher Oura, Gabriel Escobar, Christine V. F. Carrington
EPI_ISL_1500250, EPI_ISL_1500251, EPI_ISL_1500349, EPI_ISL_1500369, EPI_ISL_1500419, EPI_ISL_1500425	Maryland Genomics, Institute for Genome Sciences, University of Maryland School of Medicine	Maryland Genomics, Institute for Genome Sciences, University of Maryland School of Medicine	Tallon, Luke J; Sadzewicz, Lisa D; Humphrys, Mike; Ott, Sandra; Roussey, Holly; Mehta, Aditya; Vavikolanu, Kranthi; Fraser, Claire M; Ravel, Jacques
EPI_ISL_1500772	TGen North	TGen North	"Jolene Bowers, Heather Centner, Chris French, Hayley Yaglom, Ashlyn Pfeiffer, Darrin Lemmer, Dave Engelthaler, The Arizona COVID Genomics Union (ACGU)"
EPI_ISL_1503466, EPI_ISL_1503602, EPI_ISL_1503717, EPI_ISL_1503762, EPI_ISL_1503778, EPI_ISL_1503780	Massachusetts State Public Health Laboratory	Massachusetts State Public Health Laboratory	Andrew Lang, Timelia Fink, Glen Gallagher, Sandra Smole
EPI_ISL_1506119	MD Laboratories	Los Angeles County PHL	P. Hemarajata et al.
EPI_ISL_1508984	Kaiser Regional	Santa Clara County Public Health Laboratory	Santa Clara County Public Health Department
EPI_ISL_1509050, EPI_ISL_1509053, EPI_ISL_1509054, EPI_ISL_1509085, EPI_ISL_1509087	Department of Laboratory Medicine, Clinical Center, National Institutes of Health	Laboratory of Parasitic Diseases, Systems Genomics Section, National Institute of Allergy and Infectious Diseases, National Institutes of Health	Allison Roder, Stephanie Banakis, Matthew Chung, Jung-ho Youn, Rachel Mercado, Wei Wang, Tara Palmore, Michael Bell, Heike Bailin, Jessica McCormick-Ell, Sanchita Das, Jennifer Kwan, Elodie Ghedin
EPI_ISL_1509144, EPI_ISL_1509192	Yale Clinical Virology Lab	Grubaugh Lab - Yale School of Public Health	Joseph Fauver, Mallery Breban, Isabell Ott, Tara Alpert, Mary Petrone, Anderson Brito, Chantal Vogels, Annie Watkins, Chaney Kalinich, Jessica Rothman, Marie L. Landry, Nathan Grubaugh
EPI_ISL_1509929	"AR Dept. of Health-PHL, Molecular Diagnostics"	Genomics and Discovery, Respiratory Viruses Branch, Division of Viral Diseases, Centers for Disease Control and Prevention	Yan Li, Ying Tao, Jing Zhang, Brian Lynch, Anna Kelleher, Anna Montmayeur, Krista Queen, Anna Uehara, Peter Cook, Rachel Marine, Mark Burroughs, Han Jia Justin Ng, Clinton R. Paden, Haibin Wang, Justin Lee, Adam Retchless, Suxiang Tong
EPI_ISL_1511565, EPI_ISL_1511592, EPI_ISL_1511595	Hospital of the University of Pennsylvania Molecular Pathology Lab	Bushman Lab - University of Pennsylvania	John Everett, Kyle Rodino, Shantan Reddy, Pascha Hokama, Aoife M. Roche, Young Hwang, Abigail Glascock, Scott Sherrill-Mix, Samantha A. Whiteside, Jevon Graham-Wooten, Layla A. Khatib, Ayannah S. Fitzgerald, Arupa Ganguly, Mike Feldman, Brendan Kelly, Ronald G. Collman and Frederic Bushman
EPI_ISL_1511665, EPI_ISL_1511689, EPI_ISL_1511784, EPI_ISL_1511796, EPI_ISL_1511831, EPI_ISL_1511844	Quest Diagnostics Incorporated	Centers for Disease Control and Prevention Division of Viral Diseases, Pathogen Discovery	Dakota Howard, Dhwani Batra, Peter W. Cook, Kara Moser, Adrian Paskey, Jason Caravas, Benjamin Rambo-Martin, Shatavia Morrison, Christopher Gulvick, Scott Sammons, Yvette Unoarumhi, Darlene Wagner, Matthew Schmerer, S. H. Rosenthal, A. Gerasimova, R. M. Kagan, B. Anderson, M. Hua, Y. Liu, L.E. Bernstein, K.E. Livingston, A. Perez, I. A. Shlyakhter, R. V. Rolando, R. Owen, P. Tanpaiboon, F. Lacbawan, Clinton R. Paden, Duncan MacCannell
EPI_ISL_1511944, EPI_ISL_1511993, EPI_ISL_1511994, EPI_ISL_1512015, EPI_ISL_1512142, EPI_ISL_1512147, EPI_ISL_1512159, EPI_ISL_1512192, EPI_ISL_1512228, EPI_ISL_1512256, EPI_ISL_1512262, EPI_ISL_1512265, EPI_ISL_1512276, EPI_ISL_1512288, EPI_ISL_1512290, EPI_ISL_1512297, EPI_ISL_1512361, EPI_ISL_1512375, EPI_ISL_1512406, EPI_ISL_1512455, EPI_ISL_1512483, EPI_ISL_1512507, EPI_ISL_1512517, EPI_ISL_1512534, EPI_ISL_1512555, EPI_ISL_1512625, EPI_ISL_1512627, EPI_ISL_1512638, EPI_ISL_1512655			
see above	Helix/Illumina	Centers for Disease Control and Prevention Division of Viral Diseases, Pathogen Discovery	Dakota Howard, Dhwani Batra, Peter W. Cook, Kara Moser, Adrian Paskey, Jason Caravas, Benjamin Rambo-Martin, Shatavia Morrison, Christopher Gulvick, Scott Sammons, Yvette Unoarumhi, Darlene Wagner, Matthew Schmerer, Eileen de Feo, Jan Antico, Christine Tran, Matthew Tolentino, Shannon Wickline, Kim Gietzen, Brad Sickler, Jingtao Liu, Eric Allen, Phil Febbo, Nicole L. Washington, Simon White, Geraint Levan, Kelly Schiabor Barrett, Elizabeth Cirulli, Alexandre Bolze, Ary Ascencio, Charlotte Rivera-Garcia, Ryan Cho, Jason Nguyen, Sherry Wang, Jimmy Ramirez, Tyler Cassens, Efren Sandoval, Magnus Isaksson, William Lee, David Becker, Marc Laurent, James Lu, Clinton R. Paden, Duncan MacCannell
EPI_ISL_1512833, EPI_ISL_1512838, EPI_ISL_1512905, EPI_ISL_1512920, EPI_ISL_1512952, EPI_ISL_1512954, EPI_ISL_1513028, EPI_ISL_1513031, EPI_ISL_1513080, EPI_ISL_1513097	Infinity Biologix	Centers for Disease Control and Prevention Division of Viral Diseases, Pathogen Discovery	Dakota Howard, Dhwani Batra, Peter W. Cook, Kara Moser, Adrian Paskey, Jason Caravas, Benjamin Rambo-Martin, Shatavia Morrison, Christopher Gulvick, Scott Sammons, Yvette Unoarumhi, Darlene Wagner, Matthew Schmerer, Christian Bixby, Yihe Wang, Jonathan Schultz, Chirayu Goswami, Russ Hager, Robin Grimwood, Clinton R. Paden, Duncan MacCannell
EPI_ISL_1513157, EPI_ISL_1513230, EPI_ISL_1513281, EPI_ISL_1513282, EPI_ISL_1513285, EPI_ISL_1513292, EPI_ISL_1513355, EPI_ISL_1513444, EPI_ISL_1513463, EPI_ISL_1513469, EPI_ISL_1513492, EPI_ISL_1513512			
see above	Aegis Sciences Corporation	Centers for Disease Control and Prevention Division of Viral Diseases, Pathogen Discovery	Dakota Howard, Dhwani Batra, Peter W. Cook, Kara Moser, Adrian Paskey, Jason Caravas, Benjamin Rambo-Martin, Shatavia Morrison, Christopher Gulvick, Scott Sammons, Yvette Unoarumhi, Darlene Wagner, Matthew Schmerer, Cyndi Clark, Patrick Campbell, Rob Case, Vikramsinha Ghorpade, Holly Houdeshell, Ola Kvalvaag, Dillon Nall, Ethan Sanders, Alec Vest, Shaun Westlund, Matthew Hardison, Clinton R. Paden, Duncan MacCannell
EPI_ISL_1513800, EPI_ISL_1513921, EPI_ISL_1513933, EPI_ISL_1513950, EPI_ISL_1513951, EPI_ISL_1513952, EPI_ISL_1513983, EPI_ISL_1514018, EPI_ISL_1514041, EPI_ISL_1514097, EPI_ISL_1514221, EPI_ISL_1514225, EPI_ISL_1514314, EPI_ISL_1514340, EPI_ISL_1514388, EPI_ISL_1514495, EPI_ISL_1514520, EPI_ISL_1514607, EPI_ISL_1514662, EPI_ISL_1514677, EPI_ISL_1514909, EPI_ISL_1515028, EPI_ISL_1515506			
see above	Laboratory Corporation of America	Centers for Disease Control and Prevention Division of Viral Diseases, Pathogen Discovery	Dakota Howard, Dhwani Batra, Peter W. Cook, Kara Moser, Adrian Paskey, Jason Caravas, Benjamin Rambo-Martin, Shatavia Morrison, Christopher Gulvick, Scott Sammons, Yvette Unoarumhi, Darlene Wagner, Matthew Schmerer, Minoo Agarwal, Eyad Almasri, Debbie Boles, Ayla Burns, Nuthawin Charoensri, Oren Cohen, Susan Countryman, Mary Ann Cristobal, Bobbi Croy, Suzanne Dale, Hrushikesh Deshmukh, Amanda Douglas, Vincent Drouillon, Marcia Eisenberg, Howard Engler, Rama Ghatti, Prashant Gupta, Susan Hicks, Jake Humphrey, Lax Iyer, Manoj Jain, Mohan Kolli, Brian Krueger, Tim Kuphal, Stanley Letovsky, Michael Levandoski, Craig Lukasik, Jonathan Meltzer, Brian Norvell, Mindy Nye, Scott Parker, Christos Petropoulos, John Pruitt, Steven Ragan, Scott Ryan, Mike Sapeta, Jana Schroth, Suresh Babu Selvaraju, Goran Stevovic, Amanda Suchanek, Andrea Throop, Lyndon Tilson, Thomas Urban, Joe Voshell, Kimberly Wagner, Jonathan Williams, Mary Williamson, Qian Zeng, Tricia Zwiefelhofer, Clinton R. Paden, Duncan MacCannell
EPI_ISL_1516177	NJ Public Health and Environmental Laboratories	Centers for Disease Control and Prevention Division of Viral Diseases, Pathogen Discovery	Mili Sheth, Sarah Nobles, Jasmine Padilla, Mark Burroughs, Shoshona Le, Katie Dillon, Peter Cook, Clinton R. Paden, Dhwani Batra, Krista Queen, Kristen Knipe, Dakota Howard, Yvette Unoarumhi, Darlene Wagner, Matthew Schmerer, Ben L. Rambo-Martin, Kristine Lacek, Sam Shepard, Alison Laufer Halpin, Dave Wentworth, Vivien Dugan, Suxiang Tong, Justin Lee
EPI_ISL_1516268	WVDHHR - Office of Laboratory Services	Centers for Disease Control and Prevention Division of Viral Diseases, Pathogen Discovery	Mili Sheth, Sarah Nobles, Jasmine Padilla, Mark Burroughs, Shoshona Le, Katie Dillon, Peter Cook, Clinton R. Paden, Dhwani Batra, Krista Queen, Kristen Knipe, Dakota Howard, Yvette Unoarumhi, Darlene Wagner, Matthew Schmerer, Ben L. Rambo-Martin, Kristine Lacek, Sam Shepard, Alison Laufer Halpin, Dave Wentworth, Vivien Dugan, Suxiang Tong, Justin Lee
EPI_ISL_1516353, EPI_ISL_1516356	KY State Public Health Lab	Centers for Disease Control and Prevention Division of Viral Diseases, Pathogen Discovery	Mili Sheth, Sarah Nobles, Jasmine Padilla, Mark Burroughs, Shoshona Le, Katie Dillon, Peter Cook, Clinton R. Paden, Dhwani Batra, Krista Queen, Kristen Knipe, Dakota Howard, Yvette Unoarumhi, Darlene Wagner, Matthew Schmerer, Ben L. Rambo-Martin, Kristine Lacek, Sam Shepard, Alison Laufer Halpin, Dave Wentworth, Vivien Dugan, Suxiang Tong, Justin Lee

EPI_ISL_1516459, EPI_ISL_1516462, EPI_ISL_1516515, EPI_ISL_1516571	Broad Institute Clinical Research Sequencing Platform	Infectious Disease Program, Broad Institute of Harvard and MIT	Siddle,K.J., Adams,G., Pearlman,L., Gladden-Young,A., Vicente,G., Blumenstiel,B., DeFelice,M., Lee,M., McGovern,S., Lagerborg,K., Rudy,M., DeRuff,K., Carter,A., Normandin,E., Bauer,M., Reilly,S., Tomkins-Tinch,C., Loreth,C., Chaluvadi,S., Meldrim,J., Granger,B., Lemieux,J.E., Birren,B.W., Sabeti,P.C., Larkin,K., Dodge,S., Lennon,N., Madoff,L., Brown,C., Gallagher,G., Smole,S., Park,D.J., Gabriel,S., and MacInnis,B.L.
EPI_ISL_1516604, EPI_ISL_1516629, EPI_ISL_1516639, EPI_ISL_1516640, EPI_ISL_1516658, EPI_ISL_1516664	Rhode Island Department of Health	Infectious Disease Program, Broad Institute of Harvard and MIT	Siddle,K.J., Azevedo,K., Miller,A., Adams,G., Pearlman,L., Gladden-Young,A., Lagerborg,K., Rudy,M., DeRuff,K., Carter,A., Normandin,E., Bauer,M., Reilly,S., Tomkins-Tinch,C., Loreth,C., Chaluvadi,S., Lemieux,J.E., Birren,B.W., Sabeti,P.C., Huard,R., King,E., Park,D.J., and MacInnis,B.L.
EPI_ISL_1516691, EPI_ISL_1516695, EPI_ISL_1516697, EPI_ISL_1516699, EPI_ISL_1516703, EPI_ISL_1516704, EPI_ISL_1516713, EPI_ISL_1516714, EPI_ISL_1516731, EPI_ISL_1516741	Broad Institute Clinical Research Sequencing Platform	Infectious Disease Program, Broad Institute of Harvard and MIT	Siddle,K.J., Adams,G., Pearlman,L., Gladden-Young,A., Vicente,G., Blumenstiel,B., DeFelice,M., Lee,M., McGovern,S., Lagerborg,K., Rudy,M., DeRuff,K., Carter,A., Normandin,E., Bauer,M., Reilly,S., Tomkins-Tinch,C., Loreth,C., Chaluvadi,S., Meldrim,J., Granger,B., Lemieux,J.E., Birren,B.W., Sabeti,P.C., Larkin,K., Dodge,S., Lennon,N., Madoff,L., Brown,C., Gallagher,G., Smole,S., Park,D.J., Gabriel,S., and MacInnis,B.L.
EPI_ISL_1522085, EPI_ISL_1522233	Dutch COVID-19 response team	National Institute for Public Health and the Environment (RIVM)	Adam Meijer, Harry Vennema, Dirk Eggink, Jeroen Cremer, Sharon van den Brink, Bas van der Veer, AnneMarie van den Brandt, Lisa Wijsman, Kim Freriks, Ryanne Jaarsma, Euníce Then, Jolienke Hardeman, Lynn Aarts, Sanne Bos, Melissa van Tuil, Robert Kohl, Linda van de Nes, Sjoerd Kuiling, James Groot, Florian Zwagemaker, Dennis Schmitz, Annelies Kroneman, Karim Hajji, Chantal Reusken, on behalf of the national COVID-19 response team
EPI_ISL_1523778	Maine Health and Environmental Testing Laboratory	Tewhey Lab, The Jackson Laboratory	Matluk,N., Dewey,H., Iosue,F., Barter,M., Lynch,R., Munger,H. and Tewhey,R.
EPI_ISL_1525032, EPI_ISL_1525136, EPI_ISL_1525202, EPI_ISL_1525213	Aegis Sciences Corporation	Centers for Disease Control and Prevention Division of Viral Diseases, Pathogen Discovery	Dakota Howard, Dhwani Batra, Peter W. Cook, Kara Moser, Adrian Paskey, Jason Caravas, Benjamin Rambo-Martin, Shatavia Morrison, Christopher Gulvick, Scott Sammons, Yvette Unoarumhi, Darlene Wagner, Matthew Schmerer, Cyndi Clark, Patrick Campbell, Rob Case, Vikramsinha Ghorpade, Holly Houdeshell, Ola Kvalvaag, Dillon Nall, Ethan Sanders, Alec Vest, Shaun Westlund, Matthew Hardison, Clinton R. Paden, Duncan MacCannell
EPI_ISL_1525374, EPI_ISL_1525379, EPI_ISL_1525386, EPI_ISL_1525472, EPI_ISL_1525684, EPI_ISL_1525805, EPI_ISL_1525823	Fulgent Genetics	Centers for Disease Control and Prevention Division of Viral Diseases, Pathogen Discovery	Dakota Howard, Dhwani Batra, Peter W. Cook, Kara Moser, Adrian Paskey, Jason Caravas, Benjamin Rambo-Martin, Shatavia Morrison, Christopher Gulvick, Scott Sammons, Yvette Unoarumhi, Darlene Wagner, Matthew Schmerer, Harry Gao, Mickey Li, John Gao, Joseph Fierro, Benafsh Sapra, Becky Tsai, Yan Meng, Doreen Ng, James Xie, Clinton R. Paden, Duncan MacCannell
EPI_ISL_1525936, EPI_ISL_1526011, EPI_ISL_1526036	Quest Diagnostics Incorporated	Centers for Disease Control and Prevention Division of Viral Diseases, Pathogen Discovery	Dakota Howard, Dhwani Batra, Peter W. Cook, Kara Moser, Adrian Paskey, Jason Caravas, Benjamin Rambo-Martin, Shatavia Morrison, Christopher Gulvick, Scott Sammons, Yvette Unoarumhi, Darlene Wagner, Matthew Schmerer, S. H. Rosenthal, A. Gerasimova, R. M. Kagan, B. Anderson, M. Hua, Y. Liu, L.E. Bernstein, K.E. Livingston, A. Perez, I. A. Shlyakhter, R. V. Rolando, R. Owen, P. Tanpaiboon, F. Lacbawan, Clinton R. Paden, Duncan MacCannell
EPI_ISL_1526517	CNR Virus des Infections Respiratoires - France SUD	CNR Virus des Infections Respiratoires - France SUD	Antonin Bal, Gregory Destras, Gwendolyne Burfin, Hadrien Regue, Quentin Semanas, Martine Valette, Bruno Lina, Laurence Josset
EPI_ISL_1526859, EPI_ISL_1526863, EPI_ISL_1526864, EPI_ISL_1526888, EPI_ISL_1526897, EPI_ISL_1526902, EPI_ISL_1526908	Virginia Division of Consolidated Laboratory Services	Virginia Division of Consolidated Laboratory Services	Virginia DCLS
EPI_ISL_1527047, EPI_ISL_1527051	University of Mississippi Medical Center, Department of Pathology	University of Mississippi Medical Center, Molecular and Genomics Core Facility	Ashley C. Johnson, Ithiel J. Frame, Krishna K. Ayyalasomayajula, Michael R. Garrett, D. Ashley Robinson
EPI_ISL_1527096, EPI_ISL_1527104, EPI_ISL_1527208, EPI_ISL_1527215	Massachusetts State Public Health Laboratory	Massachusetts State Public Health Laboratory	Andrew Lang, Timelia Fink, Glen Gallagher, Sandra Smole
EPI_ISL_1527243	University of Mississippi Medical Center, Department of Pathology	University of Mississippi Medical Center, Molecular and Genomics Core Facility	Ashley C. Johnson, Ithiel J. Frame, Krishna K. Ayyalasomayajula, Michael R. Garrett, D. Ashley Robinson
EPI_ISL_1527536	School of Pharmacy, Shenandoah University	School of Pharmacy, Shenandoah University	Adams,S.M., Harralson,A.F., Kidd,R.S., Sawyer,G.W.
EPI_ISL_1528048	Michigan Department of Health and Human Services, Bureau of Laboratories	Michigan Department of Health and Human Services, Bureau of Laboratories	Blankenship HM, Riner D, Soehnlen MK
EPI_ISL_1528096	Massachusetts State Public Health Laboratory	Massachusetts State Public Health Laboratory	Andrew Lang, Timelia Fink, Glen Gallagher, Sandra Smole
EPI_ISL_1528444	WVDHHR - Office of Laboratory Services	Centers for Disease Control and Prevention Division of Viral Diseases, Pathogen Discovery	Mili Sheth, Sarah Nobles, Jasmine Padilla, Mark Burroughs, Shoshona Le, Katie Dillon, Peter Cook, Clinton R. Paden, Dhwani Batra, Krista Queen, Kristen Knipe, Dakota Howard, Yvette Unoarumhi, Darlene Wagner, Matthew Schmerer, Ben L. Rambo-Wagner, Kristine Lacek, Sam Shepard, Alison Laufer Halpin, Dave Wentworth, Vivien Dugan, Suxiang Tong, Justin Lee
EPI_ISL_1528451, EPI_ISL_1528455, EPI_ISL_1528458	MD DOH Laboratories Administration	Centers for Disease Control and Prevention Division of Viral Diseases, Pathogen Discovery	Mili Sheth, Sarah Nobles, Jasmine Padilla, Mark Burroughs, Shoshona Le, Katie Dillon, Peter Cook, Clinton R. Paden, Dhwani Batra, Krista Queen, Kristen Knipe, Dakota Howard, Yvette Unoarumhi, Darlene Wagner, Matthew Schmerer, Ben L. Rambo-Martin, Kristine Lacek, Sam Shepard, Alison Laufer Halpin, Dave Wentworth, Vivien Dugan, Suxiang Tong, Justin Lee
EPI_ISL_1528464, EPI_ISL_1528474	RI State Health Laboratories	Centers for Disease Control and Prevention Division of Viral Diseases, Pathogen Discovery	Mili Sheth, Sarah Nobles, Jasmine Padilla, Mark Burroughs, Shoshona Le, Katie Dillon, Peter Cook, Clinton R. Paden, Dhwani Batra, Krista Queen, Kristen Knipe, Dakota Howard, Yvette Unoarumhi, Darlene Wagner, Matthew Schmerer, Ben L. Rambo-Martin, Kristine Lacek, Sam Shepard, Alison Laufer Halpin, Dave Wentworth, Vivien Dugan, Suxiang Tong, Justin Lee
EPI_ISL_1528506	"PA Department of Health, Bureau of Laboratories"	Centers for Disease Control and Prevention Division of Viral Diseases, Pathogen Discovery	Mili Sheth, Sarah Nobles, Jasmine Padilla, Mark Burroughs, Shoshona Le, Katie Dillon, Peter Cook, Clinton R. Paden, Dhwani Batra, Krista Queen, Kristen Knipe, Dakota Howard, Yvette Unoarumhi, Darlene Wagner, Matthew Schmerer, Ben L. Rambo-Martin, Kristine Lacek, Sam Shepard, Alison Laufer Halpin, Dave Wentworth, Vivien Dugan, Suxiang Tong, Justin Lee
EPI_ISL_1528529	GA Department of Public Health Laboratory	Centers for Disease Control and Prevention Division of Viral Diseases, Pathogen Discovery	Mili Sheth, Sarah Nobles, Jasmine Padilla, Mark Burroughs, Shoshona Le, Katie Dillon, Peter Cook, Clinton R. Paden, Dhwani Batra, Krista Queen, Kristen Knipe, Dakota Howard, Yvette Unoarumhi, Darlene Wagner, Matthew Schmerer, Ben L. Rambo-Martin, Kristine Lacek, Sam Shepard, Alison Laufer Halpin, Dave Wentworth, Vivien Dugan, Suxiang Tong, Justin Lee
EPI_ISL_1528554, EPI_ISL_1528561, EPI_ISL_1528568, EPI_ISL_1528571, EPI_ISL_1528577	WVU Rapid Development Lab	WVU and Marshall University Combined Genomics Core Facilities	"James Denvir, Peter Stoilov, Peter Perrotta, Wesley Kimble, Ryan Percifield"
EPI_ISL_1528579, EPI_ISL_1528591, EPI_ISL_1528746	Berkeley Medical Center	WVU and Marshall University Combined Genomics Core Facilities	"James Denvir, Peter Stoilov, Peter Perrotta, Wesley Kimble, Ryan Percifield"
EPI_ISL_1528888	4952 MacCorkle Ave SE, Charleston, WV 25304	WVU and Marshall University Combined Genomics Core Facilities	"James Denvir, Peter Stoilov, Peter Perrotta, Wesley Kimble, Ryan Percifield"
EPI_ISL_1530700	Sonora Quest Laboratories	TGen North	Jolene Bowers, Heather Centner, Chris French, Hayley Yaglom, Ashlyn Pfeiffer, Darrin Lemmer, Dave Engelthaler, The Arizona COVID Genomics Union (ACGU)
EPI_ISL_1530875, EPI_ISL_1530876	Genome Analysis Center, Kamma Memorial Hospital	Genome Analysis Center, Kamma Memorial Hospital	Hanako Yazawa, Jun Ishii, Satoko Soma, Kaori Watanabe, Takuya Yazawa, Michiaki Masuda, Hiroshi Kamma
EPI_ISL_1531013, EPI_ISL_1531017, EPI_ISL_1531078, EPI_ISL_1531079, EPI_ISL_1531083, EPI_ISL_1531085, EPI_ISL_1531090	Florida Bureau of Public Health Laboratories	Florida Bureau of Public Health Laboratories	Sarah Schmedes, Jason Blanton
EPI_ISL_1531741	School of Pharmacy, Shenandoah University	School of Pharmacy, Shenandoah University	Adams,S.M., Harralson,A.F., Kidd,R.S., Sawyer,G.W.
EPI_ISL_1533880	SYNLAB	GIGA Medical Genomics	Keith Durkin, Maria Artesi, Sébastien Bontems, Raphaël Boreux, Bouchra Boujemla, Nathalie Renotte, Cécile Meex, Pierrette Melin, Marie-Pierre Hayette,

			Vincent Bours
EPI_ISL_1534497, EPI_ISL_1534498, EPI_ISL_1534506	Ministry of Health Turkey	Ministry of Health Turkey	Fatma Bayrakdar, Yasemin Cosgun, Suleyman Yalcin, Gulay Korukluoglu
EPI_ISL_1540460	Virginia Division of Consolidated Laboratory Services	Virginia Division of Consolidated Laboratory Services	Virginia DCLS
EPI_ISL_1540737	The Jackson Laboratory	The Jackson Laboratory	Bergeron D, Renzette N, Adams M, Omerza G, Kelly K, Long J, Li L
EPI_ISL_1542144, EPI_ISL_1542363, EPI_ISL_1542386, EPI_ISL_1542390, EPI_ISL_1542533, EPI_ISL_1542538, EPI_ISL_1542725, EPI_ISL_1542760, EPI_ISL_1542778, EPI_ISL_1542782, EPI_ISL_1543140, EPI_ISL_1543156, EPI_ISL_1543664, EPI_ISL_1543753, EPI_ISL_1543830			
see above	Pandemic Response Lab - NYC	Pandemic Response Lab, R&D	Henry Lee, Michael Hammerling, Melissa Hopkins, Cybill del Castillo, Shinyoung Clair Kang, William Ward, Pradeep Bugga, Sol Rey, Dylan Law, Katharine Nelson, Haiping Hao, Jon Laurent
EPI_ISL_1547987, EPI_ISL_1548036	Johns Hopkins Hospital Department of Pathology	Johns Hopkins Hospital Department of Pathology	C. Paul Morris, Chun Huai Luo, Adannaya Amadi, Matthew Schwartz, Heba H. Mostafa
EPI_ISL_1553195	Illinois Department of Public Health - Springfield Lab	Illinois Department of Public Health - Springfield Lab	Bryan Sim, Gordon McCall
EPI_ISL_1558789	Pathology and Laboratory Medicine Institute, Cleveland Clinic, Ohio, USA	Pathology and Laboratory Medicine Institute, Cleveland Clinic, Ohio, USA	Joy Nakitandwe, Zheng Jin Tu, Jay Brock, Yu-Wei Cheng, Gary Procop, Daniel Rhoads, Daniel H. Farkas, David Bosler
EPI_ISL_1559127	Public Health Ontario Laboratory	Public Health Ontario Laboratory	Vanessa G Allen, Philip Banh, Yao Chen, Richard de Borja, Alireza Eshaghi, Nahuel Fittipaldi, Christine Frantz, Jonathan B Gubbay, Jennifer L Guthrie, Lawrence Heisler, Esha Joshi, Michael Laszloffy, Aimin Li, Michael CY Li, Dean Maxwell, Sandeep Nagra, Samir N Patel, Jared Simpson, Karthikeyan Sivaraman, Ashleigh Sullivan, Yogi Sundaravadanam, Sarah Teatero, Andre Villegas, Matthew Watson, Sandra Zittermann
EPI_ISL_1559501, EPI_ISL_1559564, EPI_ISL_1559578	Quest Diagnostics Incorporated	Centers for Disease Control and Prevention Division of Viral Diseases, Pathogen Discovery	Dakota Howard, Dhwani Batra, Peter W. Cook, Kara Moser, Adrian Paskey, Jason Caravas, Benjamin Rambo-Martin, Shatavia Morrison, Christopher Gulvick, Scott Sammons, Yvette Unoarumhi, Darlene Wagner, Matthew Schmerer, S. H. Rosenthal, A. Gerasimova, R. M. Kagan, B. Anderson, M. Hua, Y. Liu, L.E. Bernstein, K.E. Livingston, A. Perez, I. A. Shlyakhter, R. V. Rolando, R. Owen, P. Tanpaiboon, F. Lacbawan, Clinton R. Paden, Duncan MacCannell
EPI_ISL_1559653, EPI_ISL_1559801, EPI_ISL_1559924, EPI_ISL_1559926, EPI_ISL_1560049, EPI_ISL_1560077, EPI_ISL_1560256, EPI_ISL_1560257, EPI_ISL_1560259, EPI_ISL_1560316, EPI_ISL_1560354, EPI_ISL_1560368, EPI_ISL_1560370, EPI_ISL_1560412, EPI_ISL_1560413, EPI_ISL_1560468, EPI_ISL_1560532, EPI_ISL_1560638, EPI_ISL_1560658, EPI_ISL_1560703, EPI_ISL_1560704, EPI_ISL_1560722, EPI_ISL_1560764, EPI_ISL_1560790, EPI_ISL_1560832, EPI_ISL_1560870, EPI_ISL_1561218, EPI_ISL_1561228, EPI_ISL_1561230, EPI_ISL_1561321, EPI_ISL_1561740, EPI_ISL_1561883, EPI_ISL_1562114, EPI_ISL_1562243, EPI_ISL_1562248, EPI_ISL_1562258, EPI_ISL_1562264, EPI_ISL_1562280, EPI_ISL_1562403, EPI_ISL_1562487, EPI_ISL_1562497, EPI_ISL_1562568, EPI_ISL_1562583, EPI_ISL_1562584, EPI_ISL_1562585, EPI_ISL_1562635, EPI_ISL_1562646, EPI_ISL_1562695, EPI_ISL_1562797, EPI_ISL_1562878, EPI_ISL_1562901, EPI_ISL_1563084, EPI_ISL_1563114, EPI_ISL_1563133, EPI_ISL_1563134, EPI_ISL_1563166, EPI_ISL_1563244, EPI_ISL_1563357, EPI_ISL_1563404, EPI_ISL_1563406, EPI_ISL_1563546, EPI_ISL_1563580, EPI_ISL_1563592			
see above	Aegis Sciences Corporation	Centers for Disease Control and Prevention Division of Viral Diseases, Pathogen Discovery	Dakota Howard, Dhwani Batra, Peter W. Cook, Kara Moser, Adrian Paskey, Jason Caravas, Benjamin Rambo-Martin, Shatavia Morrison, Christopher Gulvick, Scott Sammons, Yvette Unoarumhi, Darlene Wagner, Matthew Schmerer, Cyndi Clark, Patrick Campbell, Rob Case, Vikramsinha Ghorpade, Holly Houdeshell, Ola Kvalvaag, Dillon Nall, Ethan Sanders, Alec Vest, Shaun Westlund, Matthew Hardison, Clinton R. Paden, Duncan MacCannell
EPI_ISL_1566399, EPI_ISL_1566429, EPI_ISL_1566437, EPI_ISL_1566450, EPI_ISL_1566514, EPI_ISL_1566530, EPI_ISL_1566541, EPI_ISL_1566562, EPI_ISL_1566565, EPI_ISL_1566570, EPI_ISL_1566594, EPI_ISL_1566609, EPI_ISL_1566625, EPI_ISL_1566647, EPI_ISL_1566654, EPI_ISL_1568245, EPI_ISL_1568304, EPI_ISL_1568322, EPI_ISL_1568369, EPI_ISL_1568374, EPI_ISL_1568413, EPI_ISL_1568415, EPI_ISL_1568483, EPI_ISL_1568489			
see above	SYNLAB MVZ Weiden	Robert Koch Institute	unknown
EPI_ISL_1568500	Synlab MVZ Augsburg	Robert Koch Institute	unknown
EPI_ISL_1568503	SYNLAB MVZ Weiden	Robert Koch Institute	unknown
EPI_ISL_1569738	Uniklinikum Regensburg; Institut fÃ¼r klinische Mikrobiologie und Hygiene	Robert Koch Institute	unknown
EPI_ISL_1571301, EPI_ISL_1571303, EPI_ISL_1571306, EPI_ISL_1571310, EPI_ISL_1571321, EPI_ISL_1571335, EPI_ISL_1571384, EPI_ISL_1571431, EPI_ISL_1571436, EPI_ISL_1571444	Eurofins LifeCodexx GmbH	Robert Koch Institute	unknown
EPI_ISL_1573100, EPI_ISL_1573107, EPI_ISL_1574446, EPI_ISL_1574469, EPI_ISL_1574483, EPI_ISL_1574556, EPI_ISL_1574572, EPI_ISL_1574664	SYNLAB MVZ Weiden	Robert Koch Institute	unknown
EPI_ISL_1575171	MD PHL	MD PHL	Maryland Department of Health Laboratories Administration
EPI_ISL_1575202	University of Texas, Genomic Sequencing and Analysis Facility	University of Texas, Genomic Sequencing and Analysis Facility	Jessica Podnar, Sylvie Beaudenon, Audrey Kelly, Zachary Carver, Anna Battenhouse, Andreas Matouschek
EPI_ISL_1575327	Pathology and Laboratory Medicine Institute, Cleveland Clinic, Ohio, USA	Pathology and Laboratory Medicine Institute, Cleveland Clinic, Ohio, USA	Joy Nakitandwe, Zheng Jin Tu, Jay Brock, Yu-Wei Cheng, Gary Procop, Daniel Rhoads, Daniel H. Farkas, David Bosler
EPI_ISL_1575487, EPI_ISL_1575555, EPI_ISL_1575703, EPI_ISL_1575867, EPI_ISL_1575896, EPI_ISL_1575937, EPI_ISL_1576107, EPI_ISL_1576166, EPI_ISL_1576202, EPI_ISL_1576215, EPI_ISL_1576248, EPI_ISL_1576255, EPI_ISL_1576257, EPI_ISL_1576392, EPI_ISL_1576503, EPI_ISL_1576515, EPI_ISL_1576518, EPI_ISL_1576556, EPI_ISL_1576688			
see above	Helix/Illumina	Centers for Disease Control and Prevention Division of Viral Diseases, Pathogen Discovery	Dakota Howard, Dhwani Batra, Peter W. Cook, Kara Moser, Adrian Paskey, Jason Caravas, Benjamin Rambo-Martin, Shatavia Morrison, Christopher Gulvick, Scott Sammons, Yvette Unoarumhi, Darlene Wagner, Matthew Schmerer, Eileen de Feo, Jan Antico, Christine Tran, Matthew Tolentino, Shannon Wickline, Kim Gietzen, Brad Sickler, Jingtao Liu, Eric Allen, Phil Febbo, Nicole L. Washington, Simon White, Geraint Levan, Kelly Schiabor Barrett, Elizabeth Cirulli, Alexandre Bolze, Ary Ascencio, Charlotte Rivera-Garcia, Ryan Cho, Jason Nguyen, Sherry Wang, Jimmy Ramirez, Tyler Cassens, Efren Sandoval, Magnus Isaksson, William Lee, David Becker, Marc Laurent, James Lu, Clinton R. Paden, Duncan MacCannell
EPI_ISL_1576837, EPI_ISL_1576841, EPI_ISL_1576842, EPI_ISL_1576843, EPI_ISL_1576844, EPI_ISL_1576845, EPI_ISL_1576846, EPI_ISL_1576847, EPI_ISL_1576848, EPI_ISL_1576849, EPI_ISL_1576850			
see above	Houston Health Dept.	Houston Health Dept.	Ryker Penn, Pamela Brown, Adolpho Lara, Yanlai Lai
EPI_ISL_1578114, EPI_ISL_1578115	Genome Analysis Center, Yamanashi Central Hospital	Genome Analysis Center, Yamanashi Central Hospital	Yosuke Hirotsu
EPI_ISL_1578149, EPI_ISL_1578164, EPI_ISL_1578189, EPI_ISL_1578193, EPI_ISL_1578209	Broad Institute Clinical Research Sequencing Platform	Infectious Disease Program, Broad Institute of Harvard and MIT	Siddle,K.J., Adams,G., Pearlman,L., Gladden-Young,A., Vicente,G., Blumenstiel,B., DeFelice,M., Lee,M., McGovern,S., Lagerborg,K., Rudy,M., DeRuff,K., Carter,A., Normandin,E., Bauer,M., Reilly,S., Tomkins-Tinch,C., Loreth,C., Chaluvadi,S., Meldrim,J., Granger,B., Lemieux,J.E., Birren,B.W., Sabeti,P.C., Larkin,K., Dodge,S., Lennon,N., Madoff,L., Brown,C., Gallagher,G., Smole,S., Park,D.J., Gabriel,S., and MacInnis,B.L.
EPI_ISL_1578214, EPI_ISL_1578337	Rhode Island Department of Health	Infectious Disease Program, Broad Institute of Harvard and MIT	Siddle,K.J., Azevedo,K., Miller,A., Adams,G., Pearlman,L., Gladden-Young,A., Lagerborg,K., Rudy,M., DeRuff,K., Carter,A., Normandin,E., Bauer,M., Reilly,S., Tomkins-Tinch,C., Loreth,C., Chaluvadi,S., Lemieux,J.E., Birren,B.W., Sabeti,P.C., Huard,R., King,E., Park,D.J., and MacInnis,B.L.
EPI_ISL_1578411, EPI_ISL_1578412, EPI_ISL_1578413	Broad Institute Clinical Research Sequencing Platform	Infectious Disease Program, Broad Institute of Harvard and MIT	Siddle,K.J., Adams,G., Pearlman,L., Gladden-Young,A., Vicente,G., Blumenstiel,B., DeFelice,M., Lee,M., McGovern,S., Lagerborg,K., Rudy,M., DeRuff,K., Carter,A., Normandin,E., Bauer,M., Reilly,S., Tomkins-Tinch,C., Loreth,C., Chaluvadi,S., Meldrim,J., Granger,B., Lemieux,J.E., Birren,B.W., Sabeti,P.C., Larkin,K., Dodge,S., Lennon,N., Madoff,L., Brown,C., Gallagher,G., Smole,S., Park,D.J., Gabriel,S., and MacInnis,B.L.
EPI_ISL_1578414	Rhode Island Department of Health	Infectious Disease Program, Broad Institute of Harvard and MIT	Siddle,K.J., Azevedo,K., Miller,A., Adams,G., Pearlman,L., Gladden-Young,A., Lagerborg,K., Rudy,M., DeRuff,K., Carter,A., Normandin,E., Bauer,M., Reilly,S., Tomkins-Tinch,C., Loreth,C., Chaluvadi,S., Lemieux,J.E., Birren,B.W., Sabeti,P.C., Huard,R., King,E., Park,D.J., and MacInnis,B.L.
EPI_ISL_1580358, EPI_ISL_1580361, EPI_ISL_1580362, EPI_ISL_1580368, EPI_ISL_1580409, EPI_ISL_1580436, EPI_ISL_1580437, EPI_ISL_1580439, EPI_ISL_1580442, EPI_ISL_1580446	University of Michigan Clinical Microbiology Laboratory	Lauring Lab, University of Michigan, Department of Microbiology and Immunology	Valesano

EPI_ISL_1580743, EPI_ISL_1580791, EPI_ISL_1580968, EPI_ISL_1581135, EPI_ISL_1581145, EPI_ISL_1581161, EPI_ISL_1581266, EPI_ISL_1581315, EPI_ISL_1581451, EPI_ISL_1581578, EPI_ISL_1581743, EPI_ISL_1581792			
see above	Helix/Illumina	Centers for Disease Control and Prevention Division of Viral Diseases, Pathogen Discovery	Dakota Howard, Dhwani Batra, Peter W. Cook, Kara Moser, Adrian Paskey, Jason Caravas, Benjamin Rambo-Martin, Shatavia Morrison, Christopher Gulvick, Scott Sammons, Yvette Unoarumhi, Darlene Wagner, Matthew Schmerer, Eileen de Feo, Jan Antico, Christine Tran, Matthew Tolentino, Shannon Wickline, Kim Gietzen, Brad Sickler, Jingtao Liu, Eric Allen, Phil Febbo, Nicole L. Washington, Simon White, Geraint Levan, Kelly Schiabor Barrett, Elizabeth Cirulli, Alexandre Bolze, Ary Ascencio, Charlotte Rivera-Garcia, Ryan Cho, Jason Nguyen, Sherry Wang, Jimmy Ramirez, Tyler Cassens, Efren Sandoval, Magnus Isaksson, William Lee, David Becker, Marc Laurent, James Lu, Clinton R. Paden, Duncan MacCannell
EPI_ISL_1581911, EPI_ISL_1582055, EPI_ISL_1582056, EPI_ISL_1582147	Quest Diagnostics Incorporated	Centers for Disease Control and Prevention Division of Viral Diseases, Pathogen Discovery	Dakota Howard, Dhwani Batra, Peter W. Cook, Kara Moser, Adrian Paskey, Jason Caravas, Benjamin Rambo-Martin, Shatavia Morrison, Christopher Gulvick, Scott Sammons, Yvette Unoarumhi, Darlene Wagner, Matthew Schmerer, S. H. Rosenthal, A. Gerasimova, R. M. Kagan, B. Anderson, M. Hua, Y. Liu, L.E. Bernstein, K.E. Livingston, A. Perez, I. A. Shlyakhter, R. V. Rolando, R. Owen, P. Tanpaiboon, F. Lacbawan, Clinton R. Paden, Duncan MacCannell
EPI_ISL_1582341, EPI_ISL_1582343, EPI_ISL_1582344	URMC LABS	Wadsworth Center, New York State Department of Health	Kirsten St. George, Daryl M. Lamson, Alexis Russell, Matthew Shudt, Melissa A Leisner, Jonathan Plitnick, Catharine Prussing, Navjot Singh, John Kelly, Erasmus Schneider, Erica Lasek-Nesselquist
EPI_ISL_1582627	University of Wisconsin-Madison AIDS Vaccine Research Laboratories	University of Wisconsin-Madison AIDS Vaccine Research Laboratories	Gage Moreno, Katarina Braun, et al. AIDS Vaccine Research Laboratories
EPI_ISL_1582765	Wyoming Public Health Laboratory	Wyoming Public Health Laboratory	Jim Mildenberger, Wanda Manley, Noah Hull, Taylor Fearing, Lynette Gumbleton, Channing Weber, Ashley Norberg, Chayse Rowley, Marley Goetz, Brian Dominguez, Elliot Thomasson, Cari Sloma, and Rob Christensen
EPI_ISL_1583295	Klinikum Wels-Grieskirchen	Bergthaler laboratory, CeMM Research Center for Molecular Medicine of the Austrian Academy of Sciences	Lukas Endler, Anna Schedl, Fabian Amman, Petr Triska, Thomas Penz, Benedikt Agerer, Maelle Le Moing, Michael Schuster, Bekir Erguner, Jan Laine, Martin Senekowitsch, Christoph Bock, Andreas Bergthaler
EPI_ISL_1583368, EPI_ISL_1583370, EPI_ISL_1583380, EPI_ISL_1583389, EPI_ISL_1583398	Austrian Agency for Health and Food Safety (AGES)	Bergthaler laboratory, CeMM Research Center for Molecular Medicine of the Austrian Academy of Sciences	Lukas Endler, Anna Schedl, Fabian Amman, Petr Triska, Thomas Penz, Benedikt Agerer, Maelle Le Moing, Michael Schuster, Bekir Erguner, Jan Laine, Martin Senekowitsch, Christoph Bock, Andreas Bergthaler
EPI_ISL_1587026	Altius Institute	Seattle Flu Study	Deborah A. Nickerson, Chris D. Frazar, Jover Lee, Benjamin Pelle, Erica Ryke, Matthew Richardson, Amanda Adler, Elisabeth Brandstetter, Peter D. Han, Kairsten Fay, Misja Ilcisin, Kirsten Lacombe, Thomas R. Sibley, Melissa Truong, Caitlin R. Wolf, Ryan Alexander, Daniel Bates, Rebecca Bruders, Stephanie DeBaun, Clem Green, Muhammad Halimun, Jessica Halow, Kneshay Harper, Matt Hartman, Andrew Meuser, Alex Nguyen, Truong Nguyen, Sofia Olsson, Sadie Patraw, Hannah Petersen, Tobias Ragoczy, Joshua Richards, Jacob Rodriguez, John Stamatoyannopoulos, Julia Wald, Olivia Waltner, Michael Boeckh, Janet A. Englund, Michael Famulare, Barry R. Lutz, Mark J. Rieder, Lea M. Starita, Matthew Thompson, Helen Y. Chu, Jay Shendure, Trevor Bedford
EPI_ISL_1587281, EPI_ISL_1587313, EPI_ISL_1587365	Yale Clinical Virology Lab	Grubaugh Lab - Yale School of Public Health	Joseph Fauver, Mallery Breban, Isabel Ott, Tara Alpert, Mary Petrone, Anderson Brito, Chantal Vogels, Annie Watkins, Chaney Kalinich, Jessica Rothman, Marie L. Landry, Nathan Grubaugh
EPI_ISL_1587580, EPI_ISL_1587581, EPI_ISL_1587582	Genome Analysis Center, Yamanashi Central Hospital	Genome Analysis Center, Yamanashi Central Hospital	Yosuke Hirotsu
EPI_ISL_1587785, EPI_ISL_1588079, EPI_ISL_1588389	Infinity Biologix	Centers for Disease Control and Prevention Division of Viral Diseases, Pathogen Discovery	Dakota Howard, Dhwani Batra, Peter W. Cook, Kara Moser, Adrian Paskey, Jason Caravas, Benjamin Rambo-Martin, Shatavia Morrison, Christopher Gulvick, Scott Sammons, Yvette Unoarumhi, Darlene Wagner, Matthew Schmerer, Christian Bixby, Yihe Wang, Jonathan Schultz, Chirayu Goswami, Russ Hager, Robin Grimwood, Clinton R. Paden, Duncan MacCannell
EPI_ISL_1591250, EPI_ISL_1591265	Caribbean Public Health Agency	Carrington Lab, Department of PreClinical Sciences, Faculty of Medical Sciences, The University of the West Indies	Nikita S. D. Sahadeo, Arianne Brown-Jordan, Sarah Hill, Vernie Ramkissoon, Roshan Parasram, Naresh Nandram, Avery Hinds, Jerome Foster, Stanley Giddings, Karla Georges, Marsha Ivey, Rahul Naidu, Risha Singh, SueMin Nathaniel, Rajini Haraksingh, Jaya Jayaraman, Chinna Chinnadurai, Adesh Ramsubhag, Nuno Faria, Oliver Pybus, Christopher Oura, Gabriel Escobar, Christine V. F. Carrington
EPI_ISL_1591350	Institute for Public Health of Zagreb County	Croatian Institute of Public Health	Irena Tabain, Ivana Ferenak
EPI_ISL_1591351, EPI_ISL_1591457, EPI_ISL_1591458, EPI_ISL_1591459, EPI_ISL_1591518, EPI_ISL_1591719	Caribbean Public Health Agency	Carrington Lab, Department of PreClinical Sciences, Faculty of Medical Sciences, The University of the West Indies	Nikita S. D. Sahadeo, Arianne Brown-Jordan, Sarah Hill, Vernie Ramkissoon, Roshan Parasram, Naresh Nandram, Avery Hinds, Jerome Foster, Stanley Giddings, Karla Georges, Marsha Ivey, Rahul Naidu, Risha Singh, SueMin Nathaniel, Rajini Haraksingh, Jaya Jayaraman, Chinna Chinnadurai, Adesh Ramsubhag, Nuno Faria, Oliver Pybus, Christopher Oura, Gabriel Escobar, Christine V. F. Carrington
EPI_ISL_1591720	Caribbean Public Health Laboratory	Carrington Lab, Department of PreClinical Sciences, Faculty of Medical Sciences, The University of the West Indies	Nikita S. D. Sahadeo, Arianne Brown-Jordan, Sarah Hill, Vernie Ramkissoon, Roshan Parasram, Naresh Nandram, Avery Hinds, Jerome Foster, Stanley Giddings, Karla Georges, Marsha Ivey, Rahul Naidu, Risha Singh, SueMin Nathaniel, Rajini Haraksingh, Jaya Jayaraman, Chinna Chinnadurai, Adesh Ramsubhag, Nuno Faria, Oliver Pybus, Christopher Oura, Gabriel Escobar, Christine V. F. Carrington
EPI_ISL_1591721, EPI_ISL_1591722	Caribbean Public Health Agency	Carrington Lab, Department of PreClinical Sciences, Faculty of Medical Sciences, The University of the West Indies	Nikita S. D. Sahadeo, Arianne Brown-Jordan, Sarah Hill, Vernie Ramkissoon, Roshan Parasram, Naresh Nandram, Avery Hinds, Jerome Foster, Stanley Giddings, Karla Georges, Marsha Ivey, Rahul Naidu, Risha Singh, SueMin Nathaniel, Rajini Haraksingh, Jaya Jayaraman, Chinna Chinnadurai, Adesh Ramsubhag, Nuno Faria, Oliver Pybus, Christopher Oura, Gabriel Escobar, Christine V. F. Carrington
EPI_ISL_1592545, EPI_ISL_1592600, EPI_ISL_1592643, EPI_ISL_1592708, EPI_ISL_1592918, EPI_ISL_1593077	Helix/Illumina	Centers for Disease Control and Prevention Division of Viral Diseases, Pathogen Discovery	Dakota Howard, Dhwani Batra, Peter W. Cook, Kara Moser, Adrian Paskey, Jason Caravas, Benjamin Rambo-Martin, Shatavia Morrison, Christopher Gulvick, Scott Sammons, Yvette Unoarumhi, Darlene Wagner, Matthew Schmerer, Eileen de Feo, Jan Antico, Christine Tran, Matthew Tolentino, Shannon Wickline, Kim Gietzen, Brad Sickler, Jingtao Liu, Eric Allen, Phil Febbo, Nicole L. Washington, Simon White, Geraint Levan, Kelly Schiabor Barrett, Elizabeth Cirulli, Alexandre Bolze, Ary Ascencio, Charlotte Rivera-Garcia, Ryan Cho, Jason Nguyen, Sherry Wang, Jimmy Ramirez, Tyler Cassens, Efren Sandoval, Magnus Isaksson, William Lee, David Becker, Marc Laurent, James Lu, Clinton R. Paden, Duncan MacCannell
EPI_ISL_1593731	DOHMH PHL	New York City Public Health Laboratory	Jade Wang, et al.
EPI_ISL_1593769	DOHMH Crown Heights	New York City Public Health Laboratory	Jade Wang, et al.
EPI_ISL_1593893	Arizona State Public Health Laboratory	Arizona State Public Health Laboratory	Trung Huynh, Jessica Escobar, Katherine Fullerton, Nobuko Fukushima, Stacy White, Linda Getsinger, Victor Waddell
EPI_ISL_1595589	Illinois Department of Public Health - Springfield Lab	Illinois Department of Public Health - Springfield Lab	Bryan Sim, Gordon McCall
EPI_ISL_1595846, EPI_ISL_1595847, EPI_ISL_1595848, EPI_ISL_1595850	Genome Analysis Center, Yamanashi Central Hospital	Genome Analysis Center, Yamanashi Central Hospital	Yosuke Hirotsu
EPI_ISL_1596003, EPI_ISL_1596918, EPI_ISL_1596940	Dutch COVID-19 response team	National Institute for Public Health and the Environment (RIVM)	Adam Meijer, Harry Vennema, Dirk Eggink, Jeroen Cremer, Sharon van den Brink, Bas van der Veer, AnneMarie van den Brandt, Lisa Wijsman, Kim Freriks, Ryanne Jaarsma, Euníce Then, Jolienke Hardeman, Lynn Aarts, Sanne Bos, Melissa van Tuil, Robert Kohl, Linda van de Nes, Sjoerd Kuiling, James Groot, Florian Zwagemaker, Dennis Schmitz, Annelies Kroneman, Karim Hajji, Chantal Reusken, on behalf of the national COVID-19 response team
EPI_ISL_1599372	Salzkammergutklinikum Vöcklabruck, Institut für Pathologie	Salzkammergutklinikum Vöcklabruck, Institut für Pathologie	Regina Stitz, Penka Lechner, Senka Rohregger, René Silye
EPI_ISL_1601445	UW Virology Lab	UW Virology Lab	Pavitra Roychoudhury, Hong Xie, Lasata Shrestha, Shah Mohamed Bakhash, Michelle Lin, Noah R. Baker, Sean Ellis, Saraswathi Sathees, Meei-Li Huang, Keith R Jerome, Alexander Greninger
EPI_ISL_1602420, EPI_ISL_1602421	Swedish national genomic surveillance program of SARS-CoV-2	The Public Health Agency of Sweden	Swedish national genomic surveillance program of SARS-CoV-2
EPI_ISL_1608582, EPI_ISL_1608588, EPI_ISL_1608589, EPI_ISL_1608590, EPI_ISL_1608591, EPI_ISL_1608593, EPI_ISL_1608594, EPI_ISL_1608595, EPI_ISL_1608598, EPI_ISL_1608599, EPI_ISL_1608605, EPI_ISL_1608625, EPI_ISL_1608636, EPI_ISL_1608647, EPI_ISL_1608653, EPI_ISL_1608731, EPI_ISL_1608742			
see above	Maryland Genomics, Institute for Genome Sciences, University of Maryland School of Medicine	Maryland Genomics, Institute for Genome Sciences, University of Maryland School of Medicine	Tallon, Luke J; Sadzewicz, Lisa D; Humphrys, Mike; Ott, Sandra; Roussey, Holly; Mehta, Aditya; Vavikolanu, Kranthi; Fraser, Claire M; Ravel, Jacques
EPI_ISL_1608874, EPI_ISL_1609190, EPI_ISL_1609462, EPI_ISL_1609470, EPI_ISL_1609535, EPI_ISL_1609874, EPI_ISL_1610008, EPI_ISL_1610344, EPI_ISL_1610660, EPI_ISL_1610676, EPI_ISL_1610790, EPI_ISL_1610901, EPI_ISL_1610916, EPI_ISL_1611038, EPI_ISL_1611086, EPI_ISL_1611532, EPI_ISL_1611738, EPI_ISL_1612000, EPI_ISL_1612534, EPI_ISL_1612629			

see above	Laboratory Corporation of America	Centers for Disease Control and Prevention Division of Viral Diseases, Pathogen Discovery	Dakota Howard, Dhwani Batra, Peter W. Cook, Kara Moser, Adrian Paskey, Jason Caravas, Benjamin Rambo-Martin, Shatavia Morrison, Christopher Gulvick, Scott Sammons, Yvette Unoarumhi, Darlene Wagner, Matthew Schmerer, Minoo Agarwal, Eyad Almasri, Debbie Boles, Ayla Burns, Nuthawin Charoensri, Oren Cohen, Susan Countryman, Mary Ann Cristobal, Bobbi Croy, Suzanne Dale, Hrushikesh Deshmukh, Amanda Douglas, Vincent Drouillon, Marcia Eisenberg, Howard Engler, Rama Ghatti, Prashant Gupta, Susan Hicks, Jake Humphrey, Lax Iyer, Manoj Jain, Mohan Kolli, Brian Krueger, Tim Kuphal, Stanley Letovsky, Michael Levandoski, Craig Lukasik, Jonathan Meltzer, Brian Norvell, Mindy Nye, Scott Parker, Christos Petropoulos, John Pruitt, Steven Ragan, Scott Ryan, Mike Sapeta, Jana Schroth, Suresh Babu Selvaraju, Goran Stevovic, Amanda Suchanek, Andrea Throop, Lyndon Tilson, Thomas Urban, Joe Voshell, Kimberly Wagner, Jonathan Williams, Mary Williamson, Qian Zeng, Tricia Zwiefelhofer, Clinton R. Paden, Duncan MacCannell
EPI_ISL_1612850, EPI_ISL_1613067, EPI_ISL_1614189, EPI_ISL_1614219, EPI_ISL_1614250, EPI_ISL_1614457, EPI_ISL_1614646, EPI_ISL_1614660	Fulgent Genetics	Centers for Disease Control and Prevention Division of Viral Diseases, Pathogen Discovery	Dakota Howard, Dhwani Batra, Peter W. Cook, Kara Moser, Adrian Paskey, Jason Caravas, Benjamin Rambo-Martin, Shatavia Morrison, Christopher Gulvick, Scott Sammons, Yvette Unoarumhi, Darlene Wagner, Matthew Schmerer, Harry Gao, Mickey Li, John Gao, Joseph Fierro, Benafsh Sapra, Becky Tsai, Yan Meng, Doreen Ng, James Xie, Clinton R. Paden, Duncan MacCannell
EPI_ISL_1614958, EPI_ISL_1614965, EPI_ISL_1614975, EPI_ISL_1614996	Quest Diagnostics Incorporated	Centers for Disease Control and Prevention Division of Viral Diseases, Pathogen Discovery	Dakota Howard, Dhwani Batra, Peter W. Cook, Kara Moser, Adrian Paskey, Jason Caravas, Benjamin Rambo-Martin, Shatavia Morrison, Christopher Gulvick, Scott Sammons, Yvette Unoarumhi, Darlene Wagner, Matthew Schmerer, S. H. Rosenthal, A. Gerasimova, R. M. Kagan, B. Anderson, M. Hua, Y. Liu, L.E. Bernstein, K.E. Livingston, A. Perez, I. A. Shlyakhter, R. V. Rolando, R. Owen, P. Tanpaiboon, F. Lacbawan, Clinton R. Paden, Duncan MacCannell
EPI_ISL_1615092, EPI_ISL_1615185, EPI_ISL_1615195, EPI_ISL_1615395	Helix/Illumina	Centers for Disease Control and Prevention Division of Viral Diseases, Pathogen Discovery	Dakota Howard, Dhwani Batra, Peter W. Cook, Kara Moser, Adrian Paskey, Jason Caravas, Benjamin Rambo-Martin, Shatavia Morrison, Christopher Gulvick, Scott Sammons, Yvette Unoarumhi, Darlene Wagner, Matthew Schmerer, Eileen de Feo, Jan Antico, Christine Tran, Matthew Tolentino, Shannon Wickline, Kim Gietzen, Brad Sickler, Jingtao Liu, Eric Allen, Phil Febbo, Nicole L. Washington, Simon White, Geraint Levan, Kelly Schiabor Barrett, Elizabeth Cirulli, Alexandre Bolze, Ary Ascencio, Charlotte Rivera-Garcia, Ryan Cho, Jason Nguyen, Sherry Wang, Jimmy Ramirez, Tyler Cassens, Efren Sandoval, Magnus Isaksson, William Lee, David Becker, Marc Laurent, James Lu, Clinton R. Paden, Duncan MacCannell
EPI_ISL_1616683	UW Virology Lab	UW Virology Lab	Pavitra Roychoudhury, Hong Xie, Lasata Shrestha, Shah Mohamed Bakhash, Michelle Lin, Noah R. Baker, Sean Ellis, Saraswathi Sathees, Meei-Li Huang, Keith R Jerome, Alexander Greninger
EPI_ISL_1617446, EPI_ISL_1617513, EPI_ISL_1617544, EPI_ISL_1617705, EPI_ISL_1617755, EPI_ISL_1617758, EPI_ISL_1617887, EPI_ISL_1618090, EPI_ISL_1618273, EPI_ISL_1618274, EPI_ISL_1618311, EPI_ISL_1618372, EPI_ISL_1618476, EPI_ISL_1618540			
see above	Swedish national genomic surveillance program of SARS-CoV-2	The Public Health Agency of Sweden	Swedish national genomic surveillance program of SARS-CoV-2
EPI_ISL_1620621	Puerto Rico Department of Health	Centers for Disease Control and Prevention, Dengue Branch	Gilberto A. Santiago, Glenda Gonzalez, Betzabel Flores, Keyla Charriez, Gabriela Paz-Bailey, Jorge L. Munoz-Jordan
EPI_ISL_1620658	GA Department of Public Health	GA Department of Public Health	Stacy Reeves, Jonathan Edwards, Cynthia Dixey, Tonia Parrott, Aliyah Fields, Taylor Smith
EPI_ISL_1620827	UW Virology Lab	UW Virology Lab	Pavitra Roychoudhury, Hong Xie, Lasata Shrestha, Shah Mohamed Bakhash, Michelle Lin, Noah R. Baker, Sean Ellis, Saraswathi Sathees, Meei-Li Huang, Keith R Jerome, Alexander Greninger
EPI_ISL_1621405, EPI_ISL_1621406, EPI_ISL_1621407, EPI_ISL_1621408, EPI_ISL_1621410, EPI_ISL_1621411, EPI_ISL_1621412, EPI_ISL_1621413, EPI_ISL_1621414, EPI_ISL_1621415, EPI_ISL_1621416, EPI_ISL_1621417, EPI_ISL_1621418, EPI_ISL_1621419, EPI_ISL_1621420			
see above	Hospital of the University of Pennsylvania Molecular Pathology Lab	Bushman Lab - University of Pennsylvania	John Everett, Kyle Rodino, Shantan Reddy, Pascha Hokama, Aoife M. Roche, Young Hwang, Abigail Glascock, Scott Sherrill-Mix, Samantha A. Whiteside, Jevon Graham-Wooten, Layla A. Khatib, Ayannah S. Fitzgerald, Arupa Ganguly, Mike Feldman, Brendan Kelly, Ronald G. Collman and Frederic Bushman
EPI_ISL_1621888, EPI_ISL_1622011	Quest Diagnostics Incorporated	Centers for Disease Control and Prevention Division of Viral Diseases, Pathogen Discovery	Dakota Howard, Dhwani Batra, Peter W. Cook, Kara Moser, Adrian Paskey, Jason Caravas, Benjamin Rambo-Martin, Shatavia Morrison, Christopher Gulvick, Scott Sammons, Yvette Unoarumhi, Darlene Wagner, Matthew Schmerer, S. H. Rosenthal, A. Gerasimova, R. M. Kagan, B. Anderson, M. Hua, Y. Liu, L.E. Bernstein, K.E. Livingston, A. Perez, I. A. Shlyakhter, R. V. Rolando, R. Owen, P. Tanpaiboon, F. Lacbawan, Clinton R. Paden, Duncan MacCannell
EPI_ISL_1622536, EPI_ISL_1622677, EPI_ISL_1622725, EPI_ISL_1622727	Servicio de Microbiología Clínica (Complejo Hospitalario de Navarra, Pamplona)	Centro de Secuenciación NASERTIC	Carmen Ezpeleta Baquedano, Ana Navascués, Ana Miqueleiz
EPI_ISL_1623807	Baylor Scott & White-Temple	Baylor Scott & White-Temple	Ari Rao, Linden Morales, Kimberly Walker, Marcus Volz, Shelby Hendrickson, Caitlin Maloney
EPI_ISL_1623958	MONTEFIORE MEDICAL CENTER LABORATORIES	Wadsworth Center, New York State Department of Health	Kirsten St. George, Daryl M. Lamson, Alexis Russell, Matthew Shudt, Melissa A Leisner, Jonathan Plitnick, Catharine Prussing, Navjot Singh, John Kelly, Erasmus Schneider, Erica Lasek-Nesselquist
EPI_ISL_1624108	Illinois Department of Public Health - Springfield Lab	Illinois Department of Public Health - Springfield Lab	Bryan Sim, Gordon McCall
EPI_ISL_1624226, EPI_ISL_1624230, EPI_ISL_1624231, EPI_ISL_1624232, EPI_ISL_1624233, EPI_ISL_1624234, EPI_ISL_1624235, EPI_ISL_1624236	Naval Health Clinic Annapolis	Naval Medical Research Center Biological Defense Research Directorate	Logan Voegtly, Catherine Arnold, Bishwo Adhikari, Francisco Malagon Bautista, Andrea Luquette, Gregory Rice, Andrew Bennett, Kyle Long, Lindsay Glang, Michael Deschenes, Regina Cer, Kimberly Bishop-Lilly
EPI_ISL_1624456	URMC LABS	Wadsworth Center, New York State Department of Health	Kirsten St. George, Daryl M. Lamson, Alexis Russell, Matthew Shudt, Melissa A Leisner, Jonathan Plitnick, Catharine Prussing, Navjot Singh, John Kelly, Erasmus Schneider, Erica Lasek-Nesselquist
EPI_ISL_1624483	SARATOGA HOSPITAL LABORATORY	Wadsworth Center, New York State Department of Health	Kirsten St. George, Daryl M. Lamson, Alexis Russell, Matthew Shudt, Melissa A Leisner, Jonathan Plitnick, Catharine Prussing, Navjot Singh, John Kelly, Erasmus Schneider, Erica Lasek-Nesselquist
EPI_ISL_1624500	Massachusetts State Public Health Laboratory	Massachusetts State Public Health Laboratory	Andrew Lang, Timelia Fink, Glen Gallagher, Sandra Smole
EPI_ISL_1624728, EPI_ISL_1624740	WVU Rapid Development Lab	WVU and Marshall University Combined Genomics Core Facilities	James Denvir, Peter Stoilov, Peter Perrotta, Wesley Kimble, Ryan Percifield
EPI_ISL_1624857, EPI_ISL_1624862, EPI_ISL_1624868, EPI_ISL_1624908, EPI_ISL_1624926, EPI_ISL_1624931, EPI_ISL_1624936, EPI_ISL_1624937, EPI_ISL_1624939	QLabs	WVU and Marshall University Combined Genomics Core Facilities	James Denvir, Peter Stoilov, Peter Perrotta, Wesley Kimble, Ryan Percifield
EPI_ISL_1626223	Wisconsin State Laboratory of Hygiene Communicable Disease Division	Wisconsin State Laboratory of Hygiene Communicable Disease Division	Kelsey R. Florek, Abigail C. Shockey
EPI_ISL_1626578	IN State Department of Health Laboratory Services	IN State Department of Health Laboratory Services	Cassandra Campion, Jamie Yeadon, Brian Pope, Lixia Liu, Kyle Brownlee, Melissa Hindenlang, Ankita Kashikar, Mark Glazier
EPI_ISL_1626749	Institute of Virology, Biomedical Research Center of the Slovak Academy of Sciences, Bratislava	Faculty of Natural Sciences, Comenius University, Bratislava	Viktoria Cabanova, Kristina Borsova, Brona Brejova, Viktoria Hodorova, Sabina Fumacova Havlikova, Juraj Kopacek, Martina Lickova, Lubomira Lukacikova, Martina Nebohacova, Monika Slavikova, Tomas Vinar, Jozef Nosek, Boris Klempa
EPI_ISL_1628207	UW Virology Lab	UW Virology Lab	Pavitra Roychoudhury, Hong Xie, Lasata Shrestha, Shah Mohamed Bakhash, Michelle Lin, Noah R. Baker, Sean Ellis, Meei-Li Huang, Keith R Jerome, Alexander Greninger
EPI_ISL_1628497, EPI_ISL_1628498	University of Mississippi Medical Center, Department of Pathology	University of Mississippi Medical Center, Molecular and Genomics Core Facility	Ashley C. Johnson, Ithiel J. Frame, Krishna K. Ayyalasomayajula, Michael R. Garrett, D. Ashley Robinson
EPI_ISL_1632544, EPI_ISL_1632545, EPI_ISL_1632547, EPI_ISL_1632595, EPI_ISL_1632603, EPI_ISL_1632606, EPI_ISL_1632608, EPI_ISL_1632610, EPI_ISL_1632616	Virginia Division of Consolidated Laboratory Services	Virginia Division of Consolidated Laboratory Services	Virginia DCLS

EPI_ISL_1633438, EPI_ISL_1633439, EPI_ISL_1633440, EPI_ISL_1633441, EPI_ISL_1633442, EPI_ISL_1633443, EPI_ISL_1633446, EPI_ISL_1633449, EPI_ISL_1633450, EPI_ISL_1633453, EPI_ISL_1633454, EPI_ISL_1633455, EPI_ISL_1633459, EPI_ISL_1633461			
see above	Tokyo Metropolitan Institute of Public Health	Tokyo Metropolitan Institute of Public Health	Takako Yamazak, Masaki Hayashi, Sanae Tomizawa, Yukino Segawa, Kenji Iida, Eri Satou, Naoko Sakai, Keiko Iwakoshi, Mami Nagashima,Takushi Fujiwara, Takashi Chiba, Kenji Sadamasu
EPI_ISL_1634165, EPI_ISL_1636043	Pandemic Response Lab - NYC	Pandemic Response Lab, R&D	Henry Lee, Michael Hammerling, Melissa Hopkins, Cybill del Castillo, Shinyoung Clair Kang, William Ward, Pradeep Bugga, Sol Rey, Dylan Law, Katharine Nelson, Haiping Hao, Jon Laurent
EPI_ISL_1638523	MVZ Dr. Eberhard & Partner Dortmund	Robert Koch Institute	unknown
EPI_ISL_1639270	Labor KneiÃŸler GmbH & Co. KG	Robert Koch Institute	unknown
EPI_ISL_1640720	MVZ Labor Krone GbR	Robert Koch Institute	unknown
EPI_ISL_1641632, EPI_ISL_1641684, EPI_ISL_1641782	SYNLAB MVZ Weiden	Robert Koch Institute	unknown
EPI_ISL_1643909, EPI_ISL_1643916, EPI_ISL_1643917, EPI_ISL_1643938	Eurofins LifeCodexx GmbH	Robert Koch Institute	unknown
EPI_ISL_1648091	NJDOH, Public Health and Environmental Laboratories	NJ_PHEL	Lindsey Bodnar, Shiv K. Verma, Jacquelyn Deverell, Matthew Scarnati, Dana Woell, Byeong Jeong
EPI_ISL_1648520, EPI_ISL_1648584, EPI_ISL_1648590, EPI_ISL_1648798, EPI_ISL_1648857, EPI_ISL_1648899, EPI_ISL_1648916	Quest Diagnostics Incorporated	Centers for Disease Control and Prevention Division of Viral Diseases, Pathogen Discovery	Dakota Howard, Dhwani Batra, Peter W. Cook, Kara Moser, Adrian Paskey, Jason Caravas, Benjamin Rambo-Martin, Shatavia Morrison, Christopher Gulvick, Scott Sammons, Yvette Unoarumhi, Darlene Wagner, Matthew Schmerer, S. H. Rosenthal, A. Gerasimova, R. M. Kagan, B. Anderson, M. Hua, Y. Liu, L.E. Bernstein, K.E. Livingston, A. Perez, I. A. Shlyakhter, R. V. Rolando, R. Owen, P. Tanpaiboon, F. Lacbawan, Clinton R. Paden, Duncan MacCannell
EPI_ISL_1649000, EPI_ISL_1649002, EPI_ISL_1649083, EPI_ISL_1649113, EPI_ISL_1649151, EPI_ISL_1649152, EPI_ISL_1649183, EPI_ISL_1649203, EPI_ISL_1649284, EPI_ISL_1649366, EPI_ISL_1649404, EPI_ISL_1649418, EPI_ISL_1649460, EPI_ISL_1649500, EPI_ISL_1649501, EPI_ISL_1649520, EPI_ISL_1649528, EPI_ISL_1649565, EPI_ISL_1649567, EPI_ISL_1649763, EPI_ISL_1649848, EPI_ISL_1649919, EPI_ISL_1649942, EPI_ISL_1649965, EPI_ISL_1649975, EPI_ISL_1650080, EPI_ISL_1650112, EPI_ISL_1650239, EPI_ISL_1650280, EPI_ISL_1650525, EPI_ISL_1650545, EPI_ISL_1650562, EPI_ISL_1650582, EPI_ISL_1650737, EPI_ISL_1650983			
see above	Aegis Sciences Corporation	Centers for Disease Control and Prevention Division of Viral Diseases, Pathogen Discovery	Dakota Howard, Dhwani Batra, Peter W. Cook, Kara Moser, Adrian Paskey, Jason Caravas, Benjamin Rambo-Martin, Shatavia Morrison, Christopher Gulvick, Scott Sammons, Yvette Unoarumhi, Darlene Wagner, Matthew Schmerer, Cyndi Clark, Patrick Campbell, Rob Case, Vikramsinha Ghorpade, Holly Houdeshell, Ola Kvalvaag, Dillon Nall, Ethan Sanders, Alec Vest, Shaun Westlund, Matthew Hardison, Clinton R. Paden, Duncan MacCannell
EPI_ISL_1651301, EPI_ISL_1651304	GA Department of Public Health	GA Department of Public Health	Stacy Reeves, Jonathan Edwards, Cynthia Dixey, Tonia Parrott, Aliyah Fields, Taylor Smith
EPI_ISL_1651343, EPI_ISL_1651430, EPI_ISL_1651501, EPI_ISL_1651510	Florida Bureau of Public Health Laboratories	Florida Bureau of Public Health Laboratories	Sarah Schmedes, Jason Blanton
EPI_ISL_1651615	University of Michigan Clinical Microbiology Laboratory	Lauring Lab, University of Michigan, Department of Microbiology and Immunology	Valesano
EPI_ISL_1652488	Illinois Department of Public Health	Gagnon Lab, Southern Illinois University	Keith Gagnon
EPI_ISL_1654604	NYU Langone Health	Departments of Pathology and Medicine, New York University School of Medicine	Adriana Heguy, Dacia Dimartino, Emily Guzman, Christian Marier, Peter Meyn, Sitharam Ramaswami, Gael Westby, Paul Zappile, Yutong Zhang, Paolo Cotzia, Guiqing Wang
EPI_ISL_1655075	Fujian Center for Disease Control and Prevention	Fujian Center for Disease Control and Prevention	Lin Qi, Huang Zhimiao, Weng Yuwei
EPI_ISL_1656749, EPI_ISL_1656750, EPI_ISL_1656753, EPI_ISL_1656754, EPI_ISL_1656757, EPI_ISL_1656758, EPI_ISL_1656759, EPI_ISL_1656763, EPI_ISL_1656764, EPI_ISL_1656765, EPI_ISL_1656766, EPI_ISL_1656768, EPI_ISL_1656770, EPI_ISL_1656771, EPI_ISL_1656774, EPI_ISL_1656775, EPI_ISL_1656778, EPI_ISL_1656779, EPI_ISL_1656780, EPI_ISL_1656784, EPI_ISL_1656785, EPI_ISL_1656786, EPI_ISL_1656787, EPI_ISL_1656789			
see above	Tokyo Metropolitan Institute of Public Health	Tokyo Metropolitan Institute of Public Health	Masaki Hayashi, Takako Yamazaki,Yumi Ooba , Tomoki Igarashi , Yoshihiro Ohsawa ,Takushi Fujiwara, Hirofumi Miyake, Mami Nagashima, Jun Suzuki, Kenji Sadamasu
EPI_ISL_1660080, EPI_ISL_1660117	Swedish national genomic surveillance program of SARS-CoV-2	The Public Health Agency of Sweden	Maximilian Riess, Maria Lind Karlberg, Alma Brolund, Swedish national genomic surveillance program of SARS-CoV-2
EPI_ISL_1660765, EPI_ISL_1660766, EPI_ISL_1660767	University of Liège COVID-19 testing center	GIGA Medical Genomics	Keith Durkin, Maria Artesi, Sébastien Bontems, Raphaël Boreux, Bouchra Boujemla, Nathalie Renotte, Cécile Meex, Pierrette Melin, Marie-Pierre Hayette, Vincent Bours
EPI_ISL_1662673	Delaware Public Health Lab	Delaware Public Health Lab	Rebecca Savage
EPI_ISL_1662770, EPI_ISL_1662802, EPI_ISL_1662829, EPI_ISL_1662884, EPI_ISL_1662892, EPI_ISL_1662998	Johns Hopkins Hospital Department of Pathology	Johns Hopkins Hospital Department of Pathology	C. Paul Morris, Chun Huai Luo, Adannaya Amadi, Matthew Schwartz, Heba H. Mostafa
EPI_ISL_1663126	Illinois Department of Public Health	Illinois Department of Public Health - Chicago Lab	Vineet K. Dhiman, Ira Heimler
EPI_ISL_1664390, EPI_ISL_1664394	Clinical Molecular Microbiology Laboratory, UNC Hospitals	Jeremy Wang	Jeremy Wang, Alexander Rubinsteyn, Colleen Rice, Jason Smedberg, Shawn Hawken, Melissa Miller, Corbin Jones, Robert Hagan
EPI_ISL_1666078, EPI_ISL_1666146, EPI_ISL_1666147, EPI_ISL_1666417, EPI_ISL_1666419, EPI_ISL_1666477, EPI_ISL_1667091, EPI_ISL_1667309	Fulgent Genetics	Centers for Disease Control and Prevention Division of Viral Diseases, Pathogen Discovery	Dakota Howard, Dhwani Batra, Peter W. Cook, Kara Moser, Adrian Paskey, Jason Caravas, Benjamin Rambo-Martin, Shatavia Morrison, Christopher Gulvick, Scott Sammons, Yvette Unoarumhi, Darlene Wagner, Matthew Schmerer, Harry Gao, Mickey Li, John Gao, Joseph Fierro, Benafsh Sapra, Becky Tsai, Yan Meng, Doreen Ng, James Xie, Clinton R. Paden, Duncan MacCannell
EPI_ISL_1667809, EPI_ISL_1667927, EPI_ISL_1668028, EPI_ISL_1668333	Aegis Sciences Corporation	Centers for Disease Control and Prevention Division of Viral Diseases, Pathogen Discovery	Dakota Howard, Dhwani Batra, Peter W. Cook, Kara Moser, Adrian Paskey, Jason Caravas, Benjamin Rambo-Martin, Shatavia Morrison, Christopher Gulvick, Scott Sammons, Yvette Unoarumhi, Darlene Wagner, Matthew Schmerer, Cyndi Clark, Patrick Campbell, Rob Case, Vikramsinha Ghorpade, Holly Houdeshell, Ola Kvalvaag, Dillon Nall, Ethan Sanders, Alec Vest, Shaun Westlund, Matthew Hardison, Clinton R. Paden, Duncan MacCannell
EPI_ISL_1668465, EPI_ISL_1668636, EPI_ISL_1668934, EPI_ISL_1669190	Quest Diagnostics Incorporated	Centers for Disease Control and Prevention Division of Viral Diseases, Pathogen Discovery	Dakota Howard, Dhwani Batra, Peter W. Cook, Kara Moser, Adrian Paskey, Jason Caravas, Benjamin Rambo-Martin, Shatavia Morrison, Christopher Gulvick, Scott Sammons, Yvette Unoarumhi, Darlene Wagner, Matthew Schmerer, S. H. Rosenthal, A. Gerasimova, R. M. Kagan, B. Anderson, M. Hua, Y. Liu, L.E. Bernstein, K.E. Livingston, A. Perez, I. A. Shlyakhter, R. V. Rolando, R. Owen, P. Tanpaiboon, F. Lacbawan, Clinton R. Paden, Duncan MacCannell
EPI_ISL_1669761, EPI_ISL_1669762, EPI_ISL_1669764	Genome Analysis Center, Yamanashi Central Hospital	Genome Analysis Center, Yamanashi Central Hospital	Yosuke Hirotsu
EPI_ISL_1671116, EPI_ISL_1671117, EPI_ISL_1671118, EPI_ISL_1671119	Institute for Laboratory Diagnostics and Microbiology, Klinikum Klagenfurt am Wörthersee	Bergthaler laboratory, CeMM Research Center for Molecular Medicine of the Austrian Academy of Sciences	Lukas Endler, Anna Schedl, Fabian Amman, Petr Triska, Thomas Penz, Benedikt Agerer, Maelle Le Moing, Michael Schuster, Bekir Erguner, Jan Laine, Martin Senekowitsch, Christoph Bock, Andreas Bergthaler
EPI_ISL_1671352, EPI_ISL_1671353	TEMPUS LABS INC	Wadsworth Center, New York State Department of Health	Kirsten St. George, Daryl M. Lamson, Alexis Russell, Matthew Shudt, Melissa A Leisner, Jonathan Plitnick, Catharine Prussing, Navjot Singh, John Kelly, Erasmus Schneider, Erica Lasek-Nesselquist
EPI_ISL_1671354	ALBANY MEDICAL CENTER	Wadsworth Center, New York State Department of Health	Kirsten St. George, Daryl M. Lamson, Alexis Russell, Matthew Shudt, Melissa A Leisner, Jonathan Plitnick, Catharine Prussing, Navjot Singh, John Kelly, Erasmus Schneider, Erica Lasek-Nesselquist
EPI_ISL_1671512, EPI_ISL_1671513	SUNY UPSTATE MEDICAL UNIVERSITY	Wadsworth Center, New York State Department of Health	Kirsten St. George, Daryl M. Lamson, Alexis Russell, Matthew Shudt, Melissa A Leisner, Jonathan Plitnick, Catharine Prussing, Navjot Singh, John Kelly, Erasmus Schneider, Erica Lasek-Nesselquist
EPI_ISL_1672267	SYNLAB	GIGA Medical Genomics	Keith Durkin, Maria Artesi, Sébastien Bontems, Raphaël Boreux, Bouchra Boujemla, Nathalie Renotte, Cécile Meex, Pierrette Melin, Marie-Pierre Hayette, Vincent Bours
EPI_ISL_1672287	University of Liège COVID-19 testing center	GIGA Medical Genomics	Keith Durkin, Maria Artesi, Sébastien Bontems, Raphaël Boreux, Bouchra Boujemla, Nathalie Renotte, Cécile Meex, Pierrette Melin, Marie-Pierre Hayette,

			Vincent Bours
EPI_ISL_1672288	SYNLAB	GIGA Medical Genomics	Keith Durkin, Maria Artesi, Sébastien Bontems, Raphaël Boreux, Bouchra Boujemla, Nathalie Renotte, Cécile Meex, Pierrette Melin, Marie-Pierre Hayette, Vincent Bours
EPI_ISL_1672383	MD PHL	MD PHL	Maryland Department of Health Laboratories Administration
EPI_ISL_675164	Lighthouse Lab in Alderley Park	Wellcome Sanger Institute for the COVID-19 Genomics UK (COG-UK) Consortium	Jacquelyn Wynn, Mairead Hyland, The Lighthouse Lab in Alderley Park and Alex Alderton, Roberto Amato, Sonia Goncalves, Ewan Harrison, David K. Jackson, Ian Johnston, Dominic Kwiatkowski, Cordelia Langford, John Sillitoe on behalf of the Wellcome Sanger Institute COVID-19 Surveillance Team
EPI_ISL_736897	Pathogen Genomics Center, National Institute of Infectious Diseases	Pathogen Genomics Center, National Institute of Infectious Diseases	Tsuyoshi Sekizuka, Kentaro Itokawa, Rina Tanaka, Masanori Hashino, Makoto Kuroda
EPI_ISL_791161, EPI_ISL_791177	University of Wisconsin-Madison AIDS Vaccine Research Laboratories	University of Wisconsin-Madison AIDS Vaccine Research Laboratories	Gage Moreno, Katarina Braun, et al. AIDS Vaccine Research Laboratories
EPI_ISL_791346	Johns Hopkins Hospital Department of Pathology	Johns Hopkins Hospital Department of Pathology	C. Paul Morris, Chun Huai Luo, Adannaya Amadi, Nicholas Gallagher, Heba H. Mostafa
EPI_ISL_806807, EPI_ISL_806808	Gundersen Molecular Diagnostics Laboratory	Kabara Cancer Research Institute	Craig S. Richmond, Paraic A. Kenny
EPI_ISL_831486	University of Wisconsin-Madison AIDS Vaccine Research Laboratories	University of Wisconsin-Madison AIDS Vaccine Research Laboratories	Gage Moreno, Katarina Braun, et al. AIDS Vaccine Research Laboratories
EPI_ISL_860044	BTC, Khalifa University	BTC, Khalifa University	Al Safar et al
EPI_ISL_861474	Gundersen Molecular Diagnostics Laboratory	Kabara Cancer Research Institute	Craig S. Richmond, Paraic A. Kenny
EPI_ISL_862036, EPI_ISL_862037, EPI_ISL_862038	Johns Hopkins Hospital Department of Pathology	Johns Hopkins Hospital Department of Pathology	C. Paul Morris, Chun Huai Luo, Adannaya Amadi, Matthew Schwartz, Nicholas Gallagher, Heba H. Mostafa
EPI_ISL_862670	New Mexico Department of Health Scientific Laboratory	New Mexico Department of Health Scientific Laboratory	D'eldra Malone, Ellie Johnson, Anastacia Griego-Fisher
EPI_ISL_876148	Massachusetts State Public Health Laboratory	Massachusetts State Public Health Laboratory	Andrew Lang, Timelia Fink, Glen Gallagher, Sandra Smole
EPI_ISL_876662	Helix/Illumina	Genomics and Discovery, Respiratory Viruses Branch, Division of Viral Diseases, Centers for Disease Control and Prevention	Peter W. Cook,Dhwani Batra,Ben L. Rambo-Martin,Eileen de Feo,Jan Antico,Christine Tran,Matthew Tolentino,Shannon Wickline,Kim Gietzen,Brad Sickler,Jingtao Liu,Eric Allen,Phil Febbo,Summer Galloway,Nicole L. Washington,Simon White,Geraint Levan,Kelly Schiabor Barrett,Elizabeth Cirulli,Alexandre Bolze,Ary Ascencio,Charlotte Rivera-Garcia,Ryan Cho,Jason Nguyen,Sherry Wang,Jimmy Ramirez,Tyler Cassens,Efren Sandoval,Magnus Isaksson,William Lee,David Becker,Marc Laurent,James Lu,Clinton R. Paden,Suxiang Tong,Duncan MacCannell,
EPI_ISL_883003	Maryland Public Health Laboratory	Maryland Public Health Laboratory	Maryland Department of Health Laboratories Administration
EPI_ISL_883314	DOHMH Jamaica	New York City Public Health Laboratory	Jade Wang, et al.
EPI_ISL_883348	DOHMH Corona	New York City Public Health Laboratory	Jade Wang, et al.
EPI_ISL_886790, EPI_ISL_886810, EPI_ISL_887695, EPI_ISL_887901, EPI_ISL_887902, EPI_ISL_887903, EPI_ISL_887904	Labcorp	Genomics and Discovery, Respiratory Viruses Branch, Division of Viral Diseases, Centers for Disease Control and Prevention	Peter W. Cook,Dhwani Batra,Ben L. Rambo-Martin,Summer Galloway,Brian Krueger,Minoo Agarwal,Eyad Almasri,Debbie Boles,Ayla Burns,Nuthawin Charoensri,Oren Cohen,Susan Countryman,Mary Ann Cristobal,Bobbi Croy,Suzanne Dale,Hrushikesh Deshmukh,Amanda Douglas,Vincent Drouillon,Marcia Eisenberg,Howard Engler,Rama Ghatti,Prashant Gupta,Susan Hicks,Jake Humphrey,Lax Iyer,Manoj Jain,Mohan Kolli,Tim Kuphal,Stanley Letovsky,Michael Levandoski,Craig Lukasik,Jonathan Meltzer,Brian Norvell,Mindy Nye,Scott Parker,Christos Petropoulos,John Pruitt,Steven Ragan,Scott Ryan,Mike Sapeta,Jana Schroth,Suresh Babu Selvaraju,Goran Stevovic,Amanda Suchanek,Andrea Throop,Lyndon Tilson,Thomas Urban,Joe Voshell,Kimberly Wagner,Jonathan Williams,Mary Williamson,Qian Zeng,Tricia Zwiefelhofer,Clinton R. Paden,Suxiang Tong,Duncan MacCannell,
EPI_ISL_888669	University of Michigan Clinical Microbiology Laboratory	Lauring Lab, University of Michigan, Department of Microbiology and Immunology	Valesano
EPI_ISL_890293	KU Leuven, Rega Institute, Clinical and Epidemiological Virology	KU Leuven, Rega Institute, Clinical and Epidemiological Virology	Tony Wawina-Bokalanga, Bert Vanmechelen, Joan Marti-Carerras, Piet Maes
EPI_ISL_895012, EPI_ISL_895013	Niigata Prefectural Institute of Public Health and Environmental Sciences	Pathogen Genomics Center, National Institute of Infectious Diseases	Tsuyoshi Sekizuka, Kentaro Itokawa, Rina Tanaka, Masanori Hashino, Makoto Kuroda
EPI_ISL_902908	Department of Virology and Immunology, University of Helsinki and Helsinki University Hospital, Huslab Finland	Department of Virology, Faculty of Medicine, University of Helsinki, Helsinki, Finland	Teemu Smura, Ravi Kant, Phuoc Truong, Hussein Alburkat, Hannimari Kallio-Kokko, Jenni Virtanen, Maija Suvanto, Essi Korhonen, Sari Hannula, Harri Kangas, Hanna Liimatainen, Satu Kurkela, Hanna Jarva, Maija Lappalainen, Pekka Ellonen, Olli Vapalahti
EPI_ISL_905303	Dutch COVID-19 response team	National Institute for Public Health and the Environment (RIVM)	Adam Meijer, Harry Vennema, Dirk Eggink, Jeroen Cremer, Sharon van den Brink, Bas van der Veer, AnneMarie van den Brandt, Florian Zwagemaker, Dennis Schmitz, Chantal Reusken, on behalf of the national COVID-19 response team
EPI_ISL_905952, EPI_ISL_906030	OHSU Lab Services Molecular Microbiology Lab	Oregon SARS-CoV-2 Genome Sequencing Center	Brendan L. O'Connell, Sally Grindstaff, Kayla Carter, Ruth V. Nichols, Alec J. Hirsch, Donna Hansel, Guang Fan, Xuan, Qin, Daniel N. Streblow, William B. Messer, Andrew C. Adey, Benjamin N. Bimber, Brian J. O'Roak
EPI_ISL_911760	Johns Hopkins Hospital Department of Pathology	Johns Hopkins Hospital Department of Pathology	C. Paul Morris, Chun Huai Luo, Adannaya Amadi, Matthew Schwartz, Nicholas Gallagher, Heba H. Mostafa
EPI_ISL_915343	Quest Diagnostics	Quest Diagnostics	Rosenthal,S.H., Gerasimova,A., Kagan,R.M., Anderson, B., Hua, M., Liu Y., Bernstein, L.E., Livingston, K.E., Perez, A., Shalhout, D.F., Shlyakhter, I.A., Owen, R., Tanpaiboon, P., Lacbawan, F.
EPI_ISL_925079	TXDSHS	TXDSHS	Bonnie Oh, Anita Pokharel, James Daniel Bonser, Myong Koag, Chung Wang, Rachel Lee, Grace Kubin, Rashmi Tuladhar, Mayela Pedrueza, Maliha Rahman, Jenny Zhang
EPI_ISL_925233	New Mexico Department of Health Scientific Laboratory	New Mexico Department of Health Scientific Laboratory	Ellie Johnson, Anastacia Griego-Fisher, D'eldra Malone, Jennifer Benoit
EPI_ISL_925719, EPI_ISL_925720, EPI_ISL_925816	Public Health Ontario Laboratory	Public Health Ontario Laboratory	Vanessa G Allen, Philip Banh, Yao Chen, Richard de Borja, Alireza Eshaghi, Nahuel Fittipaldi, Christine Frantz, Jonathan B Gubbay, Jennifer L Guthrie, Lawrence Heisler, Esha Joshi, Michael Laszloffy, Aimin Li, Michael CY Li, Dean Maxwell, Sandeep Nagra, Samir N Patel, Jared Simpson, Karthikeyan Sivaraman, Ashleigh Sullivan, Yogi Sundaravadanam, Sarah Teatero, Matthew Watson, Andre Villegas, Sandra Zittermann
EPI_ISL_930614, EPI_ISL_930631	University of Liège COVID-19 testing center	GIGA Medical Genomics	Keith Durkin, Maria Artesi, Bouchra Boujemla, Emmanuel André, Marc Van Ranst, Fabrice Bureau, Laurent Gillet, Wouter Coppieters, Vincent Bours
EPI_ISL_941282	Nigeria Centre for Disease Control (NCDC)	African Centre of Excellence for Genomics of Infectious Diseases (ACEGID), Redeemer's University	Oluniyi P.E. et al
EPI_ISL_942895	South Eastern Area Laboratory Services (SEALS)	NSW Health Pathology - Institute of Clinical Pathology and Medical Research; Westmead Hospital; University of Sydney	CIDM-PH et al.
EPI_ISL_943439	Dutch COVID-19 response team	National Institute for Public Health and the Environment (RIVM)	Adam Meijer, Harry Vennema, Dirk Eggink, Jeroen Cremer, Sharon van den Brink, Bas van der Veer, AnneMarie van den Brandt, Florian Zwagemaker, Dennis Schmitz, Chantal Reusken, on behalf of the national COVID-19 response team
EPI_ISL_944658, EPI_ISL_944659, EPI_ISL_944660	Department of Biochemistry, Cell and Molecular Biology, West African Centre for Cell Biology of Infectious Pathogens (WACCBIP), University of Ghana	Department of Biochemistry, Cell and Molecular Biology, West African Centre for Cell Biology of Infectious Pathogens (WACCBIP), University of Ghana	Morang'a,C.M., Ngoi,J.M., Quansah,E.B., Saiid,S., Amuzu,D.S., Asante,I., Bonney,J.H., Bonney,E., Odoom,J.K., Ndam,N.T., Tei-Maya,F., Adusei-Poku,M., Ofori-Boadu,L., Ampofo,W.K., Amenga-Etego,L.N., Quashie,P., Bediako,Y., Awandare,G.A.
EPI_ISL_949073, EPI_ISL_949075, EPI_ISL_949076	Jessa	Jessa	Jessa_cmdLab
EPI_ISL_953663	University Hospitals of Geneva, Laboratory of Virology	HUG, Laboratory of Virology and the Health2030 Genome Center	Samuel Cordey, Ana Rita Goncalves, Laurent Kaiser, Lorenzo Cerutti, Henri Pegeot, Melyssa Elies, Deborah Penet, Keith Harshman, Ioannis Xenarios, Emmanouil Dermitzakis
EPI_ISL_961171, EPI_ISL_961175	Texas Department of State Health Services	Texas Department of State Health Services	Bonnie Oh, Anita Pokharel, James Daniel Bonser, Myong Koag, Chung Wang, Rachel Lee, Grace Kubin, Rashmi Tuladhar, Mayela Pedrueza, Maliha

			Rahman, Jenny Zhang
EPI_ISL_965471, EPI_ISL_965472, EPI_ISL_965473	Synlab	GIGA Medical Genomics	Keith Durkin, Maria Artesi, Sébastien Bontems, Raphaël Boreux, Bouchra Boujemla, Cécile Meex, Pierrette Melin, Marie-Pierre Hayette, Vincent Bours
EPI_ISL_965871, EPI_ISL_965872	Massachusetts State Public Health Laboratory	Massachusetts State Public Health Laboratory	Andrew Lang, Timelia Fink, Glen Gallagher, Sandra Smole
EPI_ISL_967768	State Laboratories Division, Hawaii State Department of Health	State Laboratories Division, Hawaii State Department of Health	Pamela O'Brien, Drew Kuwazaki, Ayana Garnet, Razvan Sultana, Edward Desmond
EPI_ISL_977974	Chiu Laboratory, University of California, San Francisco	Chiu Laboratory, University of California, San Francisco	Charles Chiu, Xianding (Wayne) Deng, Candace Wang, Venice Servellita, Jill Hacker, Debra Wadford
EPI_ISL_981088, EPI_ISL_981097, EPI_ISL_981127, EPI_ISL_981132	Johns Hopkins Hospital Department of Pathology	Johns Hopkins Hospital Department of Pathology	C. Paul Morris, Chun Huai Luo, Adannaya Amadi, Matthew Schwartz, Nicholas Gallagher, Heba H. Mostafa
EPI_ISL_982467, EPI_ISL_982483	MONTEFIORE MEDICAL CENTER LABORATORIES	Wadsworth Center, New York State Department of Health	Kirsten St. George, Daryl M. Lamson, Alexis Russel, Matthew Shudt, Melissa A Leisner, Jonathan Plitnick, Navjot Singh, John Kelly, Erasmus Schneider, Erica Lasek-Nesselquist
EPI_ISL_983250	KALEIDA CENTER FOR LABORATORY MEDICINE	Wadsworth Center, New York State Department of Health	Kirsten St. George, Daryl M. Lamson, Alexis Russel, Matthew Shudt, Melissa A Leisner, Jonathan Plitnick, Navjot Singh, John Kelly, Erasmus Schneider, Erica Lasek-Nesselquist
EPI_ISL_984774, EPI_ISL_984818, EPI_ISL_984819, EPI_ISL_984899, EPI_ISL_994885, EPI_ISL_995083	Pandemic Response Lab - NYC	Pandemic Response Lab, R&D	Henry Lee, Michael Hammerling, Melissa Hopkins, Cybill del Castillo, William Ward, Pradeep Bugga, Haiping Hao, Jon Laurent
