# Supplementary material for: Detection of R.1 lineage severe acute respiratory syndrome coronavirus 2 (SARS-CoV-2) with spike protein W152L/E484K/G769V mutations in Japan
Source: PLoS Pathog. 2021 Jun 7;17(6):e1009619. doi: 10.1371/journal.ppat.1009619 (PMC8238201; doi:10.1371/journal.ppat.1009619)
Supplement: Attachment — Submitted filename: Revise_letter.docx [file ppat.1009619.s004.docx]

Dr. Kasturi Haldar and Dr. Michael Malim

Editor-in-Chief

*PLOS Pathogens*

30 April 2021

Dear Drs. Haldar and Malim,

Please find attached a revised version of our manuscript “Detection of R.1 lineage severe acute respiratory syndrome coronavirus 2 (SARS-CoV-2) with spike protein W152L/E484K/G769V mutations in Japan,” (Manuscript Number: PPATHOGENS-D-21-00692), which we would like to submit for publication as a Research Article in *PLOS Pathogens*.

With the addition of new data and the discovery of cases other than familial transmission, I would like to change the title from “Household transmission of SARS-CoV-2 R.1 lineage with spike E484K mutation in Japan” to new one.

We would like to thank the editor and reviewers for their quite constructive and helpful comments. Following these advices, we revised the manuscript. We hope that this revision makes our manuscript suitable for publication in *PLOS Pathogens*. The modified part will be kept in the Word change history in the revised manuscript.

Yours sincerely,

Yosuke Hirotsu, Ph.D.

**Responses to the comments of Editor**

**＜Comment＞**

Please address the comments of both reviewers constructively. We concur that addition of a well-constructed phylogenetic tree to show that the R1 lineage forms a monophyletic clade would be important (Reviewer #2 comments).

**＜Response＞**

Thank you very much for your comments on our paper. In accordance with the comment from the Reviewer 2, we have added a new figure to show that the R.1 lineage forms a monophyletic group.

**＜Comment＞**

This editor also suggests that the authors discuss the potential significance of the W152L mutation. Note that W152C is a defining mutation in B.1.429, an emerging Variant of Interest, and is located in the NTD antigenic supersite (PMID: 33761326, 33821281).

**＜Response＞**

According to the editor's comment, we discuss the potential significance of the W152L mutation and note that the W152C was identified in B1.429, which is defined as an emerging Variant of Interest.

**＜Comment＞**

In addition, please ensure that the manuscript is carefully proof-read to correct for grammar, spelling and syntax.

**＜Response＞**

In order to correct the English grammar, I request a professional English editing company to have it proofread by native English speakers. A proof of English editing will be attached to the manuscript at the time of submission.

**＜Comment＞**

Some incomplete sentences (e.g line 152-154) detract from a major point that the authors might want to make. With regards to the Japanese sequence data in Fig. 2B, please update data to Mar 1 if available.

**＜Response＞**

I have also updated the data using GISAID database as of April 22, 2021. At the time of the initial submission, it was ambiguous that R.1 lineage would increase in the future, but I have now confirmed that R.1 lineage are expanding in Japan. These observations were confirmed in our district and add new figure in revised manuscript.

**Responses to the comments of Reviewer #1**

**＜Comment＞**

Reviewer #1: Epidemiological event of R.1 lineage section should discuss sampling bias.

**＜Response＞**

I completely agree with your comments. I would like to discuss about the sampling bias.

**Responses to the comments of Reviewer #2**

**＜Comment＞**

Reviewer #2: I believe this paper is a good description of the R.1 lineage, and useful to those who wish to identify it and understand its initial distribution and spread. However, I think this paper would benefit greatly from even a very basic phylogenetic tree of the lineage and the sequences it covers. I think this would allow even a quick look at the relationships between the global sequences, and to show that this is indeed one monophyletic clade.

From looking at this clade on the focal Nextstrain 484K build (https://nextstrain.org/groups/neherlab/ncov/S.E484?c=country&f_pango_lineage=R.1&label=mlabel:20B/C18877T), it does indeed seem to be, but sometimes in the paper it seems like perhaps this is presented as ambiguous (though this may not have been intentional, and I may be misinterpreting the writing - apologies if so). For example in lines 147-148, saying "...implying that [the lineage] may have emerged in several regions at approximately the same time."

**＜Response＞**

These comments are very constructive comments. As your suggestion, we analyzed the phylogenetic tree of global data and add new figure in revised manuscript. The R.1 lineage consist of one monophyletic clade. As for the ambiguously worded sentence, we have clarified that those with the ORF1b G814C mutation occur in Austria and the United States, while those without the mutation are increasing in countries including Japan.

**＜Comment＞**

Further a phylogeny would help to clarify the meaning of statement like in lines 168-169: "The circulating trend of R.1 lineage showed similar pattern (note: typo, should be 'patterns') in each country, implying that the ancestral strain acquired homegrown variants, and subsequently R.1 lineage are likely to emerge in several countries." Is there evidence of unique R.1 variants in different countries? (I actually think from the Nextstrain tree, there may be - but showing a tree would display this more clearly, if so.)

**＜Response＞**

As you pointed out, the rationale for this point was ambiguous, so it has been removed in the revised manuscript.

**＜Comment＞**

I think even a fairly basic phylogeny as an additional figure, with a small additional description of what can be seen, would add a lot of value to the paper in showing any clusters in any countries, the relationship between USA & Japanese sequences, and the genetic diversity of the cluster (including any sub-clusters within the lineage with S or other AA mutations).

**＜Response＞**

In the revised version, the phylogenetic tree has been added as a new diagram, and the relationship between Japan and other countries has been clearly written.

Part III – Minor Issues

**Responses to the comments of Reviewer #1**

**＜Comment＞**

Reviewer #1: There are a number of minor typos / grammar problems.

**＜Response＞**

Thank you for pointing out typos and grammar problems. We requested a company that specializes in English editing to proofread the entire text by a native speaker.

**＜Comment＞**

I also did not see a supplemental data table to conform with the GISAID terms of use.

**＜Response＞**

I have acknowledged the institutions and laboratories from which the data originated. I have added new supplemental table data.

**Responses to the comments of Reviewer #2**

**＜Comment＞**

Reviewer #2: I understand the world of SARS-CoV-2 research moves incredibly quickly, but I fear by the time this work is published it may seem quite outdated, given the data was collected through Feb 2021. As the work is descriptive I do think much of the value lies in the descriptions being as up-to-date as possible, so I would urge the authors to update the paper (counts, global coverage, etc) to cover data through as recent a period as is possible. This would also possibly shed some clarity onto statements like that of 153-154 ("it is possible that R.1 lineage is gradually increasing."

**＜Response＞**

Thank you for your constructive comments. In order to make the data more up-to-date, we have performed the analysis based on the data registered in GISAID as of April 22, 2021. As you pointed out, we confirmed that the R.1 lineage have been increasing in Japan. In order to make this clear, we have changed the description.

**＜Comment＞**

Minor typos/wording: Line 170 "acquired" should be "acquires". Line 174, should be "therefore, new emerging strain[s] [possibly] have been missed."

**＜Response＞**

Thank you pointing our typo. I modify these sentence. I also requested a company that specializes in English editing to proofread the entire text by a native speaker.
